# Antiseptic Cleansing to Reduce Vertical Transmission of Pathogens to Neonates: The NeoVT-AMR Randomized Clinical Trial

**DOI:** 10.1001/jamanetworkopen.2026.15665

**Published:** 2026-06-11

**Authors:** Emily Beales, Michelle N. Clements, Nicholas A. Feasey, David Lissauer, Maranatha Banda, Bertha Maseko, Julia A. Bielicki, Samantha Lissauer, Aisleen Bennett, Kondwani Kawaza, Luis A. Gadama, A. Sarah Walker, Mike Sharland, Louise F. Hill

**Affiliations:** 1 Centre for Neonatal and Paediatric Infection, Institute for Infection and Immunity, City St George’s, University of London, London, United Kingdom; 2 University College London Innovative Clinical Trials Unit, London, United Kingdom; 3 School of Medicine, University of St Andrews, St Andrews, United Kingdom; 4 Malawi-Liverpool-Wellcome Programme, Blantyre, Malawi; 5 Institute of Life Course and Medical Sciences, University of Liverpool, Liverpool, United Kingdom; 6 Zomba Central Hospital, Zomba, Malawi; 7 Paediatric Research Centre, University Children’s Hospital, Basel, Basel, Switzerland; 8 Institute of Infection, Veterinary and Ecological Sciences, University of Liverpool, Liverpool, United Kingdom; 9 Department of Paediatric Infectious Diseases, Imperial College Healthcare, NHS Trust, London, United Kingdom; 10 Paediatric Infectious Disease, Department of Infectious Disease, Imperial College, London, United Kingdom; 11 Department of Paediatrics and Child Health, Kamuzu University of Health Sciences, Blantyre, Malawi; 12 Obstetrics and Gynaecology Department, Kamuzu University of Health Sciences, Blantyre, Malawi

## Abstract

**Importance:**

Neonatal sepsis causes substantial mortality. Topical antisepsis for laboring women or neonates may reduce pathogenic colonization and sepsis risk.

**Objective:**

To evaluate the safety and effectiveness of various topical antiseptic regimens to reduce bacterial load in the maternal genital tract and on neonatal skin and assess suitability for future effectiveness trials.

**Design, Setting, and Participants:**

This randomized clinical trial was conducted from March 7, 2022, to March 29, 2023, at Zomba Central Hospital, Malawi, with 28-day follow-up. Participant populations were laboring women and, separately, facility-born neonates (aged <24 hours and with birth weight >1000 g) not born to a mother recruited to the trial. Data were analyzed from May 17 to December 28, 2023.

**Interventions:**

Participants were individually randomized in an unblinded factorial design to chlorhexidine 1% (1% CHG), chlorhexidine 2% (2% CHG), or octenidine 0.1% with phenoxyethanol 2% (OHP), each applied either once or multiple times (maternal: antiseptic applied every 4 hours during working hours for up to 6 applications; neonatal: antiseptic applied every 24 hours for up to 3 applications), or to standard of care (SOC; application of sterile water for mothers and no cleansing for neonates). Laboratory staff assessing primary outcomes were blinded.

**Main Outcomes and Measures:**

Co–primary outcomes were change in total skin bacterial load (log10 colony-forming units [log10CFU]) from baseline at each follow-up, analyzed separately in the maternal and neonatal populations and adjusted for intervention variables. Secondary outcomes included skin condition score (range, 0-12 for neonates and 0-16 for women; lower scores indicate better condition), serious adverse events (SAEs), and neonatal temperature.

**Results:**

A total of 149 women (mean [SD] age at enrollment, 25.7 [5.9] years) and 147 neonates (mean [SD] age at enrollment, 10.3 [6.3] hours; 82 [56%] male) participated. Mean (SD) infant gestational age was 37.7 (1.5) weeks in the maternal population and 36.7 (3.1) weeks in the neonatal population. Neonates’ mean birth weight was 2729 g (712 g). Among mothers, compared with 1% CHG, bacterial load was higher (worse) with OHP (adjusted log10CFU difference, 1.7; 95% CI, 0.9-2.5) and SOC (3.5; 95% CI, 2.4-4.6); there was no clear log10CFU difference with 2% CHG (−0.6; 95% CI, −1.4 to 0.2). There was no evidence of difference in effectiveness between multiple vs single application (log10CFU difference, −0.4; 95% CI, −1.1 to 0.2). In neonates, 1% CHG showed greater effectiveness than SOC (log10CFU difference, 1.3; 95% CI, 0.2-2.4) but no difference vs 2% CHG (−0.2; 95% CI, −1.1 to 0.7) or OHP (0.7; 95% CI, −0.2 to 1.6). Multiple applications showed increasing benefits over time (frequency × time interaction). Skin scores were low (almost all were 0-1 and none ≥3). There were no significant differences in SAE rates between arms and no signal of postantiseptic neonatal hypothermia.

**Conclusions and Relevance:**

In this randomized clinical trial of topical antiseptics applied in laboring women and in neonates, 1% CHG reduced maternal and neonatal bacterial colonization without safety concerns, suggesting it would be the optimal regimen to evaluate in a larger pragmatic trial powered for clinical outcomes.

**Trial Registration:**

ISRCTN Registry Identifier: ISRCTN78026255

## Key Points

**Question:**

Is topical antiseptic application in laboring women and neonates safe and effective at reducing bacterial load?

**Findings:**

In this randomized clinical trial including 149 women in labor and 147 neonates in Malawi, application of 1% chlorhexidine reduced bacterial load compared with standard care in both populations, with larger effects in the mothers. No safety concerns were identified.

**Meaning:**

The findings suggest application of 1% chlorhexidine in neonates and laboring women could be incorporated into future multimodal infection-prevention intervention trials in low-income countries.

## Teaser

This randomized clinical trial compares the effectiveness and safety of topical antiseptic regimens to reduce bacterial load in the maternal genital tract and on neonatal skin for prevention of sepsis in low-income countries.

## Introduction

Infection is a major cause of neonatal mortality, with serious bacterial infections responsible for approximately 680 000 neonatal deaths per year in low-income countries (LICs).^1^ Gram-negative pathogens, such as *Klebsiella* species and *Escherichia coli*, commonly cause early-onset sepsis (EOS; within 72 hours of birth^2,3^), which can be acquired vertically from the maternal genital tract during labor.^4^ Affordable strategies to reduce serious bacterial infections in neonates are urgently required.

Antiseptics, applied vaginally during labor or topically to the skin of neonates, offer a potential preventive approach. Chlorhexidine (CHG) and octenidine with phenoxyethanol (OHP) are inexpensive, widely used, and well tolerated. CHG is routinely used worldwide before procedures in neonatal care to reduce skin bacterial load,^5^ and OHP is used in Europe to treat bacterial vaginosis and in neonatal units.^6^ Preclinical studies suggest higher activity of octenidine than CHG against gram-negative organisms.^7,8^

Evidence for clinical benefit is mixed. A South African trial of 0.5% CHG wipes found no reduction in EOS,^9^ whereas nonrandomized studies of CHG vaginal cleansing reported reduced neonatal infection–related mortality.^10,11^ Optimal CHG concentration remains uncertain,^12^ and evidence for preventing non–Group B *Streptococcus* EOS is limited.^13^

Maternal outcomes are also relevant, with obstetric infections accounting for approximately 11% of maternal deaths^14^ and CHG vaginal cleansing reducing postoperative infectious morbidity after cesarean delivery.^15,16^ Large pragmatic trials are required to determine whether maternal or neonatal antiseptic use can reduce neonatal sepsis and maternal infections in moderate- to high-incidence settings and which antiseptic and dosing strategy should be prioritized. This trial evaluated topical antiseptic strategies in laboring women and newborns to reduce bacterial load in the maternal genital tract and on neonatal skin, aiming to identify the most promising regimen for a future pragmatic trial to prevent neonatal sepsis in LICs.

## Methods

### Study Design and Oversight

**Figure 1.**
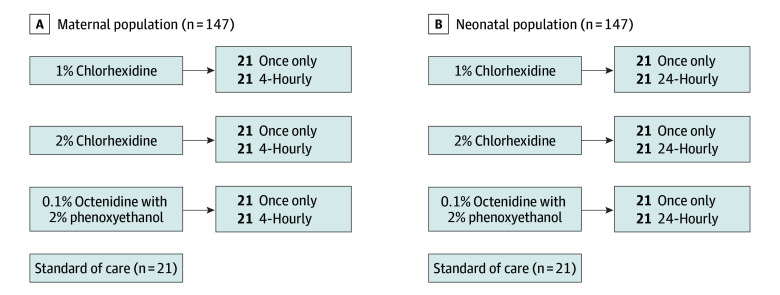
Diagram of NeoVT-AMR Trial Schema

Strategies to reduce vertical transmission of multi–drug-resistant pathogens to neonates (NeoVT-AMR) was an unblinded, factorial, individually randomized clinical trial with 2 independent participant populations: laboring women (maternal population) and neonates (neonatal population). Mothers and neonates were allocated separately in a (3 × 2) + 1 factorial design comprising 6 intervention arms and 1 standard of care (SOC) arm (Figure 1). The factorial structure allowed simultaneous superiority comparisons of 3 maternal or neonatal antiseptic regimens (1% CHG, 2% CHG, and OHP [octenidine 0.1% with phenoxyethanol 2%]) and 2 application strategies (once only vs multiple applications), with the SOC arm serving as a benchmark to distinguish between scenarios in which all experimental regimens were similarly effective or ineffective. The trial protocol is available in Supplement 1. The trial was registered with ISRCTN and received ethical approval from the research ethics committees of St George’s, University of London and the Kamuzu University of Health Sciences, Blantyre, Malawi, as well as regulatory approval from the Pharmacy and Medicines Regulatory Authority of Malawi. Written informed consent was obtained from all women and all parents or guardians of neonates. A data monitoring committee assessed safety end points halfway through recruitment. The study was performed in accordance with the International Council for Harmonisation of Technical Requirements for Pharmaceuticals for Human Use Guideline for Good Clinical Practice.^17^ This study followed the Consolidated Standards of Reporting Trials (CONSORT) reporting guideline.^18^

### Participants

The trial was conducted from March 7, 2022, to March 29, 2023, at Zomba Central Hospital, Zomba, Malawi, in the antenatal and labor wards (maternal population) and the neonatal and postnatal wards (neonatal population). Eligible mothers were those in early labor, with or without rupture of membranes, at the time of randomization. Important exclusion criteria were active labor, elective cesarean delivery, and intrauterine death.

Eligible neonates were those born in a health care facility, aged less than 24 hours, and with birth weight greater than 1000 g (as gestational age was not always known). Important neonatal exclusion criteria were birth to a mother also recruited to the trial (in case maternal interventions masked neonatal effects), birth by elective cesarean delivery, and poor skin condition (skin score ≥2 on a scale from 0-12 in any of the 3 domains of dryness, erythema, and skin breakdown, with higher scores indicating poorer skin condition) (eMethods 1 in Supplement 2). Neonatal criteria were amended from age of less than 12 hours to less than 24 hours and from birth at Zomba Central Hospital to birth in a facility to support enrollment (eAppendix in Supplement 2).

Randomization used a computer-generated sequence created by M.N.C. (blocks of 13) and implemented by the trial staff via REDCap. Staff and participants were unblinded, but laboratory assessors of co–primary outcomes were blinded.

### Study Procedures

In both populations, the intervention arms assessed 3 antiseptics (1% CHG, 2% CHG, or OHP), applied once only (at randomization) or multiple times. All participants except those in the SOC arms had antiseptic application at baseline. Maternal antiseptic application was to the vagina and perineum with cotton wool. Maternal multiple-application arms received antiseptic every 4 hours during the day for up to 6 applications (32 hours from randomization) or until delivery; the SOC arm received application of sterile water applied 4-hourly before a vaginal examination per the World Health Organization (WHO) recommendation^19^ (eMethods 2 in Supplement 2). Neonatal antiseptic application was to the body (not face and scalp). Neonatal multiple-application arms received antiseptic every 24 hours for up to 4 applications (72 hours from randomization) or until discharge; the SOC arm received no cleanses.

Swab samples were taken regularly from all nondischarged participants and before any antiseptic application: every 4 hours during the day (to 32 hours) in the maternal population (vagina and perineum) and every 24 hours (to 72 hours) in the neonatal population (neck skin fold and perirectal). Samples in Amies medium (Transwab Amies [Medical Wire & Equipment]) were vortexed, diluted, plated on UriSelect medium and blood agar (Bio-Rad Laboratories), and incubated overnight before colonies were counted (eMethods 3 in Supplement 2).

### Outcomes

The co–primary outcomes were change from baseline to each swab sample time point in total bacterial load in log10 colony forming units (log10CFU) in the maternal and neonatal populations separately. Key secondary outcomes in both populations were safety by skin condition score (measured at baseline and at each treatment time point) (eMethods 1 in Supplement 2) and serious adverse events (SAEs) to day 28 (eMethods 4 and 5 in Supplement 2). A temperature safety outcome in the neonatal population was assessed, both as temperature change from before to after antiseptic application and as a graded measurement of hypothermia (daily for those in the SOC arm). A secondary outcome in the maternal population assessed bacterial load (neck skin fold and perirectal) in neonates born to the enrolled women. In the case of a multiple birth, swab samples were taken from the firstborn. Additional effectiveness outcomes specified in the statistical analysis plan were log10CFU of gram-positive, gram-negative, and yeast species (Supplement 1).

### Statistical Analysis

A sample of 147 participants per population gave 90% power to detect a difference of 0.82 SDs between antiseptics (α = .012), 0.58 SDs between application frequencies (α = .05), and 1.01/0.90 SDs compared with SOC (α = .012/.025). At 80% power, detectable effects were 0.72, 0.50, 0.88, and 0.79 SDs. These targets were considered adequate based on prior log10CFU reductions (0.2-2 SDs).^5,20^

Analyses followed the intention-to-treat (treatment-policy) estimand, with populations analyzed separately. Participants with a baseline measure and at least 1 postbaseline measure were included, and analyses used data from all available time points. Total change in log10CFU was analyzed using mixed-effects linear models with fixed effects for antiseptic, application frequency, assessment time, and baseline log10CFU and a random-participant effect. SOC was a benchmark only; the reference category was a single application of 1% CHG. Early discharge explained loss to follow-up and indicated the feasibility of multiple applications. Statistical significance was set at 2-sided *P* < .05, with no multiplicity adjustment owing to the exploratory design.

Interaction and prespecified subgroup analyses were run separately, with sensitivity analyses using only the first and last postbaseline measures. Bayesian ACCEPT analysis^21^ estimated differences from the reference category (eMethods 6 in Supplement 2). Skin score and neonatal temperature changes were analyzed similarly. Adverse events (AEs) and SAEs were summarized and compared using exact logistic regression. Analyses were performed from May 17 to December 28, 2023, in Stata, version 17 (StataCorp LLC).

## Results

**Figure 2.**
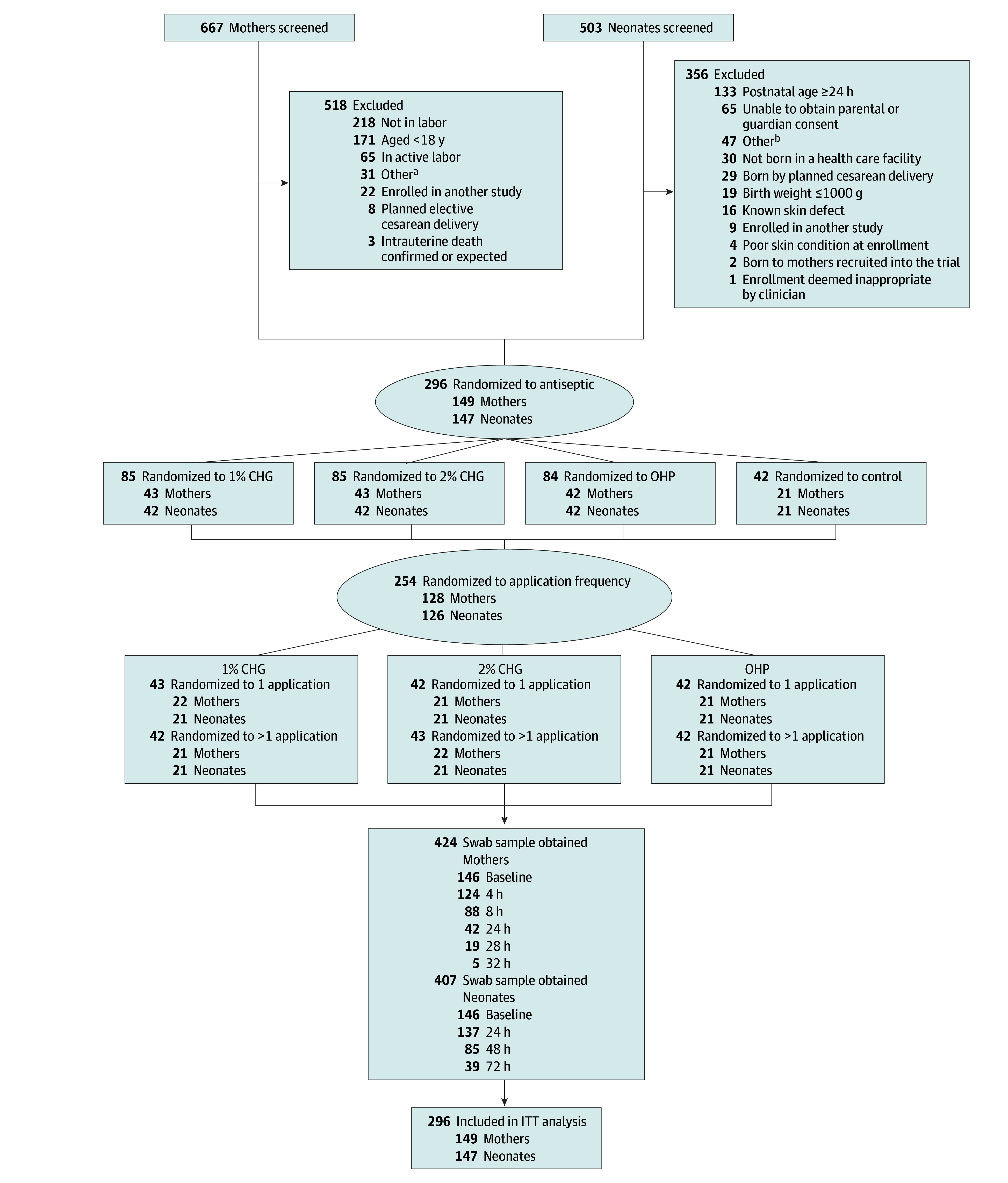
CONSORT Diagram of Participant Flow Through Trial CHG indicates chlorhexidine; ITT, intention to treat; OHP, octenidine with phenoxyethanol. ^a^Other reasons for maternal exclusions were not being in the ward (n = 10), deferred decision pending discussion with others (n = 10), preterm delivery (n = 4), not responding (n = 3), in extreme pain (n = 3), and laboratory unable to receive samples (n = 1). ^b^Other reasons for neonatal exclusions were planned discharge (n = 20), deferred decision pending discussion with others (n = 15), neonate was very sick (n = 8), mother was very sick (n = 2), and online database was not working (n = 2).

A total of 667 mothers were screened (149 enrolled) and 503 neonates were screened (147 enrolled) (Figure 2). Recruitment ended when the target of 147 was reached in each population.

At enrollment, the mean (SD) maternal age was 25.7 (5.9) years and mean (SD) neonatal age was 10.3 (6.3) hours (Table 1 and eTables 1 and 2 in Supplement 2). Estimated mean (SD) infant gestational age at labor was 37.7 (1.5) weeks in the maternal population and 36.7 (3.1) weeks in the neonatal population. Mean (SD) neonatal birth weight was 2729 g (712 g); 64 (44%) were female, 82 (56%) were male, and 1 (1%) had unknown sex; and 76 (52%) were born by spontaneous vaginal delivery vs 61 (41%) by emergency cesarean delivery.

**Table 1.** Participant Baseline Characteristics^a^

Characteristic	Overall	Antiseptic			Application frequency		SOC
		1% CHG	2% CHG	OHP	Once	Multiple	
**Maternal population**							
Mothers, No.	149	43	43	42	64	64	21
Age at randomization, mean (SD), y	25.7 (5.9)	26.3 (6.6)	25.4 (6.1)	25.7 (5.6)	27.5 (6.6)	24.1 (5.0)	24.8 (5.0)
Estimated gestational age at labor, mean (SD), wk	37.7 (1.5)	37.8 (1.3)	37.8 (1.6)	37.4 (1.4)	37.9 (1.2)	37.5 (1.6)	37.6 (1.9)
Antenatal care during pregnancy	144 (97)	43 (100)	43 (100)	37 (88)	62 (97)	61 (95)	21 (100)
Tuberculosis	2 (1)	0	1 (2)	1 (2)	0	2 (3)	0
HIV	14 (9)	5 (12)	4 (9)	4 (10)	10 (16)	3 (5)	1 (5)
Antibiotics							
During pregnancy	26 (17)	5 (12)	10 (23)	9 (21)	13 (20)	11 (17)	2 (10)
Since admission	5 (3)	3 (7)	0	2 (5)	3 (5)	2 (3)	0
Total log10CFU at baseline, mean (SD)	6.3 (2.0)	6.2 (1.9)	6.3 (1.9)	6.6 (2.1)	6.4 (1.7)	6.3 (2.2)	6.0 (2.1)
**Neonatal population^b^**							
Neonates, No.	147	42	42	42	63	63	21
Age at enrollment, mean (SD), h	10.3 (6.3)	10.8 (5.9)	10.6 (6.6)	9.4 (6.0)	10.3 (6.1)	10.3 (6.3)	10.6 (7.2)
Estimated gestational age at labor, mean (SD), wk	36.7 (3.1)	36.9 (2.9)	36.6 (3.1)	37.0 (3.1)	36.5 (3.0)	37.2 (3.0)	36.0 (3.1)
Sex							
Female	64 (44)	17 (40)	21 (50)	17 (40)	26 (41)	29 (46)	9 (43)
Male	82 (56)	24 (57)	21 (50)	25 (60)	37 (59)	33 (52)	12 (57)
Unknown	1 (1)	1 (2)	0	0	0	1 (2)	0
Birth weight, mean (SD), g	2729 (712)	2661 (671)	2751 (731)	2877 (724)	2739 (690)	2787 (732)	2528 (713)
Where admitted							
Nursery	50 (34)	14 (33)	17 (40)	13 (31)	24 (38)	20 (32)	6 (29)
Nursery HDU	50 (34)	13 (31)	11 (26)	16 (38)	20 (32)	20 (32)	10 (48)
Postnatal ward with mother	46 (31)	14 (33)	14 (33)	13 (31)	19 (30)	22 (35)	5 (24)
Unknown	1 (1)	1 (2)	0	0	0	1 (2)	0
Reason for admission							
Birth asphyxia	48 (33)	13 (31)	12 (29)	16 (38)	20 (32)	21 (33)	7 (33)
Other	54 (37)	15 (36)	17 (40)	13 (31)	25 (40)	20 (32)	9 (43)
Routine admission	44 (30)	13 (31)	13 (31)	13 (31)	18 (29)	21 (33)	5 (24)
Unknown	1 (1)	1 (2)	0	0	0	1 (2)	0
Any antibiotics prescribed before enrollment since birth							
Yes	31 (21)	8 (19)	7 (17)	10 (24)	12 (19)	13 (21)	6 (29)
No	115 (78)	33 (79)	35 (83)	32 (76)	51 (81)	49 (78)	15 (71)
Unknown	1 (1)	1 (2)	0	0	0	1 (2)	0
Chlorhexidine cord care given							
Yes	112 (76)	32 (76)	32 (76)	33 (79)	50 (79)	47 (75)	15 (71)
No	34 (23)	9 (21)	10 (24)	9 (21)	13 (21)	15 (24)	6 (29)
Unknown	1 (1)	1 (2)	0	0	0	1 (2)	0
Mother received antibiotics in labor							
Yes	59 (40)	16 (38)	20 (48)	18 (43)	29 (46)	25 (40)	5 (24)
No	77 (52)	25 (60)	19 (45)	21 (50)	31 (49)	34 (54)	12 (57)
Unknown	11 (7)	1 (2)	3 (7)	3 (7)	3 (5)	4 (6)	4 (19)
Total log10CFU at baseline, mean (SD)	4.3 (2.9)	4.2 (2.8)	4.3 (3.1)	3.9 (2.8)	3.9 (2.5)	4.4 (3.2)	5.3 (3.4)

Abbreviations: CFU, colony-forming units; CHG, chlorhexidine; HDU, high dependency unit; OHP, octenidine with phenoxyethanol; SOC, standard of care.

^a^ Data are presented as number (percentage) of participants unless otherwise indicated. Individuals assigned to a non-SOC arm appear twice in the table: in the randomized antiseptic analysis and the application frequency analysis.

^b^ One neonate in the neonatal population was withdrawn before any baseline data were collected and is shown as “unknown.”

Protocol adherence was high: no SOC participants received antiseptic and no single-application participants received multiple applications (eTables 3 and 4 in Supplement 2). The median number of applications in the multiple-application arm was 3 (IQR, 2-3) in both populations. Follow-up at day 28 was achieved for 104 mothers (70%) and 114 neonates (78%).

In the maternal population, 146 participants (98%) had a swab sample obtained at baseline, 124 (83%) at 4 hours, and 88 (59%) at 8 hours—the point at which differences between frequency arms may have begun to appear; 5 mothers (3%) remained undelivered for the 32-hour swab sample (Figure 2). In the neonatal population, 146 (99%) had a swab sample obtained at baseline, 137 (93%) at 24 hours, 85 (58%) at 48 hours, and 39 (27%) at 72 hours. Decreasing numbers at later time points reflected women giving birth and routine neonatal discharge, respectively, rather than study-related loss to follow-up.

**Figure 3.**
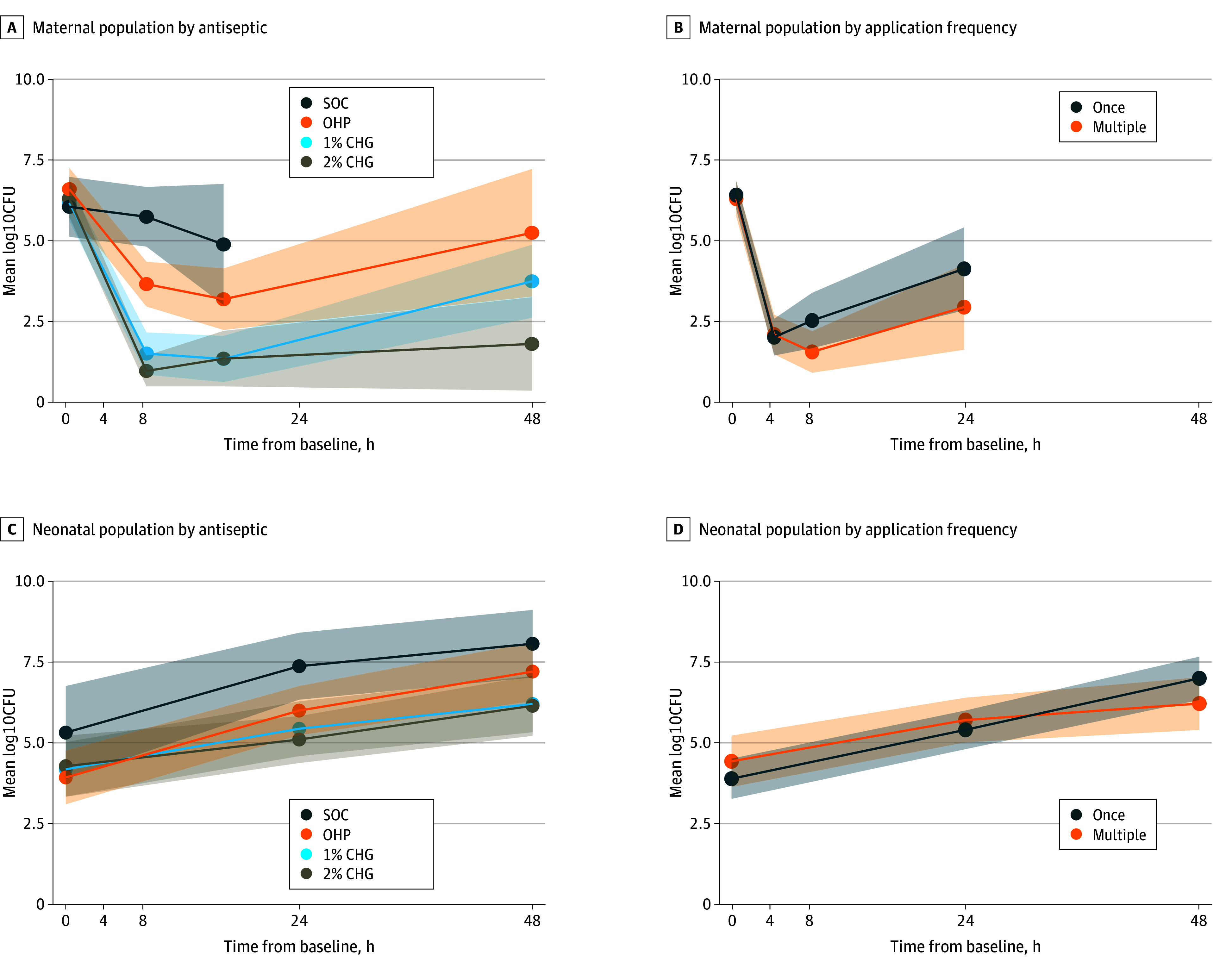
Line Graphs Showing Bacterial Load Over Time in the Maternal and Neonatal Populations Data from each non–standard of care (SOC) factorial arm (antiseptic or frequency) were averaged across the other factorial arm (frequency or antiseptic, respectively); thus, participants in each antiseptic arm appear twice (in the antiseptic and frequency graphs). Data points comprising fewer than 10 participants were removed for clarity. Time 0 corresponds to enrollment. Shading represents 95% CIs. CFU indicates colony-forming units; CHG, chlorhexidine; OHP, octenidine with phenoxyethanol.

In the maternal population, bacterial load was lower (better) with 1% CHG than with OHP (log10CFU difference, 1.7; 95% CI, 0.9-2.5; *P* < .001) or SOC (log10CFU difference, 3.5; 95% CI, 2.4-4.6; *P* < .001) (Table 2, Figure 3, and eTables 5 and 6 and eFigures 1 and 2 in Supplement 2). There was no evidence of a difference between 1% CHG and 2% CHG (log10CFU difference, −0.6; 95% CI, −1.4 to 0.2; *P* = .12). There was also no evidence of an effect of application frequency (multiple vs single: log10CFU difference, −0.4; 95% CI, −1.1 to 0.2; *P* = .20). There was no evidence of treatment interactions or subgroup effects (eTables 7 and 9 in Supplement 2).

**Table 2.** Primary and Key Secondary Outcomes

Outcome	Maternal population			Neonatal population		
	Adjusted difference (95% CI)^a^	*P* value	*P* value (overall antiseptic effect)^b^	Adjusted difference (95% CI)^a^	*P* value	*P* value (overall antiseptic effect)^b^
**Total log10CFU^c^**						
2% CHG vs 1% CHG	−0.6 (−1.4 to 0.2)	.12	<.001	−0.2 (−1.1 to 0.7)	.63	.06
OHP vs 1% CHG	1.7 (0.9 to 2.5)	<.001		0.7 (−0.2 to 1.6)	.11	
SOC vs 1% CHG	3.5 (2.4 to 4.6)	<.001	NA	1.3 (0.2 to 2.4)	.02	NA
>1 vs 1 Application	−0.4 (−1.1 to 0.2)	.20		−0.5 (−1.2 to 0.2)	.17	
**Skin score, points^d^**						
2% CHG vs 1% CHG	0.16 (−0.03 to 0.35)	.09	.02	0.02 (−0.15 to 0.18)	.86	.90
OHP vs 1% CHG	0.26 (0.07 to 0.45)	.007		0.04 (−0.13 to 0.20)	.66	
SOC vs 1% CHG	−0.04 (−0.31 to 0.22)	.74	NA	−0.05 (−0.27 to 0.16)	.63	NA
>1 vs 1 Application	0.21 (0.06 to 0.36)	.008		0.02 (−0.12 to 0.16)	.78	
**log10CFU in neonates born to a mother in the maternal population^c^**						
2% CHG vs 1% CHG	−0.4 (−1.8 to 1.0)	.54	.02	NA	NA	NA
OHP vs 1% CHG	1.5 (0.1 to 2.9)	.04		NA	NA	
SOC vs 1% CHG	0.5 (−1.4 to 2.4)	.58	NA	NA	NA	NA
>1 vs 1 Application	0.0 (−1.2 to 1.1)	.99		NA	NA	
**Gram-negative log10CFU^c^**						
2% CHG vs 1% CHG	−0.1 (−0.8 to 0.6)	.72	.001	−0.1 (−1.1 to 0.8)	.79	.86
OHP vs 1% CHG	1.1 (0.4 to 1.8)	.002		−0.3 (−1.2 to 0.7)	.59	
SOC vs 1% CHG	2.1 (1.1 to 3.0)	<.001	NA	−0.9 (−2.1 to 0.3)	.14	NA
>1 vs 1 Application	−0.1 (−0.7 to 0.5)	.72		−0.1 (−0.8 to 0.7)	.87	

Abbreviations: CFU, colony-forming units; CHG, chlorhexidine; NA, not applicable; OHP, octenidine with phenoxyethanol; SOC, standard of care.

^a^ Values are adjusted differences from mixed-effects linear models, averaged over time points and adjusted for antiseptic, application frequency, assessment time, and baseline value; participant was a random effect.

^b^ Test for the antiseptic factor within each outcome.

^c^ Positive values indicate higher bacterial load (worse); negative values indicate lower load (better). The reference group was 1% CHG applied once only (effect = 0).

^d^ Higher skin score values indicate worse skin condition (maternal scale, 0-16 points; neonatal scale, 0-12 points).

In the neonatal population, there was no evidence of a difference in bacterial load between 1% CHG and 2% CHG (log10CFU difference, −0.2; 95% CI, −1.1 to 0.7; *P* = .63) or OHP (log10CFU difference, 0.7; 95% CI, −0.2 to 1.6; *P* = .11). Bacterial load was lower (better) with 1% CHG than with SOC (log10CFU difference, 1.3; 95% CI, 0.2 to 2.4; *P* = .02). There was no evidence of an effect of multiple vs single application frequency (log10CFU difference, −0.5; 95% CI, −1.2 to 0.2; *P* = .17). There was evidence of an interaction between frequency and time, with the benefit of multiple applications increasing over time, and there were further interactions with admission ward and prior antibiotics (eTables 8 and 10 in Supplement 2).

Bayesian ACCEPT analysis (eTables 11 and 12 in Supplement 2) provided little evidence that 2% CHG was better than 1% CHG overall, with probabilities of 94% in the maternal population and 63% in the neonatal population (reducing to 62% and 22%, respectively, when requiring a log10CFU improvement of at least 0.5). The probability of OHP or SOC outperforming 1% CHG was near 0 (0%-4%). The probability that multiple applications were better than a single application was 81% in the maternal and 82% in the neonatal population. Prespecified sensitivity analyses showed similar patterns (eTables 13 and 14 in Supplement 2).

Skin scores were low (good) in both populations, almost all being 0 or 1 (out of 16 in maternal and 12 in neonatal participants); only 3 of 277 maternal (1%) and 5 of 262 neonatal (2%) assessments reached a score of 2, and none were 3 or higher. In the maternal population (Table 2), skin scores were higher with OHP than with 1% CHG (difference, 0.26 points; 95% CI, 0.07-0.45 points; *P* = .007) and higher with multiple applications than a single application (difference, 0.21 points; 95% CI, 0.06-0.36 points; *P* = .008). There was no evidence of a significant difference between 1% CHG and 2% CHG (difference, 0.16 points; 95% CI, −0.03 to 0.35 points; *P* = .09) or between 1% CHG and SOC (difference, −0.04 points; 95% CI, −0.31 to 0.22 points; *P* = .74). In the neonatal population, there was no evidence of differences between arms in skin score or postapplication body temperature.

Seven SAEs occurred in 7 women (5% of the maternal population), 6 (86%) of which were related to the neonate, and 17 SAEs occurred in 16 neonates (11% of the neonatal population) (eTables 15 and 16 in Supplement 2). There was no evidence of differences in the proportion of participants experiencing an SAE between treatment arms in either population, although neonatal SAE counts were numerically higher in the 2% CHG arm. The most common SAE system organ class in the maternal population was “pregnancy, puerperium, and perinatal conditions,” occurring in 2 of 22 women (9%) in the arm receiving a single application of 1% CHG and 1 of 22 women (5%) in the arm receiving multiple applications of 2% CHG (eTable 17 in Supplement 2). The most common SAE system organ class in the neonatal population was “infections and infestations,” occurring in 10 neonates overall (7%): 3 of 21 (14%) in the arm receiving a single application of 2% CHG, 2 of 21 (10%) receiving multiple applications of 1% CHG, 2 of 21 (10%) receiving multiple applications of 2% CHG, 1 of 21 (5%) receiving a single application of 1% CHG, 1 of 21 (5%) receiving multiple applications of OHP, and 1 of 21 (5%) receiving SOC (eTable 18 in Supplement 2). No SAE was judged to be related to the antiseptic, and no skin reactions of grade 3 or 4 were reported. In the neonatal population, 2 temperatures less than 35 °C were recorded: 1 of 21 (5%) in the SOC arm and 1 of 21 (5%) at baseline in a participant in the single-application group receiving 1% CHG.

Among neonates born to mothers in the maternal population, patterns were similar to those seen in mothers: bacterial load was higher with OHP than with 1% CHG (log10CFU difference, 1.5; 95% CI, 0.1-2.9; *P* = .04) (Table 2), with no evidence of a difference between 1% CHG and 2% CHG (log10CFU difference −0.4; 95% CI, −1.8 to 1.0; *P* = .54). There was no evidence of an effect of application frequency (log10CFU difference for >1 vs 1 application, 0.0; 95% CI, −1.2 to 1.1; *P* = .99) or of a difference between 1% CHG and SOC (log10CFU difference, 0.5; 95% CI, −1.4 to 2.4; *P* = .58). Total bacterial load was higher in neonates born to mothers without vs with HIV and following spontaneous vaginal birth, although there was no evidence of interactions between these factors and treatment arms (eTable 9 in Supplement 2).

Results for gram-positive, gram-negative, and yeast organisms in both the maternal and neonatal populations were consistent with the primary bacterial-load findings, although reduced yeast colonization was seen in the maternal population receiving 2% CHG compared with 1% CHG and higher Group B *Streptococcus* colonization was seen in the neonatal population receiving OHP compared with 1% CHG. There were no substantive differences from the overall patterns described previously (eTables 5 and 6 and eFigures 1 and 2 in Supplement 2).

## Discussion

In this randomized clinical trial evaluating topical antiseptic strategies in laboring women and newborns, we aimed to identify an antiseptic regimen suitable for a future effectiveness trial to reduce neonatal sepsis in low-income settings. Using change in total bacterial load as a surrogate effectiveness outcome, the trial showed that 1% CHG was both safe and effective in reducing maternal and neonatal colonization. These findings support 1% CHG as the regimen most appropriate to take forward into a larger trial powered for clinical end points.

Lower total bacterial load was observed with 1% CHG applied once compared with SOC in both populations. Effect sizes were larger for the maternal population, likely reflecting the more frequent sampling and differences in anatomic site and environmental exposure. These findings align with previous studies in neonates^22,23,24,25,26^ and laboring women^27^ showing that CHG reduced colony counts, with neonatal rebound colonization commonly reported. There was no robust evidence that 2% CHG performed better than 1% CHG in either population, although reduced yeast colonization was seen in the maternal population. These results are consistent with the NeoCHG trial,^24^ which found no clear added benefit of 2% CHG over 1% CHG in hospitalized neonates with low or very low birth weight.

Colony counts with OHP were higher than with 1% CHG in the maternal population, consistent across organism groups. In the neonatal population, higher counts with OHP were observed only for Group B *Streptococcus*, with no evidence of differences for other outcomes. Total bacterial load was also higher in neonates born to mothers allocated to OHP. To our knowledge, this is the first in vivo comparison of OHP and CHG in these populations, and results suggest that OHP may be less effective for reducing colonization. The skin scores in the mothers treated with OHP were modestly worse than with 1% CHG, although the clinical significance of this is unclear.

In the neonatal population, the benefits of multiple applications increased over time, likely reflecting the rapid colonization or recolonization of hospitalized neonates.^28^ This pattern is consistent with the NeoCHG trial, which found a numeric though nonsignificant decrease in CFU with higher application frequency.^24^ In mothers, we observed no robust evidence favoring multiple applications, suggesting a single cleanse may be sufficient, although reduced swab sample availability at later time points limits certainty. Time points were selected pragmatically to reflect feasibility in each population, and results may therefore reflect colonization risk over the admission period rather than the instantaneous effect of antiseptic application. Later time point sampling decreased because of delivery and discharge, supporting a single maternal cleanse and short neonatal follow-up (or postdischarge sampling). Extended follow-up could target neonates expected to stay longer while generalizing to infants with shorter stays.

There was no evidence that SAE frequency differed between the trial arms in either population and no signal of postantiseptic neonatal hypothermia, supporting the safety of 1% CHG for future trials. These findings align with NeoCHG, which reported no safety concerns.^24^

Most research on intrapartum antiseptic use has focused on vaginal cleansing before cesarean delivery, which in meta-analyses was found to reduce maternal infection^29^ and is recommended by the WHO. However, routine cleansing before vaginal births is not recommended due to insufficient evidence.^12^ This question remains critical in LICs, where infectious disease burden is high and gram-negative organisms predominate.^30,31^ This study demonstrated that vaginal cleansing during labor lowered bacterial density, which could reduce maternal infection risk; a randomized clinical trial powered for clinical end points is warranted.

Lowering bacterial density in the vaginal tract may reduce neonatal pathogen exposure and vertical transmission. A South African trial published in 2009^9^ found no effect of CHG vaginal and neonatal cleansing on EOS, but gram-negative neonatal infections have increased substantially since,^32^ suggesting potential benefit today. Although CHG vaginal cleansing appears safe for neonates in this and other studies,^27^ potential risks to neonates, including altered skin microbiome, should be balanced against infection risk.

Antiseptic cleansing of neonates may reduce bloodstream infections, although causal pathways are less clear than in mothers, because neonates also acquire pathogens from the hospital environment,^33^ with *Klebsiella* species a particular issue in LICs. CHG may therefore be most effective within a multimodal infection-prevention strategy targeting health care–associated sepsis.^28,34^ In community-born neonates, CHG application reduced infection risk in low- and middle-income countries and lowered mortality in neonates with low birth weight after a single application.^35^ Hospital-based evidence also suggests a benefit: a Zambian study reported fewer culture-positive bloodstream infections with CHG cleansing but no difference in culture-negative sepsis or mortality.^36^ An Indian trial found a reduction (though not statistically significant) in Gram-positive bloodstream infections with CHG cleansing,^37^ and weekly CHG cleansing as part of a bundle was associated with reduced hospital mortality in Zambia.^38^ CHG cleansing is widely used in neonatal units worldwide, and a large pragmatic trial evaluating its safety, effectiveness, pharmacokinetics, and microbiome effects in high-risk neonates is warranted.

### Strengths and Limitations

Key strengths of this study include recruiting neonates within 24 hours of birth, allowing evaluation of antiseptic use during early colonization, and the factorial design, which enabled efficient comparison of antiseptic type, concentration, and application frequency within 1 study. Inclusion of both maternal and neonatal populations yielded complementary insights into colonization dynamics in the birth canal and on neonatal skin.

Limitations include the single-center setting, which may limit generalizability, and the use of bacterial load—including both pathogenic and nonpathogenic organisms—as a surrogate outcome that may not directly translate to clinical end points. Fewer samples at late time points, due to mothers delivering and neonates being discharged, reduced precision for evaluating application frequency. In addition, the skin-score tool may have limited sensitivity for detecting milder reactions.^39^

## Conclusions

This randomized clinical trial, to our knowledge the first comparative trial of OHP and CHG in laboring women and neonates, found that a single application of 1% CHG reduced acquisition of potentially pathogenic bacteria in both the vaginal canal and on neonatal skin. No safety concerns were identified. These findings support 1% CHG as the lead regimen for evaluation in a larger multifactorial infection-prevention trial powered on clinical outcomes.

## Notes

Supplement 1.
Trial Protocol

Supplement 2. **eAppendix.** Protocol Amendments **eMethods 1.** Skin Score Assessment Criteria **eMethods 2.** Six-Swab Technique for Maternal Antiseptic Application **eMethods 3.** Microbiological Sampling and Laboratory Procedures **eMethods 4.** Adverse Event Grading Scales (Maternal) **eMethods 5.** Adverse Event Grading Scales (Neonatal) **eMethods 6.** Priors for Bayesian ACCEPT Analyses **eFigure 1.** Maternal Bacterial-Load Trajectories **eFigure 2.** Neonatal Bacterial-Load Trajectories **eTable 1.** Additional Baseline Variables (Maternal) **eTable 2.** Additional Baseline Variables (Neonatal) **eTable 3.** Intervention Delivery (Maternal) **eTable 4.** Intervention Delivery (Neonatal) **eTable 5.** Primary and Secondary Outcomes (Maternal) **eTable 6.** Primary and Secondary Outcomes (Neonatal) **eTable 7.** Main Effects Interaction Analyses (Maternal) **eTable 8.** Main Effects Interaction Analyses (Neonatal) **eTable 9.** Subgroup Analyses (Maternal) **eTable 10.** Subgroup Analyses (Neonatal) **eTable 11.** ACCEPT Posterior Probability Analyses (Maternal) **eTable 12.** ACCEPT Posterior Probability Analyses (Neonatal) **eTable 13.** Sensitivity Analyses (Maternal) **eTable 14.** Sensitivity Analyses (Neonatal) **eTable 15.** SAE Summary Tables (Maternal) **eTable 16.** SAE Summary Tables (Neonatal) **eTable 17.** SAE Line Listing (Maternal) **eTable 18.** SAE Line Listing (Neonatal)
eReferences

Supplement 3.
Data Sharing Statement
