# Supplementary material for: Antiseptic Cleansing to Reduce Vertical Transmission of Pathogens to Neonates: The NeoVT-AMR Randomized Clinical Trial
Source: JAMA Netw Open. 2026 Jun 11;9(6):e2615665. doi: 10.1001/jamanetworkopen.2026.15665 (PMC13261496; doi:10.1001/jamanetworkopen.2026.15665)
Supplement: Supplement 1. — Trial Protocol [file jamanetwopen-e2615665-s001.pdf]

DocuSign Envelope ID: B928C439-2A10-4CA0-932F-B711DAAFBA4E

St George's University of London
University of Malawi College of Medicine
LSTM Liverpool School of Tropical Medicine
University of Liverpool
MRC Clinical Trials Unit
UCL

Strategies to reduce vertical transmission of multi-drug resistant pathogens to neonates (NeoVT-AMR)

PROTOCOL VERSION: v**6.0**
Date: 09-Feb-2023

Authorised by:

Name: Professor Mike Sharland
Role: Chief Investigator
Signature: DocuSigned by: Mike Sharland 77159C24682D45E...

Date: 2023-Mar-28

Name: Michelle Clements
Role: Trial Statistician
Signature: DocuSigned by: Michelle Clements AD78954D2C79466...

Date: 2023-Mar-27

Funder

DocuSign Envelope ID: B928C439-2A10-4CA0-932F-B711DAAFBA4E

The NeoVT-AMR project is funded by the Medical Research Council Seed funding Call - Global Maternal and Neonatal Health 2019. Grant reference number: MR/T039035/1.

INVESTIGATORS

Principle Investigator: Professor David Lissauer MBChB BMedSci MRCOG
Organisation: University of Liverpool Institute of Translational Medicine
Tel: +265992892149 Email: david.lissauer@liverpool.ac.uk

Chief Investigator: Professor Mike Sharland MBBS BSc MRCP MD DTM&H FRCPCH
Organisation: St George's, University of London; Institute of Infection & Immunity
Tel: +44 (0)208 725 5382 Email: msharland@sgul.ac.uk

Co-Investigator: Dr Aisleen Bennett MBBS MRes DTM&H MRCPCH
Organisation: St George's, University of London; Institute of Infection & Immunity
Tel: +44 (0)208 725 5382 Email: abennett@sgul.ac.uk

Co-Investigator: Dr Emily Beales MBChB BMedSc
Organisation: St George's, University of London; Institute of Infection & Immunity
Tel: +44 (0)208 725 5382 Email: ebeales@sgul.ac.uk

Co-Investigator: Dr Louise F Hill BMedSc, MBChB, MRCPCH, AFHEA
Organisation: St George's, University of London; Institute of Infection & Immunity
Tel: +44 (0)208 725 5382 Email: lhill@sgul.ac.uk

Co-Investigator: Dr Julia Bielicki BA MB/BChir MaA DPMSA MRCPH FMH Kinder MPH FMH Infektiologie
Organisation: St George's, University of London; Institute of Infection & Immunity
Tel: +44 (0)208 725 5382 Email: jbielick@sgul.ac.uk

Co-Investigator: Dr Luis Gadama MBBS MMED O&G FRCOG
Organisation: Kamuzu University of Health Sciences
Email: lgadama@medcol.mw

Co-Investigator: Dr Maranatha Kasonde Banda MBBS and MMed
Organisation: Kamuzu University of Health Sciences
Email: mkasonde17@gmail.com

Co-Investigator: Bertha Maseko
Organisation: Malawi Liverpool Wellcome Trust Department Clinical Research Programme
Email: bmaseko@mlw.mw

Co-Investigator: Dr Michelle Clements PhD MSci
Organisation: University College London Department: MRC Clinical Trials Unit
Tel: +44 (0)20 7670 4912 Email: michelle.clements@ucl.ac.uk

Co-Investigator: Professor Ann Walker FMedSci
Organisation: University College London Department: MRC Clinical Trials Unit
Tel: +77 (0)20 7670 4912 Email: rmjlasw@ucl.ac.uk

DocuSign Envelope ID: B928C439-2A10-4CA0-932F-B711DAAFBA4E

Co-Investigator: Professor Nicholas Feasey MBBS MSc BSc MRCP DTM&H FRCPath
Organisation: Liverpool School of Tropical Medicine Clinical Sciences
Email: nicholas.feasey@lstmed.ac.uk

Co-Investigator: Dr Samantha Lissauer MBChB MRCPCH PhD
Organisation: University of Liverpool
Tel: +265992892149 Email: slissauer@mlw.mw

Co-Investigator: Dr Kondwani Kawaza MBBS (MW), FCPaeds (SA), Cert Neon (SA)
Organisation: Kamuzu University of Health Sciences
Tel: +265 9 922 17282 Email: kkawaza@medcol.mw

TRIAL SPONSOR AND GRANT HOLDER

St George's, University of London (SGUL)
Paediatric Infectious Diseases Research Group
Institute for Infection and Immunity
2nd Floor, Jenner Wing,
Cramner Terrace

London. SW17 0RE
United Kingdom

Switchboard: +44 (0)208 725 0666/5382
Fax: +44 (0)208 8725 0716

Operations Manager: Dr Tatiana Munera Huertas PhD
Organisation: St George's, University of London; Institute of Infection & Immunity
Tel: +44 (0)208 725 5382 Email: tmunerah@sgul.ac.uk

Project Manager: Yasmine Yau
Organisation: St George's, University of London; Institute of Infection & Immunity
Tel: +44 (0)208 725 5382 Email: yyau@sgul.ac.uk

Project Co-ordinator: Caroline Albrecht
Organisation: St George's, University of London; Institute of Infection & Immunity
Tel: +44 (0)208 725 5382 Email: calbrech@sgul.ac.uk

COORDINATING CENTRE /TRIAL ADMINISTRATION

Please direct all queries to the Project Management Team at SGUL in the first instance; clinical queries will be passed to the Chief Investigator at SGUL via the team as appropriate.

Malawi-Liverpool-Wellcome Trust Clinical Research Programme. Tel: +265 1811628 / 18122429
Fax: +265 1875774
Queen Elizabeth Central Hospital
College of Medicine
P.O. Box 30096
Chichiri, Blantyre 3
Malawi.

RECRUITING SITE

Zomba Central Hospital
P.O.Box 21,

Tel: +265 1 526 266/525 195

DocuSign Envelope ID: B928C439-2A10-4CA0-932F-B711DAAFBA4E

Zomba, MALAWI

TABLE OF CONTENTS

ABBREVIATIONS **7**

EXECUTIVE SUMMARY **8**

1. BACKGROUND INFORMATION **11**

2. RATIONALE OF THE RESEARCH PROJECT **12**

2.1. RESEARCH QUESTIONS 13

3. AIMS AND OBJECTIVES **13**

4. METHODOLOGY **13**

4.1. TYPE OF RESEARCH STUDY 13
4.2. STUDY PLACE 13
4.3. STUDY POPULATION 13
4.4. STUDY PERIOD 13
4.5. SAMPLE SIZE 14
4.5.1. SELECTION OF PARTICIPANTS 14
4.5.1.1. PATIENT INCLUSION CRITERIA 14
4.5.1.2. PATIENT EXCLUSION CRITERIA 15
4.5.2. CO-ENROLMENT GUIDELINES 15
4.5.3. INFORMED CONSENT AND WITHDRAWAL OF CONSENT 15
4.5.4. PRE-RANDOMISATION PROCEDURES 16
4.5.5. RANDOMISATION 17
4.5.5.1. RANDOMISATION PRACTICALITIES 17
4.5.5.2. RANDOMISATION CODES AND UNBLINDING 17
4.5.5.3. CO-ENROLMENT GUIDELINES AND REPORTING 17
4.6. TREATMENT OF PATIENTS 17
4.6.1. INTRODUCTION 17
4.6.2. TRIAL TREATMENTS 18
4.6.2.1. JUSTIFICATION FOR CHOICE OF INTERVENTION 18
4.6.2.1.1. MATERNAL STRATA 18
4.6.2.1.2. NEONATAL STRATA 19
4.6.2.2. MATERNAL TREATMENT 19
4.6.2.3. NEONATAL TREATMENT 20
4.6.2.4. DISPENSING 20
4.6.2.5. DOSE INTERRUPTIONS AND DISCONTINUATIONS 20
4.6.2.6. STOPPING CHG/OCT EARLY (PERMANENT DISCONTINUATION) 21
4.6.2.7. HANDLING CASES OF OVERDOSE 21
4.6.2.8. ACCOUNTABILITY AND UNUSED PRODUCTS 21
4.6.3. CONTROL GROUP 21
4.6.3.1. COMPLIANCE AND ADHERENCE 22
4.6.4. UNBLINDING/UNMASKING 22
4.6.5. PROTOCOL TREATMENT DISCONTINUATION 22
4.6.6. COMPLIANCE AND ADHERENCE 22
4.6.7. NON-TRIAL TREATMENT 22
4.6.7.1. MEDICATIONS PERMITTED 22
4.6.7.2. MEDICATIONS NOT PERMITTED 22
4.6.7.3. MEDICATIONS TO BE USED WITH CAUTION 22
4.7. ASSESSMENTS AND FOLLOW-UP 23
4.7.1. TRIAL ASSESSMENT SCHEDULE 23
4.7.1.1. MATERNAL STRATA 23
4.7.1.2. NEONATAL STRATA 25
4.7.2. MICROBIOLOGY PROCEDURES FOR ASSESSING EFFICACY 26
4.7.3. PROCEDURES FOR ASSESSING SAFETY 28
4.7.4. EARLY STOPPING OF FOLLOW-UP 28
4.7.5. PATIENT TRANSFERS 29
4.7.6. FOLLOW-UP 29
4.8. DATA COLLECTION AND GENERATION 29

DocuSign Envelope ID: B928C439-2A10-4CA0-932F-B711DAAFBA4E

4.8.1. METHODOLOGIES FOR DATA COLLECTION/GENERATION 29
4.8.2. DATA QUALITY AND STANDARDS 29
4.9. DATA MANAGEMENT AND QUALITY ASSURANCE 30
4.9.1. CENTRAL MONITORING 30
4.9.2. ON SITE MONITORING 30
4.9.3. DIRECT ACCESS TO PATIENT RECORDS 30
4.9.4. DATA MANAGEMENT, DOCUMENTATION AND CURATION 30
4.9.5. DATA SECURITY AND CONFIDENTIALITY 31
4.9.5.1. FORMAL INFORMATION/DATA SECURITY STANDARDS 31
4.9.5.2. MAIN RISKS TO DATA SECURITY 31
4.9.5.3. CONFIDENTIALITY 31
4.9.6. DATA SHARING AND ACCESS 31
4.9.6.1. SUITABILITY FOR SHARING 31
4.9.6.2. DISCOVERY BY POTENTIAL USERS OF THE RESEARCH DATA 31
4.9.7. DATA GOVERNANCE OF ACCESS 31
4.9.8. RESTRICTIONS OR DELAYS TO SHARING, WITH PLANNED ACTIONS TO LIMIT SUCH RESTRICTIONS 32
4.10. STATISTICAL CONSIDERATIONS 32
4.10.1. METHOD OF RANDOMISATION 32
4.10.2. OUTCOME MEASURES 32
4.10.2.1. CO-PRIMARY OUTCOME MEASURES 32
4.10.2.2. SECONDARY OUTCOME MEASURES 32
4.10.3. INTERIM MONITORING AND ANALYSES 32
4.10.4. ANALYSIS PLAN (BRIEF) 33
4.11. RESULTS PRESENTATION AND DISSEMINATION OF FINDINGS 33

5. SAFETY REPORTING 35

5.1. DEFINITIONS 35
5.1.1. PRODUCT 36
5.1.2. ADVERSE EVENTS 36
5.1.3. SAFETY MANAGEMENT 37
5.1.4. EXEMPTED ADVERSE EVENTS 37
5.2. INVESTIGATOR RESPONSIBILITIES 37
5.3. INVESTIGATOR ASSESSMENT 37
5.3.1. SERIOUSNESS 37
5.3.2. SEVERITY OR GRADING OF ADVERSE EVENTS 37
5.3.3. CAUSALITY 37
5.3.4. NOTIFICATION 38
5.4. SGUL (SPONSOR) RESPONSIBILITIES 39

6. ETHICAL CONSIDERATIONS 40

6.1. COMPLIANCE 40
6.1.1. REGULATORY COMPLIANCE 40
6.1.2. SITE COMPLIANCE 40
6.2. ETHICAL CONDUCT 40
6.2.1. ETHICAL CONSIDERATIONS .... **ERROR! BOOKMARK NOT DEFINED.**
6.2.2. ETHICAL APPROVALS 41
6.3. TRIAL CLOSURE 41

7. INDEMNITY 41

8. OVERSIGHT AND TRIAL COMMITTEES 42

8.1. TRIAL MANAGEMENT GROUP (TMG) 42
8.2. INDEPENDENT DATA MONITORING COMMITTEE (DMC) 42
8.3. ROLE OF THE TRIAL SPONSOR 42

9. PATIENT AND PUBLIC INVOLVEMENT 42

9.1. POSSIBLE CONSTRAINTS 42

10. FUNDING AND JUSTIFICATION 44

11. REFERENCES 46

Abbreviations

ABBREVIATION	EXPANSION
AE	Adverse event
AMR	Antimicrobial resistance
AR	Adverse reaction
CF	Consent Form
CFU	Colony forming units
CI	Chief Investigator
CHG	Chlorhexidine
COMREC	College of Medicine Research Ethics Committee
CRF	Case Report Form
D	Day
DM	Data Manager
DMC	Independent Data Monitoring Committee
DMP	Data Management Plan
EU	European Union
GCP	Good Clinical Practice
HAI	Healthcare Associated Infection
IMPAACT	International Maternal Pediatric Adolescent AIDS Clinical Trials Network
IMP	Investigational medicinal product
IPC	Infection prevention and control
ISRCTN	International Standard Randomised Controlled Trial Number
ITT	Intention-to-treat
LMIC	Low or middle income country
MHRA	Medicines and Healthcare products Regulatory Agency
MLW	Malawi Liverpool Wellcome Trust
MOP	Manual of Operations
MRC	Medical Research Council
NEC	Necrotising enterocolitis
NIMP	Non-investigational-medicinal product
OCT	Octenidine
OHP	Octenidine 0.1% Phenoxyethanol 2% (trade name Octenisept Ò)
PI	Principal Investigator
PIL	Participant Information Leaflet
PMRA	Pharmacy and Medicines Regulatory Authority
RCT	Randomised controlled trial
REC	Research Ethics Committee
SAE	Serious adverse event
SAP	Statistical Analysis Plan
SAR	Serious adverse reaction
SD	Standard deviation
SGUL	St George’s University of London
SOP	Standard operating procedure

SmPC	Summary of Product Characteristics
SSA	Site-specific approval
SUSAR	Suspected unexpected serious adverse reaction
TMF	Trial Master File
TMG	Trial Management Group
UAR	Unexpected adverse reaction
VT	Vertical transmission
WHO	World Health Organization

Executive Summary

Type of research study

This is a factorial randomised controlled pilot trial.

The problem to be studied

Neonatal infection is a leading cause of morbidity and mortality in low income countries, and rates of antimicrobial resistance in causative organisms are rising rapidly. Strategies to reduce the incidence of neonatal sepsis are urgently required. Early onset neonatal infection (EOS) often results from transmission of pathogenic organisms from labouring mothers to their infants. Application of antiseptic to the genital tract of labouring women and neonates is potentially a cheap, practical, scale-able intervention to reduce transmission of Multi Drug Resistant organisms from mother to child and to reduce EOS, however equipoise exists regarding the effectiveness of such an intervention.

The objectives

Primary outcome measures:

Mothers: change in vaginal and perineal bacterial load (in colony forming units)

Neonates: change in neonatal skin bacterial load (in colony forming units)

Secondary outcome measures:

Mothers:

- Maternal toxicity – score and grade (Tolerability and safety)
- Change in neonatal skin bacterial load in neonates exposed to maternal antiseptic, compared to control
- Serious adverse events

Neonates:

- Adapted neonatal skin condition score (safety) (absolute score and grade)
- Temperature (change in absolute temperature and grade (hypothermia))
- Serious adverse events

Methodology

Setting:

Hospital labour ward, postnatal ward and neonatal unit in Zomba Central Hospital in Malawi.

Type of participants to be studied:

Women presenting in labour at any gestation with or without rupture of membranes.

Neonates born at a healthcare facility within the last 24 hours, weighing >1000g and not born to enrolled women.

Interventions to be compared:

DocuSign Envelope ID: B928C439-2A10-4CA0-932F-B711DAAFBA4E

Antiseptic (3 levels): Chlorhexidine (CHG) concentration 1% vs 2% vs 0.1% Octenidine and 2% 2-phenoxyethanol) (OHP) application to vagina and perineum in labouring women or whole body in neonates.

Frequency (2 levels): single application vs multiple application [4 hourly (in mothers) or 24 hourly (in neonates)].

Compared to control groups who will receive normal standard of care.

Randomisation:

Mothers and neonates are each allocated 1:1:1:1:1:1:1 into 6 intervention arms and 1 control arm. Randomisation is by permuted blocks to guard against bias introduced over time, such as outbreaks of pathogenic bacteria in the hospital.

Both strata:

1% CHG single application

1% CHG multiple application (mothers: 4hrly; neonates: 24hrly)

2% CHG single application

2% CHG multiple application (mothers: 4hrly; neonates: 24hrly)

0.1% OHP single application

0.1% OHP multiple application (mothers: 4hrly; neonates: 24hrly)

Control group

Number of participants:

Within each group of mothers and neonates, 147 individuals will be recruited – 21 per treatment combination or control.

In addition, babies born to participating mothers will have swabs taken and are included in the total participant count. The total sample size will be 441 individuals.

Duration/planned follow-up:

16 months to complete recruitment and follow-up. Final follow up for both groups will be 28 days after enrolment.

Expected findings

Trial hypothesis: Topical antiseptic use leads to greater reductions in bacterial colonisation in both mothers and neonates compared to control. Reduction is greater at higher frequency of application and at higher concentration of CHG.

1. Background Information

Infection remains a major cause of neonatal mortality, with serious bacterial infection (SBI) responsible for an estimated 680,000 neonatal deaths per year in low income countries (LIC)(1). Gram negative pathogens, such as *Escherichia coli*, and *Klebsiella* spp., are now the most common cause of SBI in many low and middle income countries (LMIC)(2,3). A rise in antimicrobial resistance (AMR) of these pathogens has recently been observed, and it is estimated that resistant organisms are responsible for 30% of all neonatal infectious deaths globally(4). Extended Spectrum Beta-Lactamase producing Enterobacteriaceae (ESBL-E)(5,6) are of particular concern in the African context as access to carbapenems is limited.

The majority of SBI identified in neonates in LICs appears to be early onset sepsis (EOS), occurring within the first 72 hours of life(5,7). A major mechanism of such EOS is through pathogens acquired during birth from the maternal perineum and genital tract – so called vertical transmission (VT) from mother to child(8–11). There is a high frequency of carriage of ESBL-E in heathy individuals in LMIC, ranging from 17- 22% in Africa(12,13) to 46% in the West Pacific region, with a trend towards increasing prevalence over time(13). These data highlight the potential for interventions to reduce vertical transmission of ESBL-E pathogens in neonatal sepsis.

New strategies to reduce SBI in neonates are urgently required. In high-income countries (HIC) intra-partum antibiotics have been used to reduce the risk of mother to child transmission of Group B *Streptococcus* (GBS), however both the logistics of widespread administration in LICs and the diversity of pathogens coupled with the rise in AMR necessitate an alternative solution for LMIC. Antiseptics - which can be used vaginally or applied topically to neonates - are a potential method to reduce VT of resistant pathogens as they are cheap, widely used, generally well tolerated, and easy to administer, even in resource poor settings.

The most widely used antiseptics in maternal and neonatal populations are topical chlorhexidine (CHG), octenidine (OCT) and octenidine 0.1% combined with phenoxyethanol 2% (OHP) (trade name Octenisept®). CHG is routinely used for skin cleaning prior to procedures in neonatal units and has been shown to significantly reduce skin bacterial load for up to 72 hours following application(14,15). OHP is used widely as a treatment for bacterial vaginosis and is used as the routine antiseptic in many neonatal units in Europe(16). Importantly some reports have shown higher activity of OCT against gram negative organisms compared to CHG(17–20).

A large randomised controlled trial (n=8011) of vaginal and neonatal CHG wipes for the prevention of early-onset GBS disease conducted in South Africa from 2004-2006 found no evidence of an effect on culture positive or clinically diagnosed EOS(21). However, this study was conducted 15 years ago when the epidemiology of EOS differed substantially from that observed today and focused on GBS rather than gram negative/drug resistant isolates. In addition, this study took place in a setting with a relatively low incidence of culture positive EOS (3/1000 recruited births), involved a large study team which significantly improved baseline standards of care independent of the intervention, used low concentration of CHG (0.5%), and the majority of labouring women in the intervention arm only received one CHG application(21,22). Large, non-randomised studies of CHG vaginal administration conducted in LIC prior to this trial had reported substantial reductions in infection related mortality in infants(23,24) and a systematic review concluded that intrapartum, vaginal chlorhexidine may reduce neonatal

infection/sepsis but the impact of concentration on outcome was unknown(25). A recent Cochrane review highlighted the lack of evidence for the use of CHG to prevent non-GBS EOS in neonates(26).

Large pragmatic trials are required to investigate whether antiseptic use in neonates and labouring women reduces clinical sepsis in moderate-high incidence settings, particularly with highly resistant organisms. However, equipoise exists surrounding the optimal choice of drug, dosing and scheduling of antiseptic administration and whether maternal or neonatal administration is more effective. This pilot trial aims to investigate the effect of different strategies of topical antiseptic use in labouring women and new-born infants on reducing bacterial load in the female genital tract and on the skin of new-borns. Results from this trial will inform the design of a planned pragmatic clinical trial of antiseptic use to prevent EOS in neonates in LMIC. A complementary trial investigating the optimal antiseptic regime to reduce skin bacterial load in low birth weight (LBW) infants admitted to neonatal intensive care units (NICUs) - designed to inform antiseptic interventions to reduce late onset sepsis and healthcare associated transmission of pathogens in NICUs in Africa and Asia - has been funded as a pilot by the Medical Research Council (MRC – JGHT call, Chief Investigator Prof Sharland).

2. Rationale of the Research Project

This trial will identify the optimal antiseptic strategy for labouring women and neonates to be tested in a larger clinical trial of interventions to prevent neonatal sepsis. It is part of a programme of work to identify pragmatic, scale-able interventions that can be used in clinical practice to protect neonates in low income countries from serious infection. Neonatal sepsis has long-term consequences for affected infants, their families and the health care system, with a significant social and economic impact on communities in low income countries. Prevention of neonatal sepsis is a therefore a global health priority, reflected in the sustainable development goal (SDG) 3.2 to reduce neonatal mortality to as low as 12/1000 live births(27). This trial is part of a global effort to address the escalating challenge of antimicrobial resistance (AMR). It will provide data to inform antiseptic use to reduce transmission of pathogenic bacteria from mothers to infants, including resistant gram-negative bacteria which are emerging as a major global cause of serious neonatal infections. Recent child mortality surveillance data from sub-Saharan Africa and Asia identified gram negative bacteria as the most common contributory pathogens in neonatal deaths(28).

Potential long-term beneficiaries of this research include neonates and their families, health care workers, and health systems. Results from this trial, and from research leading on from it, will inform public health and infection prevention and control (IPC) strategy at local, national and international levels. This is a clinical trial which will be conducted in the maternity, postnatal and neonatal units of a busy government hospital in a low-income country. It will provide opportunities for education and training on IPC for health care providers and for the families of new-born infants. Follow-up qualitative assessments of the acceptability and feasibility of the interventions will be an integral part of the trial. These will be shared with local clinical teams to support the development of successful, locally appropriate, IPC guidelines.

This pilot trial is one step in a programme of work designed to reduce the burden of neonatal sepsis. A clinical trial is planned to follow on from this trial, and the trial will need to be completed and the results disseminated, before any positive findings could be incorporated into policy.

2.1. Research questions

What is the optimal strategy in terms of efficacy and safety of using a topical antiseptic applied to i) the genital tract in labouring women and ii) the skin in newborn infants, to reduce bacterial load?

3. Aims and objectives

The broad objective is to assess the optimal regime of choice of antiseptic agent and frequency of application to reduce bacterial colonization in the genital tract of laboring women and skin of newborns. The specific objectives of this pilot trial are:

To assess the efficacy of different antiseptic agents and regimes, in reducing bacterial load when applied to the vagina and perineum of labouring women and the skin of neonates.

To evaluate the safety of administering topical antiseptics to labouring women and to neonates.

4. Methodology

4.1. Type of Research Study

The experimental approach is a randomised controlled trial. The trial design incorporates two independent strata: labouring women (mothers) and neonates (born to mothers who have not been recruited into the trial). The trial design for each group is a 3x2 factorial plus control arm, which enables efficient comparison of the choice of antiseptic (drug), the concentration (dose) and frequency of application (duration).

4.2. Study Place

The research setting is in the labour ward, and the neonatal and postnatal ward in Zomba Central Hospital, Zomba, Malawi.

4.3. Study Population

The study will recruit women presenting in labour at any gestation with or without rupture of membranes and neonates born in a healthcare facility within the last 24 hours, weighing >1000g and not born to enrolled women.

4.4. Study Period

This study will take 16 months to complete recruitment and follow-up. Final follow up for both groups will be 28 days after enrolment. For full study timelines see below.

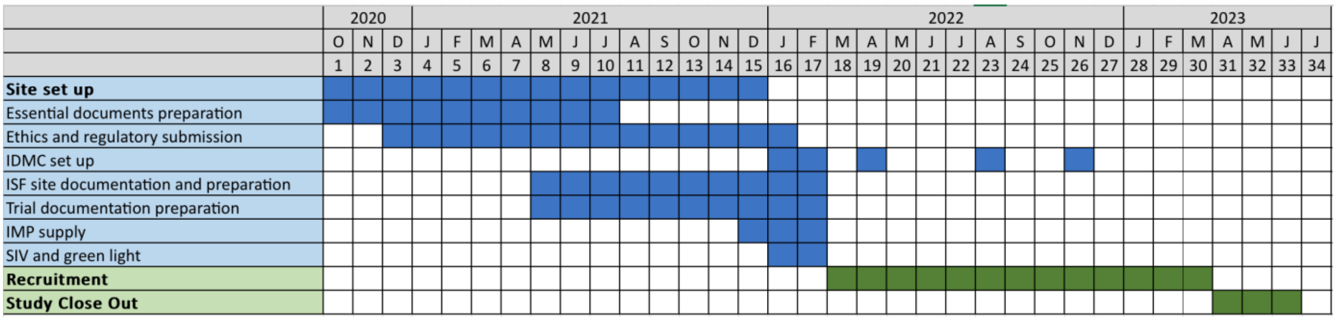

4.5. Sample Size

The two independent strata each have a factorial design, with an additional control group. Within each stratum, 147 individuals will be recruited – 21 per treatment combination or control. Additionally, 147 babies from mothers in the maternal stratum will also be swabbed at birth.

Recruitment feasibility has been taken into account during the writing of this protocol. Recent audit data (August 2020 – January 2021) in Zomba Central Hospital showed that there were an average of 138 births a week, which is equal to an average of 19 births per day. Rates of caesarean section ranged from 19% to 52%. This indicates that recruitment will be feasible in around 12 months.

The primary endpoints of log colony forming units (CFUs) are widely accepted to be normally distributed. Although all-cause mortality is more relevant, and will be used in the planned future trial, it is not feasible for this pilot due the increased sample size requirements with a binary outcome. High bacterial load (reflected by log CFUs) is associated with increased risk of neonatal sepsis and consequently provides a good balance between relevance to the scientific question and power in a pilot trial.

However, there is very little data on bacterial load in neonates in LMICs. Additionally, bacterial loads are expected to differ across locations complicating interpretation of previous studies. For example, previous studies in neonates have found varying effects of CHG on log colony counts: a decrease from baseline to 24h of 0.2 SD with 1% CHG in Nepal(29) and 2SDs with 2% CHG in the USA(30). Sample size calculations used standard formula with Bonferroni adjustment for multiple testing within arms of frequency and antiseptic. Within each stratum, 147 individuals randomized to 7 treatment groups, 21 participants per group, provides 90% power to detect a difference of 0.82 standard deviations (SDs) between antiseptics (two-sided α=0.012) and 0.58 SDs between frequencies (α=0.05) (80% power for 0.72 and 0.50 SDs, respectively). This also provides 90% power to detect a difference of 1.01 SD between each antiseptic and control (α=0.012), and 0.90 SD between frequency and control (α=0.025) (0.88 and 0.79 SDs at 80% power). These expected effects were judged by clinical and statistical members of the team to be sufficient for selecting a treatment regimen for a future trial.

4.5.1. Selection of participants

There will be no exceptions to eligibility requirements at the time of randomisation. Questions about eligibility criteria must be addressed to the trial management group prior to attempting to randomise the participant.

The eligibility criteria are the standards used to ensure that only medically appropriate mothers and neonates are considered for this trial. Mothers or neonates not meeting the criteria should not join the trial. For the safety of the participants, as well as to ensure that the results of this trial can be useful for making treatment decisions regarding other mothers/neonates in similar situations, it is important that no exceptions be made to these criteria for admission to the trial.

Those enrolled in the trial will be those who have been admitted to hospital. Mothers or neonates will be considered eligible for enrolment in this trial if they fulfil all the inclusion criteria and none of the exclusion criteria as defined below.

4.5.1.1. Patient inclusion criteria

Mothers:

1. Presenting in labour with or without rupture of membranes

DocuSign Envelope ID: B928C439-2A10-4CA0-932F-B711DAAFBA4E

Neonates:

1. Born in a healthcare facility
2. Postnatal age at randomisation <24 hours
3. Birth weight >1000g

4.5.1.2. Patient exclusion criteria

Mothers:

1. Under the age of 18 (minor in Malawi)
2. Any contra-indication to digital vaginal examination
3. In active labour (regular contractions and/or cervical dilatation of more than 4cm)
4. Poor perineal and vaginal skin condition as judged by clinician
5. Planned elective caesarean-section delivery
6. Known or suspected allergy to chlorhexidine or octenidine or phenoxyethanol
7. Intrauterine death confirmed or expected before randomsiation
8. Antiseptic application or enrolment in the trial determined inappropriate in the opinion of the enrolling clinician
9. Any recent or planned (within 4 hours) iodine application to the perineum or vagina
10. Unable to obtain consent

Neonates:

1. Born by planned elective caesarean section
2. Born to mothers recruited in the trial
3. Poor skin condition (skin score of 2 or more in any of three domains (see Appendix I)) at the time of enrolment
4. Known congenital or acquired skin disorder or defect at time of enrolment
5. Antiseptic application or enrolment in the trial determined inappropriate in the opinion of the enrolling clinician
6. Any recent or planned (within 4 hours) iodine application to body
7. Any planned or previous lumbar puncture
8. Unable to obtain parental or guardian consent

4.5.2. Co-enrolment guidelines

Co-enrolment in previous or future trials is considered in Section 4.5.5.3.

4.5.3. Informed consent and withdrawal of consent

Posters advertising the trial will be displayed in the antenatal ward, antenatal clinic and labour ward of Zomba Central Hospital. Where possible, information about the trial will be given antenatally. The poster contains the contact information of the coinvestigator and consultant obstetrician at Zomba Central Hospital for the trial team, for pregnant women or parents/carers who wish to obtain more information.

Maternal - Participant approach and consent:

Before the research midwife approaches a woman about the trial, she will check with the clinical team if there is any reason that this would be inappropriate. The clinical team will therefore act as gatekeepers for any participants, ensuring that they are both eligible and considered to be in a stable mental and physical condition before being approached, preventing unwanted disturbance.

Women who are already known to be in active labour will not be approached by the research midwife, and will not be eligible for inclusion.

Women who have been deemed appropriate for being approached will then be provided with a participant information leaflet, in a language they understand. The trial and the contents of the participant information leaflet will be verbally explained to them and they will be free to ask any questions. The women may take as much time as they need to discuss with their partners and consider joining the trial; but will be made aware that women in active labour will not be eligible for inclusion.

Newborns:
Parents/carers of newborns will be given information about the trial including a participant information leaflet in a language they understand. They will have the trial and contents of the participant information leaflet verbally explained to them and will be free to ask any questions about the trial. They may take as much time as they need to consider joining the trial, but will be made aware that only babies up to 24 hours old can be enrolled.

Both:
The doctor or midwife seeking consent, who will be fully trained in consent procedures, will confirm understanding of the important points of the trial.

It will be made completely and unambiguously clear that participation is voluntary and the labouring woman or parent/carer is free to refuse to participate in all or any aspect of the trial, at any time and for any reason, without incurring any penalty or affecting the treatment of the Mother or the baby.

Only following this formal written consent can be sought. Signed consent will be kept by the investigator and documented in both the labouring woman’s and the neonate’s notes and a copy given to the participant or neonate’s parent/carer. Consent may be given using a thumbprint with signed agreement from an independent witness.

If a participant or newborn’s parent/carer choses to discontinue trial treatment but remains willing to follow other trial procedures and follow-up schedule, the participant may remain in the trial “on-study, off-study treatment”. However, if they do not wish to remain on trial follow-up, their decision must be respected and the participant will be withdrawn from trial follow-up. The sponsor should be informed of this in writing using the appropriate documentation. Prior to transferring to routine follow-up, the participant or parent/carer will be asked whether they would be happy to have assessments performed as for the final study visit. They would be at liberty to refuse any or all individual components of the follow-up assessments and their wishes should be respected.

If follow-up is stopped early, the pseudonymised medical data collected during their participation in the trial will be kept and used in the analysis of the trial results; consent cannot be withdrawn for the use of historical pseudonymised data already collected.

4.5.4. Pre-randomisation procedures

Written informed consent to enter into the trial and be randomised will be obtained from Mothers (for their own participation) or parents/carers (for neonate participation) after explanation of the aims, methods, benefits and potential hazards of the trial and before any trial-specific procedures are performed.

For enrolment to the maternal stratum: mothers will be approached on admission to the labour ward, and when initial routine clinical procedures and communication have already been completed. For enrolment to the neonatal stratum: parents/carers will be approached after their baby has been admitted to the neonatal or post natal ward, and after initial routine clinical procedures and communication have been completed.

4.5.5. Randomisation

4.5.5.1. Randomisation practicalities

Randomisation will be performed using a random number generated as implemented in the statistical computing environment R. Randomisation will be by permuted blocks (random block sizes of 7 or 14 participants) to guard against bias introduced over time, such as outbreaks of pathogenic bacteria in the hospital. Participants will be randomised 1:1:1:1:1:1:1 to the 6 treatment and 1 control arms.

Randomisation will be built into electronic data systems using algorithms developed by the data management team at the Malawi Liverpool Wellcome Trust (MLW). The participant's trial number, treatment allocation and the date of randomisation will be entered into the trial register at the site.

4.5.5.2. Randomisation codes and unblinding

The allocation will not be blinded as this is not practical given the number of factors evaluated. However, the primary end point (total bacterial load in colony forming units (CFUs)) is quantitative in nature and will be assessed by laboratory staff blinded to randomisation. See Section 10.1.1 for further information.

4.5.5.3. Co-enrolment guidelines and reporting

Co-enrolment in another interventional trial is not permitted for the duration of follow-up. Co-enrolment in other non-interventional (i.e. observational) studies may be allowed but should be discussed with the trial management group before enrolment.

4.6. Treatment of patients

4.6.1. Introduction

All women and neonates will receive routine hospital care as laid out in local policies and guidelines for all procedures, except for the interventions of the trial including antiseptic application and the trial's clinical and microbiological assessments. Labouring women and neonates will be randomised into one of the 7 arms of the trial 1:1:1:1:1:1:1 as follows:

Figure 3: Trial arms

DocuSign Envelope ID: B928C439-2A10-4CA0-932F-B711DAAFBA4E

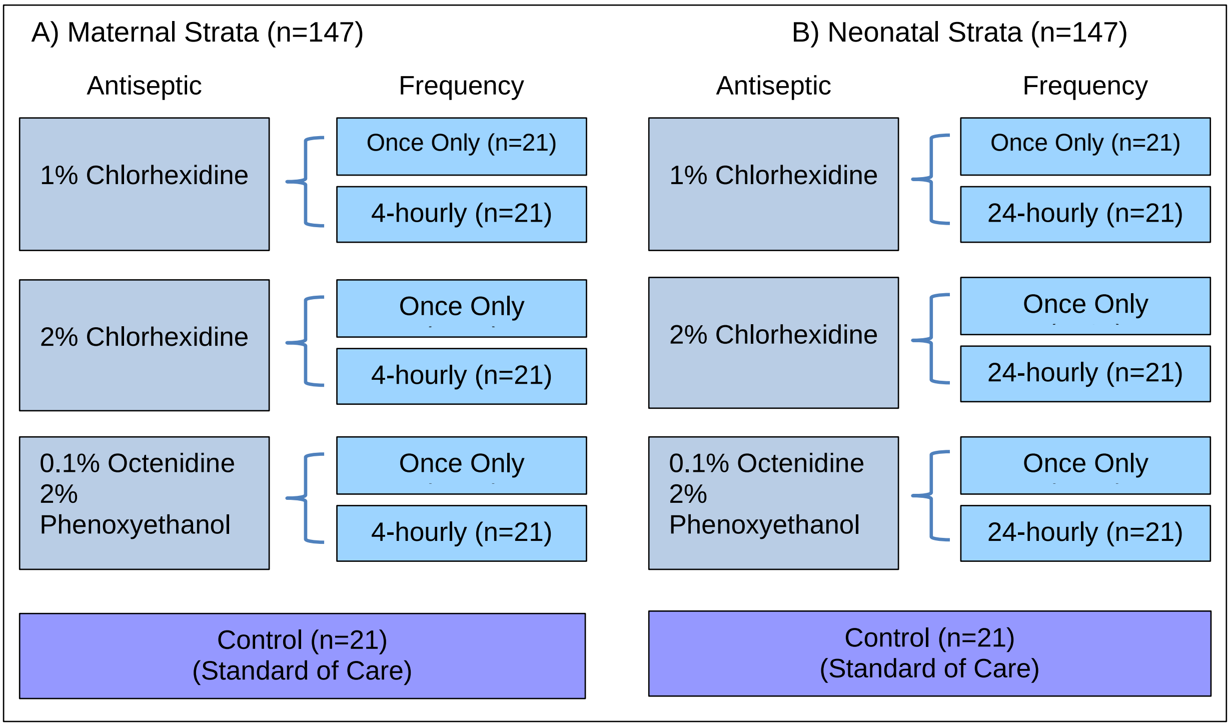

4.6.2. Trial treatments

At the point of recruitment baseline clinical and demographic data will be collected for all participants, and a baseline skin assessment performed and documented.

Participants in both strata, other than those randomised to the control group, will be randomised to either 1% CHG, 2% CHG or Octenidine dihydrochloride 0.1% phenoxyethanol 2% (OHP, trade name Octenisept®), at different frequencies (see above). Each application will be recorded in the appropriate CRF.

4.6.2.1. Justification for Choice of Intervention

4.6.2.1.1. Maternal strata

CHG is the most widely used antiseptic in labour and is on the WHO list of essential medicines(31). One percent CHG was well tolerated in labouring women from Zimbabwe(32,33), however, two

applications were required to achieve an ~80% reduction in bacterial load. This study will test CHG in concentrations up to 2% to determine if increasing concentration of CHG leads to increased efficacy. Concentrations will be limited to 2% because of a rare case report of mucosal damage with higher concentrations(34).

There is good data on safety and tolerability of OCT 0.1% (35,36) and OHP in female populations(18,37,38) and some evidence that OCT (and OHP) has improved action against gram negative organisms compared to CHG, particularly in the presence of organic material. We have elected to test the standard available preparation of 0.1% octenidine with 2% phenoxyethanol (OHP).

Antiseptic application in labour is invasive and requires personnel; interventions must therefore be limited to the minimum necessary to achieve adequate reductions in bacterial load. However, there is some evidence that more than one intervention may be required for optimum effect (see above). We therefore plan to compare a single vaginal cleansing intervention with 4 hourly repeat interventions up to a maximum of 6 applications, a frequency well tolerated by labouring women in Zimbabwe and consistent with WHO recommendations for frequency of vaginal examination during labour(33,39). A control arm receiving standard of care will also be recruited.

4.6.2.1.2. Neonatal strata

CHG is used in neonatal units (NNUs) in concentrations from 0.2-4%(40–43). Previous large- scale studies of CHG to decolonise neonatal skin have used concentrations towards the lower end of the range(22); however babies born in LMIC with high levels of environmental contamination may require relatively high concentrations to achieve effective decolonisation. This study will use an upper limit of 2% CHG because of rare reports of skin damage with concentrations of CHG ≥2%, although these are mostly limited to infants <1000g at birth(44). This trial is only recruiting neonates with a birth weight >1000g. OCT 0.1% and 0.1% Octenidine 2% Phenoxyethanol (OHP) are the standard antiseptics in neonatal unit’s in Germany and there are published data on safety in neonates(16), including premature newborns(45). This study of OHP in premature newborns also demonstrated that although phenoxyethanol undergoes cutaneous absorption, it almost completely metabolizes to 2-phenoxyacetic acid, which can be detected in the urine. As with the maternal strata, OHP will be tested at a single concentration because of limited published data.

Previous studies from high income countries have documented a significant decline in skin bacterial load for up to 48 hours following a single topical application of CHG(14), however infants from LMIC may be exposed to high levels of environmental contamination during birth and in the postnatal period. This study will therefore compare a single antiseptic application to 24 hourly repeat applications until discharge, up to a maximum of 72 hours. Twenty-four hourly applications were selected as the maximum feasible to scale up in a LMIC setting. A control arm receiving standard of care will also be recruited.

4.6.2.2. Maternal treatment

The antiseptic will be applied to the vagina and perineum. Participants will receive the intervention according to the frequency schedule they were assigned to (either single application or 4 hourly [during working hours, up to 6 applications]), aligned with routine maternal vaginal examinations.

Further details can be found in the MOP.

Before an antiseptic is applied or swabs are taken, a toxicity assessment will be performed (see Appendix II). This will assess for vaginal/vulval irritation, erythema, skin breakdown and swelling (more details in Section 4.7.3).

The normal bathing and washing regime will be permitted.

4.6.2.3. Neonatal treatment

The antiseptic will be applied topically to the whole body with the same volume of CHG/OHP, excluding the face to avoid the eyes.

Before an antiseptic is applied, the neonate's temperature will be measured and the skin condition score assessment will be performed. Whilst treatment is being applied, precautions should be taken to keep the baby warm and also to avoid pooling of the antiseptic around the baby. After the application, the neonate should be re-dressed and their body temperature will be measured after 15 minutes. The neonate should be observed for skin reactions at 30 minutes after each application.

In the case of rare and highly unlikely accidents such as splash injury to the eye from antiseptic, flush the affected area with copious saline as quickly as possible and inform the local paediatric services. See MOP for further details.

The normal bathing, washing and cord care regime delivered by the nursing staff or family will be permitted.

4.6.2.4. Dispensing

Antiseptic will be transported to the ward in pre-defined concentrations. Containers for individual use will be prepared on the ward and labelled for clinical trial use, adding the trial number to each container immediately after randomisation. These containers should not be shared with any other trial participants or patients outside the trial. Further details will be provided in the Manual of Procedures (MOP).

4.6.2.5. Dose interruptions and discontinuations

Doses and concentrations will not be modified after the participant has been randomised. Consideration of temporary or permanent discontinuation of antiseptic use should be made at the discretion of the treating clinician in the following circumstances:
Mothers:

- Concerns over perineal skin/vaginal mucous membrane integrity: score >=3 in any category immediately before antiseptic application or >2 in 2 or more categories (see Appendix II)
- Any reason or change in clinical condition the treating clinician deems antiseptic application is not appropriate (i.e. antepartum haemorrhage)

Neonates:

- Hypothermia < 36.0C immediately before antiseptic application
- Skin score >2 in any category immediately before antiseptic application (see Appendix I)
- Neurological disturbance

When antiseptic is temporarily stopped for any reason, an assessment will be performed at the time of the next scheduled application and the clinician will determine whether it should be re-started. Treatment

interruptions and discontinuation and the reason for this should be recorded as appropriate on the trial CRFs.

4.6.2.6. Stopping CHG/OHP early (permanent discontinuation)

Stopping antiseptic permanently before the 6th application or birth (mothers) or discharge/72 hours (neonates) will be decided by the clinician. Possible reasons for permanent discontinuation would include relevant change in maternal or neonatal condition as decided by the clinician, or significant toxicity possibly related to the IMP as identified by the vaginal toxicity assessment and the neonatal skin score. Standard of care will then be delivered as decided by the local clinician.

4.6.2.7. Handling cases of overdose

For the purposes of this trial, overdose will be defined as more than 4 hourly in the mothers or more than once daily application in the neonates. Any additional doses given will be recorded on the treatment CRF and an AE form completed. Adverse reactions due to this would be expected to be similar to those described in Section 4.7.3 and will be recorded and reported in the same way as other adverse reactions (see Section 5).

4.6.2.8. Accountability and unused products

The antiseptic to be used for the trial will be stored separately from routine supplies in a designated section of the pharmacy, the ward or other appropriate location. The product will be designated for strict use for trial participants. The designated trial pharmacist or midwife will confirm receipt of supplies prior to the commencement of the trial. Inventories will be conducted regularly, and logs returned to the main pharmacy as detailed in the MOP. Inventories will comply with applicable local regulation, GCP and the protocol.

On no account should any product assigned to a participant be used for anyone else. Unused trial product must be returned to the main pharmacy if a participant is withdrawn from treatment before completing their randomised duration of therapy or if there is any remaining product. Procedures for product distribution, labelling, accountability and destruction will be detailed in the Pharmacy MOP. Product accountability will be regularly monitored and the remaining stocks checked against the amounts dispensed. At the end of the trial, all remaining products will be disposed of.

4.6.3. Control group

Control groups in both strata will receive routine clinical care. The current WHO recommendation during routine vaginal examination is to wash with clean water. Each antiseptic application that occurs will be recorded.

Any topical antiseptic or antimicrobial (all routes) that are used will be recorded on CRFs/worksheets, along with indication for its use.

Both control groups will receive swabs and toxicity assessments as per the schedule (see Section 4.7, Table 1, and Table 2). The control groups will receive regular assessments, including AE’s, the neonates will also receive daily temperature and skin assessments.

4.6.3.1. Compliance and adherence

If a participant in a control group receives antiseptic skin application, this may be due either to inadvertent application (e.g. due to error thinking the participant is in an intervention group), or due to routine use of antiseptic. Both will be recorded on trial CRFs. In the case of use for a specific purpose related to routine clinical care, this will be recorded but not classified as a protocol deviation, whereas if a participant inadvertently receives an application designed for a participant randomised into an intervention group, then this will be recorded as a protocol deviation.

4.6.4. Unblinding/unmasking

NeoVT-AMR is an open label trial and therefore there is no unblinding/unmasking.

4.6.5. Protocol treatment discontinuation

In consenting to the trial, mothers and parents/carers are consenting to trial treatment, trial follow-up and data collection. However, a participant may stop treatment early for any reason, including:

Both strata:

- Withdrawal of consent for treatment by the mother or parent/ carer
- Unacceptable toxicity or adverse event
- Intercurrent illness that prevents further treatment
- Any change in the participant's condition that justifies the discontinuation of treatment in the clinician's opinion
- Inadequate compliance with the protocol treatment in the judgement of the treating clinician

As participation in the trial is entirely voluntary, the mother or parent/carer may choose to discontinue the trial treatment at any time without penalty or loss of benefits to which they are otherwise entitled. Although the mother or parent/carer is not required to give a reason for discontinuing their trial treatment, a reasonable effort should be made to establish this reason while fully respecting their rights.

Participants should remain in the trial for the purpose of follow-up and data analysis (unless the mother or parent/carer withdraws their consent from all stages of the trial). If a participant is withdrawn from follow-up, refer to Section 4.7.4.

4.6.6. Compliance and adherence

Adherence to allocated interventions will be recorded on CRFs/worksheets.

4.6.7. Non-trial treatment

4.6.7.1. Medications permitted

After entry into the trial, any concomitant medication which is required is permitted, including any antimicrobials. All antiseptics and antibiotics will be recorded on CRFs.

4.6.7.2. Medications not permitted

All medications are permitted if necessary as deemed by participant's medical team.

4.6.7.3. Medications to be used with caution

Iodine has a known cosmetic interaction with octenidine. If applied within four hours of one another, the skin will discolour to a deep purple colour, which has no clinical significance and fades completely over

time. The exclusion criteria for both the labouring women and neonates exclude patients with any planned iodine use. In the case where iodine is needed and octenidine has been applied as part of the trial, chlorhexidine will be used instead of iodine. This is in line with latest WHO guidance.

Chlorhexidine may be required for routine care in any group, such as an invasive procedure or cord care. Whenever used outside of the trial procedures this will be recorded in the CRF and will not be considered as a protocol deviation if it is for routine care.

4.7. Assessments and follow-up

4.7.1. Trial assessment schedule

4.7.1.1. Maternal strata

Table 1: Trial procedures in maternal strata

Visit number	1		2	3	4	5	6	7	8	9
Visit name	Screening	Randomisation							Post delivery	Final follow up
Day in trial	0	0	0	0	0	1	1	1		28
Time in trial			0hr	4hr	8hr	24hr	28hr	32hr		
**All participant mothers**										
Eligibility	X									
Participant Information Leaflet	X									
Informed consent	X									
Enrolment form and randomisation		X								
Baseline data collection		X								
Newborn's swabs (peri-rectal/rectal + cervical/neck) - post delivery									X	
Final follow up										X
**Participants in single application arms**										
Antiseptic application			X							
Clinical assessment (toxicity + AE)			X	X	X	X	X	X	X	
Swabs (vaginal + perineal)			X	X	X	X	X	X		
**Participants in multiple application arms (4hrly)**										
Antiseptic application			X	X	X	X	X	X		
Clinical assessments (toxicity + AE)			X	X	X	X	X	X	X	
Swabs (vaginal + perineal)			X	X	X	X	X	X		
**Participants in control group**										
Clinical assessments (toxicity + AE)			X	X	X	X	X	X	X	
Swabs (vaginal + perineal)			X	X	X	X	X	X		

On days assigned to maternal recruitment, eligibility will be assessed by trial midwives. Labouring women who meet all of the inclusion and none of the exclusion criteria will be provided with a participant information leaflet, have the trial verbally explained and given the opportunity to have their questions answered. Informed consent will be recorded (Section 4.5.3).

An enrolment form will be completed and data entered into the database. Participants will be randomised (Section 4.5.5.1) and have baseline data collected, including medical history, gestational age, co-morbidities and antibiotic history.

Assessment and treatment regime will be according to allocation:

All maternal participants:

Toxicity assessment: the toxicity assessment will include both questions about symptoms (of irritation) and a visual inspection of the vulval area (for erythema, skin breakdown and swelling) by trial staff. The assessment will take place immediately before the swabs at 4 hourly intervals during working hours on day 1 and day 2, or until birth if sooner and post delivery. More details can be found in Section 4.7.3 and Appendix II.

Swab: Swabs (low vaginal and perineal) will be taken at baseline (0 hr) and then 4 hourly on day 1 and day 2 (during working hours), or until birth (whichever is first). The swab timing will be recorded in a CRF. See MOP for further details on how to take swabs.

If the vaginal and perineal area is heavily soiled prior to the swabs and antiseptic application, it will be cleaned, as per routine clinical practice. Further details can be found in the MOP.

Swab of babies: Babies born to enrolled mothers will have swabs (peri-rectal/rectal and cervical/neck) collected after birth to assess bacterial load. The time this is taken will be recorded.

Final follow-up: On day 28 (+/- 7 days) after enrolment, final follow up will occur either by phone call or hospital visit. If a hospital visit is chosen, appropriate compensation will be provided for travel and time in line with local standard policies. Vital status will be ascertained, together with occurrence of adverse events (including clinically defined sepsis and any unscheduled visits to healthcare providers) and follow up of any reported potential side effects that were present.

Maternal participants in single application arms:

Antiseptic: The antiseptic will be applied once to the vagina and perineum (more details Section 4.6.2.1) after the 1st assessment and swab. Following this, routine care at vaginal examination's will continue. Any antiseptic that is applied as part of routine care will be recorded on the CRF.

Maternal participants in multiple application arms:

Antiseptic: The antiseptic will be applied to the vagina and perineum 4 hourly during working hours, up to a maximum of 6 times (more details Section 4.6.2.1), after each swab and assessment of toxicity.

Maternal participants in control group:

Antiseptic application: any antiseptic that is applied as part of routine care will be recorded on the CRF. Antiseptic will be applied after the toxicity assessment and swabs.

Recruitment will continue until the sample size has been met. Missing swabs and incomplete follow up will be monitored closely and additional patients may be recruited to meet the required number of swabs.

4.7.1.2. Neonatal strata

Table 2: Trial procedures in neonatal strata

**Visit number**	**1**		**2**	**3**	**4**	**5**	**6**
**Visit name**	Screening	Randomisation	Intervention				Follow up
**Day in trial**	0		0	1	2	3	28
**All participant neonates**							
Eligibility	X						
Parent/carer information leaflet	X						
Informed consent	X						
Enrolment form and randomisation		X					
Baseline data collection	X						
Assessment and treatment according to allocation (see below)							
Final follow up							X
**Participants in single application arms**							
Clinical assessment (temperature, skin score + AE)			X	X	X	X	
Swabs (peri-rectal/rectal and cervical/neck)			X	X	X	X	
Antiseptic application			X				
**Participants in multiple application arm (24 hourly)**							
Clinical assessment (temperature, skin score + AE)			X	X	X	X	
Swabs (peri-rectal/rectal and cervical/neck)			X	X	X	X	
Antiseptic application			X	X	X	X	
**Participants in control group**							
Clinical assessment (temperature, skin score + AE)			X	X	X	X	
Swabs (peri-rectal/rectal and cervical/neck)			X	X	X	X	

Eligibility will be checked by trial staff. Parents/carers of neonates who meet all of the inclusion and none of the exclusion criteria will be provided with a participant information sheet, have the trial verbally explained and given the opportunity to have their questions answered. Informed consent will be recorded (Section 4.5.3).

An enrolment form will be completed and data entered into the database. Participants will be randomised (details in Section 4.5.5.1) and have baseline data collected, including antenatal history, estimated gestational age, birth weight, labour duration, mode of delivery, co-morbidities, and intrapartum and postpartum antibiotic history.

Assessment and treatment regime will be according to allocation:

All neonatal participants:

Skin score: Skin will be assessed according to the skin score in Appendix I before each swab or antiseptic application at screening and once daily thereafter (at 24 and 48 hours) until discharge or 72 hours. For babies assigned to antiseptic application, skin score will also be assessed 30 minutes after antiseptic application.

Swab: Swabs (peri-rectal/rectal and cervical/neck) will be taken at baseline (0 hrs) and then at 24 hours, 48 hours, and 72 hours, or until discharge (whichever is sooner).

The timing of swabs will be recorded in a CRF. See MOP for further details on how to take swabs.

Final follow-up: On day 28 (+/- 7 days) after enrolment final follow up will occur either by phone call or hospital visit. If a hospital visit is chosen by the parents/carers, appropriate compensation will be provided for travel and time in line with local standard policies. Vital status will be ascertained, together with occurrence of serious adverse events (including clinically defined sepsis and any unscheduled visits to healthcare providers).

Neonatal participants in single application arms:

Temperature: Temperature will be measured before antiseptic application, 15 minutes post application and once daily before the swabs thereafter until discharge or 72 hours.

Antiseptic: The antiseptic will be applied to the whole body (except face) (more details Section 4.6.2.3) after the 1st swabs.

Neonatal participants in multiple application arms:

Temperature: Temperature will be measured before every antiseptic application and 15 minutes post application.

Antiseptic: The antiseptic will be applied to the whole body (except face) after each assessment and swabs: at baseline, and at 24 hours, 48 hours and 72 hours or discharge (more details Section 4.6.2.3).

Neonatal participants in control group:

Temperature: Temperature will be recorded at baseline and once daily before the swabs thereafter until discharge or 72 hours.

4.7.2. Microbiology procedures for assessing efficacy

Efficacy will be measured as change in colony forming units (CFUs) from baseline (the swabs at randomisation before antiseptic application).

To allow comparison across groups, all women in labour (regardless of randomisation group) will have a swabs taken at baseline (0 hrs), 4 hours, 8 hours, 24 hours, 28 hours, and 32 hours and all neonates (regardless of randomisation group) will have swabs taken at baseline, 24 hours, 48 hours and discharge or 72 hours.

Additionally, to provide an estimate of the effect of maternal intervention in the newborn, babies born to mothers in the maternal stratum will have swabs (peri-rectal/rectal and cervical/neck) taken after birth. The timing of these swabs will be recorded on a CRF.

Table 3: Schedule of swabs from maternal and neonatal strata

	**Maternal Strata***			**Newborns born to mothers from Maternal Strata#**			**Neonatal Strata***		
**Time from intervention**	Frequency of intervention			Frequency of intervention			Frequency of intervention		
	Single	Multiple	Control	Single	Multiple	Control	Single	Multiple	Control
**Post Delivery**				✓	✓	✓			
**Baseline**	✓	✓	✓				✓	✓	✓
**4 hours**	✓	✓	✓						
**8 hours**	✓	✓	✓						
**12 hours**									
**16 hours**									
**20 hours**									
**24 hours**	✓	✓	✓				✓	✓	✓
**28 hours**	✓	✓	✓						
**32 hours**	✓	✓	✓						
**48 hours**							✓	✓	✓
**72 hours**							✓	✓	✓

*Participants will be swabbed as per schedule or until delivery/birth (maternal strata) or discharge from the hospital (neonatal strata), whichever comes first.

#Newborns born to women from the maternal strata will be swabbed only once immediately after birth and will not receive any interventions.

Swabs will be vortexed in liquid transport medium, inoculated in serial dilutions on MacConkey and blood agar, and read after 24 hours incubation. Both total bacterial load (total CFU) and pathogen species will be recorded. Simultaneous inoculation onto extended spectrum beta-lactamase producing selective CHROMagar will be used to identify ESBL-E. Species identity will be confirmed by API.

Consent will be gained for use of samples in future microbiological analyses.

Swabs will be processed in the Malawi-Liverpool-Wellcome Trust microbiology laboratory, Blantyre. They will be securely couriered there from the trial site, using appropriate methods to control sample temperature. Two swabs will be taken and one disposed of after primary microbiological processing, and clones of bacteria will be kept in the Wellcome Trust research laboratory, Blantyre. The second

DocuSign Envelope ID: B928C439-2A10-4CA0-932F-B711DAAFBA4E

swab will be retained for up to 5 years to permit future microbiological analysis of the vaginal microbiota. Further details are outlined in the MOP.

4.7.3. Procedures for assessing safety

Mothers:

The summary of product characteristics for Octenisept® states that the undesirable effects of burning, redness, itching and warmth at application site occur rarely (>1/10,000 <1/1,000) and allergic contact reaction occurs very rarely (<1/10,000). The summary of product characteristics for CHG states that allergic or irritation reactions such as erythema, rash, pruritis, blisters and local symptoms such as burning sensation, pain and inflammation occur very rarely (<1/10,000).

The incidence of these events has not been studied specific to vaginal and perineal application. These will be monitored for at the 4 hourly toxicity assessments (as described in Appendix II). Toxicity assessments will be performed at the same time points for all enrolled women, to provide unbiased comparisons across randomised groups. The toxicity score will be considered as both an absolute value and graded.

Adverse events will be checked for regularly during admission. They will be reported as described in Section 5. The final follow up at day 28, which will be over telephone or in person visit, will check for adverse events and status of toxicity reported during admission.

Neonates:

Safety will be assessed using a modified skin score which assesses the skin condition, and presence of erythema, dryness, and any other sign of skin damage (see Appendix I)(46,47). This will be performed before each application of antiseptic and 30 minutes after application. The skin score will be considered as both an absolute value and graded. Skin score assessments will be performed once every day on all babies to provide unbiased comparisons across randomised groups.

Temperature will be monitored before and 15 minutes post every antiseptic application. For neonates in the once only and control arms temperature will be measured daily. Guidelines for repeating the temperature if it is out of range will be outlined in the clinical MOP.

Hypothermia will be graded as follows:

- Grade 1: 35.5 - 36.0C
- Grade 2: 35.4 - 35.0C
- Grade 3: 34.0 - 34.9C
- Grade 4: <34.0C

Other adverse events will be monitored for daily and reported as described in Section 5.

4.7.4. Early stopping of follow-up

If a mother or parent/carer (who gave consent) chooses to discontinue their own or their baby’s trial treatment, the participant should be encouraged not to leave the whole trial to allow follow up. If the mother or parents/carers do not wish themselves or their baby to remain on trial follow-up, however, their decision must be respected, and the participant will be withdrawn from the trial completely. The SGUL team and MLW trial team should be informed of this in writing via email and early stopping detailed in the CRF.

The medical data collected during the neonates or mother's participation in the trial will be kept for research and analysis purposes, in a pseudonymised format (identified only by their trial number).

4.7.5. Patient transfers

If a participant moves from the trial hospital to another hospital during the trial, further application and assessments related to the trial will not be continued at the other hospital.

4.7.6. Follow-up

Upon discharge from Zomba hospital, mothers and parents/carers will be provided with the trial team's contact details and information on signs to monitor for and inform the trial team about. They will be advised to return to hospital if they have any concerns. Swabs and antiseptic application will not continue after discharge and participation will not impact duration of inpatient stay. Final follow up for both groups will occur at 28 days (+/- 7 days) via telephone, or at a visit in the hospital. Compensation for time and travel to the hospital will be provided.

4.7.7. Recruitment team

Recruitment will be led by research midwives who will be employed by MLW. They will be responsible for approaching and recruiting participants, and for completion of trial procedures. Training in trial methodology will be led by the trial management group in collaboration with MLW. Training on GCP and clinical trials methodology will be led by MLW. This will include training before the trial commences, and regular refresher training and quality control exercises.

4.8. Data Collection and Generation

4.8.1. Methodologies for data collection/generation

Baseline, treatment and follow up data including information about all antibiotics and concomitant topical medication received will be recorded on CRFs from recruitment to final follow up at day 28.

Source data in Malawi will be collected electronically directly into REDCap™ databases specifically designed for the trial by the data management team in Malawi, with additional installation, operational and performance qualification developed by the Malawi data team. REDCap™ is a secure web-based application. Clinical and demographic data will be entered in real-time in English. Clinical samples will be linked to participant data through individually issued barcode labels. Laboratory data will be managed using the preLINK LIMs system. Wherever possible laboratory data will be uploaded electronically or using barcodes to avoid transcription errors.

4.8.2. Data quality and standards

Electronic data capture forms will be compliant with regulatory requirements for clinical trials, following the principles of ICH-GCP and the UK Data Protection Act 2018 and GDPR. Standard operating procedures (SOPs) will be developed and followed to ensure consistency and transparency across the teams and personnel that will be handling the data and to ensure the correct and confidential management of data. On recruitment all participants will be given a unique trial identification number and their data will be uncoupled from personal identifiers. Data will be collected and stored in accordance with international GCP guidelines and SGUL information governance and data management policies.

4.9. Data Management and Quality Assurance

4.9.1. Central monitoring

The site will be responsible for its own data entry and local trial management. Data will be entered into the trial database directly at the site. Data stored on the central REDCap™ database will be checked by SGUL or delegated team for missing or unusual values (range checks). If any problems relating to data quality are identified, the site will be contacted and asked to verify or correct the entry. Reminders will be sent for any overdue and/or missing data with the regular inconsistency reports of errors.

Other essential trial issues, events and outputs will be detailed in the Monitoring Plan that is based on the trial-specific Risk Assessment.

4.9.2. On site monitoring

The frequency, type and intensity for routine monitoring and the requirements for triggered monitoring will be detailed in the Monitoring Plan. This plan will also detail the procedures for review and sign-off.

A detailed site initiation meeting with training will be performed at the trial site by staff from MLW either face to face or online. The site initiation meeting will include training in the administration and toxicity profile of trial products, as well as the trial procedures.

4.9.3. Direct access to patient records

Participating investigators should agree to allow trial-related monitoring, including audits, ethics committee review and regulatory inspections by providing direct access to the REDCap database, source data and documents as required. Mothers and parental/caregiver consent for this must be obtained. Such information will be treated as strictly confidential and will in no circumstances be made publicly available.

The trial data and consent should all be verifiable from source documents which may include paper notes.

The investigator/institution should maintain adequate and accurate source documents and trial records that include all pertinent observations on each of the participants. Source data should be attributable, legible, contemporaneous, original, accurate, and complete. Changes to source data should be traceable, should not obscure the original entry, and should be explained if necessary (e.g., via an audit trail).

4.9.4. Data management, documentation and curation

Data capture forms will be designed with validity criteria built in to minimise data entry error and regular data checks will be conducted to identify data discrepancies and errors using specifically designed algorithms and data checks. This will be managed by the Malawi data management team with oversight from the trial PI. Data will be stored for 20 years as per SGUL's research data management and records management policies and Medical Research Council "Good Research Practice: Principles and Guidelines" July 2012.

DocuSign Envelope ID: B928C439-2A10-4CA0-932F-B711DAAFBA4E

4.9.5. Data security and confidentiality

4.9.5.1. Formal information/data security standards

On recruitment all participants will be given a unique trial identification number and their data will be uncoupled from personal identifiers. The electronic data will be held on a secure server at MLW, with data extracted periodically to a secure server at SGUL. Data will be exported to CSV format to enable sharing and long-term validity of the data. Only the trial PIs, trial data management team, and the statistical team will have access to the data.

All data will be collected, processed and retained in accordance with SGUL's information governance and data management policies. SGUL's information security policies, procedures and standards are compliant with the NHS Data Security and Protection Toolkit and are Cyber Essentials Plus certified. SGUL and MLW are REDCap™ consortium partners each having a dedicated server which uses Secure Sockets Layer to protect data in transit.

4.9.5.2. Main risks to data security

Data will be captured in the field and may need to be temporarily kept on trial electronic devices **-** in this instance the electronic devices will be encrypted and password protected. To mitigate the risks of data loss, data on the server will be backed up daily by both MLW and SGUL IT network onto storage disk and tape storage.

4.9.5.3. Confidentiality

We plan to follow the principles of the EU General Data Protection Regulation (GDPR) as well as being compliant with data laws in Malawi. In particular, the investigator must ensure that participant's anonymity will be maintained and that their identity is protected from unauthorised parties. Participants will be assigned a trial identification number and this will be used on CRFs; they will not be identified by name. The investigator will keep securely a trial register showing trial identification numbers, initials and date of admission, date of birth and age at admission, held only at the local site. The unique trial number (or laboratory tracking number) will identify all laboratory specimens, CRFs, and other records and no names or initials will be used on forms or samples, in order to maintain confidentiality. Date of birth will be collected as a data item since all babies will be under 28 days old and hence is needed to calculate age precisely. Names will be written on signed informed consent documents, but these will be stored separately to CRFs.

All records will be kept in locked locations. Clinical information will not be released without written permission, except as necessary for monitoring by the trial monitors.

4.9.6. Data sharing and access

4.9.6.1. Suitability for sharing

To maximise the scientific outputs of the trial anonymised data may be shared with collaborators and the wider research community.

4.9.6.2. Discovery by potential users of the research data

Due to the potential sensitivity of the data, the data will be made available on a controlled access basis, with descriptive information on the data publicly available on clinical trial and institutional repositories along with information on how researchers will be able to access the data.

4.9.7. Data Governance of access

This will normally be at the approval of the trial management group, in accordance with SGUL policy for

DocuSign Envelope ID: B928C439-2A10-4CA0-932F-B711DAAFBA4E

controlled data access. Controlled access data will be shared under standard data sharing agreements.

4.9.8. Restrictions or delays to sharing, with planned actions to limit such restrictions

Prior to sharing all data will be made anonymous. Trial participants will be consented for anonymous sharing of their data at the point of recruitment.

4.10. Statistical considerations

4.10.1. Method of randomisation

Randomisation codes will be generated using a random number generator, as implemented in common statistical software packages. The randomisation codes will be kept on an electronic database which the mobile device used by the clinical staff will connect to when they initiate the randomisation of a participant. The mobile device will, during this connection, retrieve the randomised treatment group allocation for the next participant on the randomisation list. The design will not be blinded as this is not practical given the factors evaluated. However, the efficacy endpoint (bacterial colonisation) will be assessed by laboratory staff blinded to randomisation.

4.10.2. Outcome measures

4.10.2.1. Co-primary outcome measures

Mothers:

- Change in vaginal and perineal bacterial load (in colony forming units)

Neonates:

- Change in neonatal skin bacterial load (in colony forming units)

4.10.2.2. Secondary outcome measures

Mothers:

- Maternal toxicity – score and grade (tolerability and safety)
- Bacterial load in neonates exposed to maternal antiseptic, compared to control
- Serious adverse events

Neonates:

- Adapted neonatal skin condition score (safety) (absolute score and grade)
- Temperature (change in absolute temperature and grade (hypothermia))
- Serious adverse events

4.10.3. Interim monitoring and analyses

A DMC Charter will be drawn up that describes the membership of the DMC, terms of reference, decision-making processes, and the timing and frequency of meetings. The DMC will meet around the time the trial starts (without reviewing any data) to ensure they are familiar with its design and conduct. They will formally review data halfway through planned recruitment. They may call additional meetings at any time at their discretion. In addition, SARs will be sent in real time during the trial to the DMC.

The DMC can recommend premature closure or reporting of the trial, or that recruitment to any randomised group be discontinued or modified. Such recommendations would be made if, in the view of the DMC, there is an unacceptable rate of adverse reactions. There will be no early stopping for

clinical efficacy because this pilot trial is powered only to detect differences in skin bacterial load, not clinical efficacy outcomes. See Section 8.2 for details on DMC membership.

4.10.4. Analysis plan (brief)

The analyses will be described in detail in a full Statistical Analysis Plan. This section summarises the main issues.

The primary analysis population is intention-to-treat, including all randomised participants, regardless of treatment received. This corresponds to estimating the impact of the effectiveness of the treatments. However, in secondary analyses we will also use inverse-probability weighting methods to adjust for deviation from randomised strategy, which is a more efficient approach than defining a per-protocol population.[39]

Analysis of the co-primary endpoints will compare within-individual differences over time across randomised arm to maximise the information gained from the trial. Analysis will use linear mixed-models, with individual as a random effect, will adjust for baseline values of each outcome, and use Normally distributed errors. Analysis will be both frequentist, enabling straightforward comparison between arms (primary analysis since sample size is based on this), and Bayesian, enabling estimation of the probability that one arm is truly superior to another. This is more powerful when sample sizes are small as use of priors provides additional information. Sensitivity analysis to prior assumptions will be performed using optimistic, non-informative and sceptical priors.

The effect of each treatment arm will be assessed within a single model with each factor being fitted independently as a factor, and not as a continuous variable. Comparison of main effects within arms will be assessed using the estimate and 95% confidence interval of the comparison of differences between factors and in comparison, to control. As this is a pilot trial, the sample size was not powered to robustly detect interactions between treatment and frequency and so we will not test these formally, but will use exploratory analyses to assess whether there is any evidence supporting individual interactions testing each one, one at a time.

Similar mixed models will be used to analyse change in skin temperature after vs before each application, including individual as a random effect, the change in temperature as the outcome and the pre-application (baseline) temperature as a factor.

Frequency of SAEs will be tabulated by randomised group, and compared using exact logistic models (or time-to-event models if AEs occur in >10% of the trial population overall). SAEs will also be tabulated by grade. Similar analyses will be conducted for the AEs in Appendix III.

A Statistical Analysis Plan will be written and approved by the DMC before the first interim analysis is reviewed by the DMC.

4.11. Results Presentation and Dissemination of Findings

The TMG is responsible for access to the data and bacterial isolates generated from the NeoVT-AMR trial; trial data are not the property of individual participating investigators or healthcare facilities where the data were generated.

- Publications include papers (including abstracts) for presentation at national and international meetings, as well as the preparation of manuscripts for peer-reviewed publication. In order to avoid disputes regarding authorship, it is important to establish a consensus approach that will provide a framework for all publications derived in full or in part from this clinical trial.

- All publications are to be approved by the TMG before submission for publication. Any publication arising before the end of the trial (not by randomised groups) will also be approved by the DMC in order to ensure that the primary objective of the trial (the randomised comparison) is not compromised. The TMG will resolve problems of authorship and maintain the quality of publications.

- Results of publicly-funded research should be freely available, manuscripts arising from the trial will, wherever possible, be submitted to peer-reviewed journals which enable Open Access via UK PubMed Central (PMC) within six months of the official date of final publication.

- For all publications, the TMG will nominate a chairperson or approve an individual's request to chair a manuscript writing committee. The chair will usually be the primary or senior author. The chairperson is responsible for identifying fellow authors and for determining with that group the order of authorship that will appear on the manuscript. The TMG will resolve any problems of authorship and maintain the quality of publications.

- The TMG will maintain a list of investigators to be presented in an appendix at the end of the paper. This list will include investigators who contributed to the investigation being reported but who are not members of the writing committee, together with all relevant expert advisors and members of the DMC. All families who participated in the trial will be thanked as a group (not by name). In principle, sub-study reports should include all investigators for the main trial, although in some instances where a smaller number of investigators have made any form of contribution, it may be appropriate to abbreviate the listing. All headline authors in any publication arising from the main trial or sub-studies must have a made a substantive academic or project management contribution to the work that is being presented. "Substantive" must be defined by a written declaration of exactly what the contribution of any individual is believed to have been. In addition to fulfilling the criteria based on contribution, additional features that will be considered in selecting an authorship group will include the recruitment of babies who contributed data to any set of analyses contained in the manuscript and/or the conduct of analyses (laboratory and statistical), leadership and coordination of the project in the absence of a clear academic contribution.

- The data derived from this clinical trial are considered the property of the Sponsor. The presentation or publication of any data collected by the participating investigators on participants entered into this trial is under the direct control of the TMG (and the DMC before the end of the trial). This is true whether the publication or presentation is concerned directly with the results of the trial or is associated with the trial in some other way. However, although individual participating investigators will not have any inherent right to perform analyses or interpretations or to make public presentations or seek publication of any of the data other than under the

auspices of and with the approval of the TMG (and the DMC before the end of the trial), they will be encouraged to develop sub-studies or propose analyses subject to the approval by the TMG (and the DMC before the end of the trial). Any requests for access to raw data will be welcomed as long as they are scientifically valid and do not conflict with the integrity of the trial or ongoing analyses by the trial team

The research will be conducted at the Malawi-Liverpool-Wellcome Trust clinical research programme (MLW). This institute has a strong history of collaborative research with the local community and has a very strong science communication team.

A copy of the final report and any published paper(s) or abstracts of papers read at conferences out of the research findings will be submitted to each of the following:-

- The College of Medicine Research and Ethics Committee (COMREC)
- College of Medicine Library
- The Health Sciences Research Committee (through the COMREC Secretariat)
- The University Research and Publication Committee (URPC) (through the COMREC Secretariat)

Research findings will be disseminated via presentations at local and international conferences and scientific meetings, including the annual Malawi College of Medicine research dissemination day, Results will be published on institute, University and hospital websites.

To safeguard against possible bias affecting the data collection, outcome data by randomised group will not be revealed to the participating investigators until the data collection phase and primary full analysis of the trial has been completed. The DMC will be monitoring the outcome results and may recommend that the trial be stopped for safety reasons.

5. Safety reporting

The principles of GCP require that both investigators and Sponsors follow specific procedures when notifying and reporting adverse events or reactions in clinical trials. These procedures are described in this section of the protocol. <u>Section 5.1</u> lists definitions, <u>Section 5.2</u> gives details of the investigator responsibilities and <u>Section 5.3</u> provides information on the SGUL responsibilities.

5.1. Definitions

The definitions of the EU Directive 2001/20/EC Article 2 based on the principles of GCP, which is in line ICH E2A apply to this trial protocol. These definitions are given in Table 3.

Table 4: Definitions

TERM	DEFINITION
Adverse Event (AE)	Any untoward medical occurrence in a patient or clinical trial subject to whom a medicinal product has been administered including occurrences that are not necessarily caused by or related to that product.
Adverse Reaction (AR)	Any untoward and unintended response to an investigational medicinal product related to any dose administered.

Unexpected Adverse Reaction (UAR)	An adverse reaction, the nature or severity of which is not consistent with the information about the medicinal product in question set out in the Summary of Product Characteristics (SmPC) or Investigator Brochure for that product.
Serious Adverse Event (SAE) or Serious Adverse Reaction (SAR) or Suspected Unexpected Serious Adverse Reaction (SUSAR)	Respectively any adverse event, adverse reaction or unexpected adverse reaction that:<br>▪ Results in death<br>▪ Is life-threatening [a]<br>▪ Requires hospitalisation or prolongation of existing hospitalisation [b]<br>▪ Results in persistent or significant disability or incapacity<br>▪ Consists of a congenital anomaly or birth defect<br>▪ Is another important medical condition [c]<br>▪ Cancer – new onset and recurrent [d]<br>▪ Immune dysfunction – autoimmune and immune deficiency diseases [d]

[A] The term life-threatening in the definition of a serious event refers to an event in which the patient is at risk of death at the time of the event; it does not refer to an event that hypothetically might cause death if it were more severe, for example, a silent myocardial infarction.

[B] Hospitalisation is defined as an inpatient admission, regardless of length of stay, even if the hospitalisation is a precautionary measure for continued observation. Hospitalisations for a pre-existing condition, that has not worsened or for an elective procedure do not constitute an SAE.

[c] Medical judgement should be exercised in deciding whether an AE or AR is serious in other situations. The following should also be considered serious: important AEs or ARs that are not immediately life-threatening or do not result in death or hospitalisation but may jeopardise the subject or may require intervention to prevent one of the other outcomes listed in the definition above; for example, a secondary malignancy, an allergic bronchospasm requiring intensive emergency treatment, seizures or blood dyscrasias that do not result in hospitalisation or development of drug dependency.

[d] The Pharmacy and Medicines Regulatory Authority of Malawi recognise Cancer and Immune Dysfunction as reportable serious adverse experiences

5.1.1. Product

The trial is testing CHG and OHP for topical use on the perineum and vagina of labouring women or the skin of neonates.

5.1.2. Adverse events

Adverse Events include:

- An exacerbation of a pre-existing illness
- An increase in frequency or intensity of a pre-existing episodic event or condition
- A condition (even though it may have been present prior to the start of the trial) detected after trial product administration
- Continuous persistent disease or a symptom present at baseline that worsens following administration of the trial treatment

Assessment of adverse events (AEs) is challenging in neonates since standard adult grading scales do not apply; for example, normal ranges of laboratory parameters vary with age and normal function is difficult to specify. Adverse events will therefore be assessed using an adapted version of the DAIDS

scale for neonates, that has been used in previous neonatal trials by groups such as IMPAACT(48), including studies conducted for regulatory FDA approval (Appendix III).

Each adverse event will be assessed at each scheduled assessment and graded by the investigator according to the Adapted DAIDS score for neonates, reproduced from the IMPAACT network(48) and maternal adverse event grading, reproduced from the "Common Terminology Criteria for Adverse Events (CTCAE)" (Appendix III).

Maternal toxicity and neonatal skin reactions and hypothermia will be separately assessed as part of secondary outcomes and graded according to the classification shown in Appendix I, II and Section 4.7.3.

5.1.3. Safety management

In the case of adverse events, standard operating procedures will be available for events including hypothermia, skin damage, as well as rare and highly unlikely events such as splash injury to the eye from antiseptic and allergic reactions.

5.1.4. Exempted adverse events

Adverse Events do not include:

- Medical or surgical procedures associated with delivery (including caesarean sections)
- Pre-existing disease or a condition present before treatment that does not worsen
- Hospitalisations where no untoward or unintended response has occurred, e.g., social admissions
- Overdose of medication without signs or symptoms

5.2. Investigator responsibilities

Presence of non-serious AEs in Appendix III will be documented in CRFs at each scheduled visit.

All AEs meeting the seriousness criteria defined in Table 4, should be notified to SGUL using the SAE Form within 24 hours of the investigator becoming aware of the event.

5.3. Investigator assessment

5.3.1. Seriousness

When an AE occurs, the trial team member responsible for the care of the participant must first assess whether or not the event is serious using the definition given in Table 3. If the event is serious, then an SAE Form must be completed within 24 hours of the investigator becoming aware of the event.

5.3.2. Severity or grading of adverse events

AEs, serious or not serious, should be graded using the Adapted DAIDS score for neonates, reproduced from the IMPAACT network[38] (Appendix III) or the maternal adverse event grading, reproduced from the "Common Terminology Criteria for Adverse Events (CTCAE)" (Appendix III).

5.3.3. Causality

The investigator must assess the causality of all serious events in relation to the trial treatment using the definitions in Table 4. There are five categories: unrelated, unlikely, possible, probable, and definitely related. If the causality assessment is unrelated or unlikely to be related, the event is classified as an

SAE. If the causality is assessed as possible, probable or definitely related, then the event is classified as an SAR.

Table 5: Assigning Type of SAE Through Causality

RELATIONSHIP	DESCRIPTION	SAE TYPE
Definitely	There is clear evidence to suggest a causal relationship and other possible contributing factors can be ruled out.	SAR
Probable	There is evidence to suggest a causal relationship and the influence of other factors is unlikely.	SAR
Possible	There is some evidence to suggest a causal relationship (for example, because the event occurs within a reasonable time after administration of the trial medication). However, the influence of other factors may have contributed to the event (for example, the patient's clinical condition, other concomitant treatments).	SAR
Unlikely	There is little evidence to suggest that there is a causal relationship (for example, the event did not occur within a reasonable time after administration of the trial medication). There is another reasonable explanation for the event (for example, the patient's clinical condition, other concomitant treatment).	Unrelated SAE
Unrelated	There is no evidence of any causal relationship	Unrelated SAE

If an SAE is considered to be related to trial treatment and CHG or OHP is stopped, refer to Section 4.6.2.5. for more information on dose interruptions and discontinuations.

5.3.4. Notification

SGUL, the College of Medicine Research and Ethics Committee (COMREC) and the Pharmacy and Medicines Regulatory Authority (PMRA) should be notified of all SAEs within 24 hours of the investigator becoming aware of the event. Investigators should provide notification of all SAEs occurring from the time of signature of the informed consent form until 28 days after randomisation.

The SAE Form must be completed by a trained clinical team member (named on the Signature List and Delegation of Responsibilities Log, who is responsible for the participant's care; this will be either the Principal Investigator or another medically qualified person with delegated authority for SAE reporting). Due care should be paid to the grading, and assessment of causality of the event, as outlined above. In the absence of the responsible investigator, the form should be completed and signed by a member of the site trial team and emailed to neovtamr@sgul.ac.uk. The initial report must be followed by detailed, written reports as appropriate.

The minimum criteria required for reporting an SAE are the trial number and date of birth, name of investigator reporting, the event, and why it is considered serious.

Follow-up: participants must be followed up until clinical recovery is complete or until the event has stabilised. Follow-up should continue after completion of protocol treatment if necessary. A further SAE

Form, indicated as ‘Follow-up SAE Form’ should be completed and emailed to the SGUL team as information becomes available (or where a small amount of fields are updated, the original SAE form may be modified following GCP). Extra, annotated information and/or copies of test results may be anonymised and provided separately. The participant must be identified by trial number and date of birth. The participant’s name must not be used on any correspondence and should be deleted from any test results.

Staff should follow the Zomba Central Hospital’s procedure for local notification requirements and the reporting timelines as outlined for local applicable regulations. This will be further detailed in the SOP.

SAE REPORTING
Within 24 hours of becoming aware of an SAE, please email a completed SAE form to neovtamr@sgul.ac.uk.

5.4. SGUL (sponsor) responsibilities

Medically-qualified staff at SGUL and/or the Chief Investigator (or a medically-qualified delegate) will review all SAE reports received and assess expectedness as necessary. The causality assessment given by the local investigator at the hospital cannot be overruled; in the case of disagreement, both opinions will be provided in any subsequent reports.

6. Ethical considerations

6.1. Compliance

6.1.1. Regulatory compliance

The trial complies with the principles of the 1996 version of the Declaration of Helsinki. It will also be conducted in compliance with the approved protocol, the principles of ICH Good Clinical Practice (GCP) as laid down by the ICH topic E6 (R2).

6.1.2. Site compliance

The site will comply with the above and also with applicable national guidelines. The site will inform the sponsor as soon as they are aware of a possible serious breach of compliance, so that they can report this breach if necessary as per relevant regulatory requirements. For the purposes of this regulation, a 'serious breach' is one that is likely to affect to a significant degree:

- The safety or physical or mental integrity of the subjects in the trial, or
- The scientific value of the trial.

6.2. Ethical conduct

6.2.1. Ethical considerations

By necessity this trial recruits potentially vulnerable groups (labouring women, neonates). Every care will be taken to respect women and newborns at this time and to ensure that recruitment and participation does not interfere with clinical care.

The autonomy of women and parents/carer's will be protected throughout the trial and during the informed consent process. Risk of coercion will be reduced by separating the role of the clinical team and the researchers, with the clinical team acting as advocates for their patients. Care will be taken to gain and record written informed consent at an appropriate time before enrolment. Before consent is obtained, women and parents/carers will be fully informed of known risks and possible benefits by a participant information leaflet, and this will be reinforced by discussions with the trial research team. Additionally, the woman and their partner will have opportunity to discuss the study and have any questions answered. Women who are already known to be in active labour will not be approached by the research midwife, and will not be eligible for inclusion. Further information is in Section 4.5.3 (Section 4.5.3).

Antiseptic and swabs will be administered by trial staff at the time of planned vaginal examinations to minimise interruption to clinical care. Condition of the external mucous membranes will be examined prior to administration and antiseptic will not be applied if they are damaged or inflamed or if the participant has reported any severe side effects.

One ethical concern in this trial relates to potential skin or mucous membrane damage. Antiseptics, including CHG and OHP, are widely used for skin disinfection in neonates and mothers and are typically well tolerated. The main risk is with high concentrations (>2%) in extremely low birth weight/preterm neonates (<1kg/<28 weeks), among whom skin reactions have been observed, particularly when chlorhexidine is combined with alcohol. Hypothermia has also been observed after skin cleansing with chlorhexidine on the first day of life. This trial will exclude neonates less than 1kg at enrolment which will minimise, although not entirely eradicate these risks. However, close and regular monitoring of the

skin with a standardised skin condition scoring system, and temperature checking after application will allow chlorhexidine to be discontinued at the discretion of the local clinician.

Importantly, safety concerns are one of the main rationale for this pilot trial, in order to identify the safest but most effective regimen which could be tested in a larger trial aiming to reduce neonatal sepsis and mortality.

Safety will be closely monitored throughout the trial by an independent data and safety monitoring committee, and interventions will be stopped if there are any concerns regarding safety.

Confidentiality of the mothers, newborns and their parents/care-givers will be maintained throughout the trial. Data submitted on CRFs at the trial site and SGUL will be identified only by the trial number.

The final follow up will occur on day 28 by telephone or an in person visit at Zomba Central Hospital. If participants or parents/carers do not have access to a telephone, they will be asked to attend for a short visit at the hospital. They will be offered appropriate compensation (K1200) for this day 28 follow up. This is in line with local policies to reimburse their travel expense and time spent at the visit (which is anticipated to take <30 minutes), but not too much as to incentivise participation in the trial(49).

For the staff involved in the trial, no risk is expected as appropriate personal protective equipment, such as gloves, will be used for antiseptic application.

6.2.2. Ethical approvals

Prior to initiation of the trial at clinical sites, the protocol, consent forms and information materials to be given to participants will be submitted for ethical approval in Malawi - the COMREC and in the UK – SGUL ethics committee. Following ethical approval, all documents will be submitted to the PMRA for approval. Any amendments will be submitted for approval.

6.3. Trial closure

End of trial is defined as 18 months after the last scheduled follow-up visit of the last randomised participant. This is to ensure sufficient time for data submission, data cleaning, verification of queries, database lock and final analysis. The trial site will be closed once data cleaning is completed at that site, and the ethics committee and any relevant regulatory authorities will be informed.

7. Indemnity

The Sponsor of the trial is St George's, University of London. In consideration of the agreement by the Principal Investigator at the site to supervise the trial, the sponsor undertakes to indemnify the Principal Investigator and the institutions which participate in the trial and their employees and agents in respect of any claims made against them by any third party which arises out of or as a result of the supervision or conduct of the trial (including any claim arising in respect of the technical procedures described in the protocol which participants would not have been exposed to but for their participation in the trial). Full details of the Indemnity agreement are given in a separate document. Cover against claims arising from medical negligence is not included.

8. Oversight and trial committees

There are several groups involved with the oversight of the trial, detailed below.

8.1. Trial management group (TMG)

A Trial Management Group (TMG) will be formed comprising the Chief Investigator, other lead investigators (clinical and non-clinical) and members of SGUL. The TMG will be responsible for the day-to-day running and management of the trial.

8.2. Independent data monitoring committee (DMC)

An independent Data and Safety Monitoring Committee (DMC) will be formed. The Chair will be Professor Elizabeth Molyneux, the independent member will be Professor Pisake Lumbiganon (Khon Kaen University) and the statistician will be Marc Henrion (Malawi Liverpool Wellcome Trust). The DMC will be the only group who sees the confidential, accumulating data for the trial. Reports to the DMC will be produced by the trial statistician(s). The DMC will meet at the beginning of trial to become familiar with the protocol, then approximately halfway through expected recruitment. The DMC will consider data using the statistical analysis plan (see Section 4.10.4) and will advise the TMG. The DMC can recommend premature closure or reporting of the trial, or that recruitment to any randomised arm be discontinued.

Further details of DMC functioning, and the procedures for interim analysis and monitoring are provided in the DMC Charter.

8.3. Role of the trial sponsor

St George's, University of London, is the Sponsor. Responsibility for implementation of the trial, including arrangements for adequate management, monitoring, including safety, and reporting will be delegated to MLW. Data analysis and reporting is delegated to the MRC CTU.

9. Patient and public involvement

There are no existing patient and public involvement groups for those admitted to the labour ward, postnatal ward or the neonatal unit at Zomba Central Hospital. The time of labour, birth and the postnatal period is a sensitive area of clinical research. Within this pilot trial, there is no specific resource to set up formal patient and public involvement, but we will explore with the participating women and parents/carers of babies recruited into this pilot trial their potential interest in being involved in future engagement around a larger trial, particularly in terms of the potential acceptability of the intervention, and differing cultural practices around use of skin products in newborn babies and in labouring women. Therefore, this pilot trial will assess the feasibility of establishing parent/carer groups and identifying possible parent/carer representatives for a future larger trial.

9.1. Possible Constraints

All clinical aspects, from recruitment to follow up, will be conducted by dedicated trial staff who will feel comfortable and trained in trial prosses and procedures. This will reduce the burden of the trial on clinical staff and removes barriers such as time constraints. Staff in the relevant departments will be briefed about the trial so they are aware that the trial is taking place.

Midwives and obstetricians local to Zomba Central Hospital have been consulted in the writing of this protocol to ensure it is acceptable and practical for local procedures.

Recruitment feasibility has been taken into account during the writing of this protocol. Recent audit data (August 2020 – January 2021) in Zomba Central Hospital showed that there were an average of 138 births a week, which is equal to an average of 19 births per day. Rates of caesarean section ranged from 19% to 52%. This indicates that recruitment will be feasible in around 6 to 9 months. The pragmatic study design has been formulated to ensure that there is minimal interference with routine clinical care.

Demand on participating women in labour has been kept to a minimum as antiseptic application will occur during routine VE's. For the newborn's in the neonatal strata, the antiseptic application and trial related assessments will be timed to not impact routine care and feeding. Antiseptic application and trial procedures will stop at discharge and participation in the trial will not impact duration of inpatient stay. We anticipate low dropout rates as only the day 28 trial visit is scheduled to take place after discharge; this follow up will occur over the phone or in person at the hospital (reimbursement will be paid for this in person visit).

The IMP is being procured centrally and will be available in sufficient supply prior to study commencement.

10. Funding and Justification

	FEC	Funded amount (GBP)	Funded amount (MKW)
Malawi-Liverpool-Wellcome Trust:			
DIRECTLY INCURRED COSTS			
Staff	£ 38,977.77	£ 38,977.77	39,407,152.13 MWK
Lab Consumables (including storage of samples)	£ 22,308.53	£ 22,308.53	22,554,282.49 MWK
Clinic Consumables (including stationary, clinic expenses and shipping costs)	£ 3,726.00	£ 3,726.00	3,767,045.90 MWK
IMP	£ 500.00	£ 500.00	505,508.04 MWK
Staff Miscellaneous	£ 1,300.00	£ 1,300.00	1,314,320.90 MWK
Research Support	£ 15,400.00	£ 15,400.00	15569647.59 MWK
Patient reimbursement on day 28 FU	£240	£240	242643.86 MWK
Ethics and Regulation Fees	£ 10,860.00	£ 10,860.00	10,979,634.60 MWK
INDIRECT COSTS	£ 6,772.00	£ 6,772.00	6,846,600.90 MWK
**Sub – Total**	**£ 100,084.30**	**£ 100,084.30**	**101,186,836.40 MWK**
**TOTAL funded by MRC**	**£ 112,472.41**	**£ 109,995.40**	**111,207,117.85 MWK**

Converted using https://ec.europa.eu/info/funding-tenders/how-eu-funding-works/information-contractors-and-beneficiaries/exchange-rate-inforeuro_en

Rates as of December 2020 £1= 1011.01608MWK

Cost	Justification
Staff –directly incurred Posts	One and a half midwives in Malawi will be necessary to consent and recruit women and to administer the intervention to women and to infants. The study will recruit over 6 months. We will employ midwives for 9 months to allow for training and for any necessary follow up following

	study completion, (£14.6K). The laboratory work for this study will all be done in Malawi. The primary end-point for this study is microbiological in nature and this requires intensive microbiology work. A full-time laboratory technician will be employed for 9 months to undertake this work, (£10.5K).This study is a clinical trial of two intervention agents which will need preparation as investigational medicinal products (IMP). This will require support from clinical trials pharmacy and this study will contribute 15% FTE of a pharmacist to support this, (£5K).The majority of the data management will occur at the Malawi Liverpool Wellcome Trust and data management time is included in the directly incurred costs from Malawi. Data management and project management at St George's, University of London (SGUL) will be given in-kind.
Staff –directly allocated	This is a pilot study with limited resources, and so directly allocated costs have been minimised with a substantial amount of work being given to this study in-kind. Dedicated time has been allocated for the Principle Investigator and local study Co-Investigators to provide oversite to the study. Specifically Professor Sharland (0.01FTE) will provide overall oversight for the study. Dr Emily Beales will co-ordinate the study. Professor Sarah Walker from MRC-Clinical Trials Unit at University College London (MRC-CTU/UCL) will give her contribution in-kind. To deliver the statistical analysis required by this study 2.5% FTE salary contribution will be made to Michelle Clements, a statistician at the MRC-CTU/UCL. She will provide support with analysis and interpretation of the results. Professor Feasey (0.026FTE) from Liverpool School of Tropical Medicine (LSTM) will support the microbiology within Malawi. Professor Lissauer (0.01FTE) from University of Liverpool will provide obstetric expertise. This will be supported by Dr Gadama (0.02FTE)in Malawi. Dr Dube (0.05FTE) will provide neonatal and paediatric oversight in Malawi. Dr Kumwenda (0.05FTE) in Malawi will lead the qualitative components, (£8.3K).
Other directly incurred costs	Consumables will be required to recruit participants, prepare the interventional product, administer interventional agents, collect swabs and to conduct the microbiological analysis required. These include paper and stationary, containers, aprons, sterile gloves, swabs, agar plates and media and inoculation loops. Chlorhexidine will be procured in-country. Octenidine Phenoxyethanol will be supplied from the manufacturer. Shipping for consumables has been included in the budget. Research consumables are estimated at £26,000. This study will be managed as a clinical trial and costs for the appropriate approvals and data management are incorporated (approximately £28,300). Patient reimbursement for day 28 follow up visit will be provided as a flat rate, £1.20 per patient and £240 in total.
Estates and indirect costs	The estate and indirect costs have been included for staff at SGUL, LSTM, University of Liverpool and MRC-CTU/UCL, (£4K). The indirect and overhead costs have also been included at 10% for University of Malawi, College of Medicine, (£6.7K).

DocuSign Envelope ID: B928C439-2A10-4CA0-932F-B711DAAFBA4E

11. References

1. Seale AC, Blencowe H, Manu AA, Nair H, Bahl R, Qazi SA, et al. Estimates of possible severe bacterial infection in neonates in sub-Saharan Africa, south Asia, and Latin America for 2012: a systematic review and meta-analysis. Lancet Infect Dis [Internet]. 2014 Aug 1 [cited 2019 May 15];14(8):731–41. Available from: https://www.sciencedirect.com/science/article/pii/S1473309914708047?via%3Dihub
2. Viswanathan R, Singh AK, Mukherjee S, Mukherjee R, Das P, Basu S. Aetiology and antimicrobial resistance of neonatal sepsis at a tertiary care centre in eastern India: a 3 year study. Indian J Pediatr [Internet]. 2011 Apr 17 [cited 2019 May 15];78(4):409–12. Available from: http://link.springer.com/10.1007/s12098-010-0272-1
3. Morkel G, Bekker A, Marais BJ, Kirsten G, van Wyk J, Dramowski A. Bloodstream infections and antimicrobial resistance patterns in a South African neonatal intensive care unit. Paediatr Int Child Health [Internet]. 2014 May 6 [cited 2019 May 15];34(2):108–14. Available from: http://www.tandfonline.com/doi/full/10.1179/2046905513Y.0000000082
4. Laxminarayan R, Matsoso P, Pant S, Brower C, Røttingen J-A, Klugman K, et al. Access to effective antimicrobials: a worldwide challenge. Lancet (London, England) [Internet]. 2016 Jan 9 [cited 2019 Apr 12];387(10014):168–75. Available from: http://www.ncbi.nlm.nih.gov/pubmed/26603918
5. Investigators of the Delhi Neonatal Infection Study (DeNIS) collaboration. Characterisation and antimicrobial resistance of sepsis pathogens in neonates born in tertiary care centres in Delhi, India: a cohort study. Lancet Glob Heal [Internet]. 2016 Oct 1 [cited 2019 Apr 12];4(10):e752-60. Available from: http://www.ncbi.nlm.nih.gov/pubmed/27633433
6. Kayange N, Kamugisha E, Mwizamholya DL, Jeremiah S, Mshana SE. Predictors of positive blood culture and deaths among neonates with suspected neonatal sepsis in a tertiary hospital, Mwanza- Tanzania. BMC Pediatr [Internet]. 2010 Dec 4 [cited 2019 Apr 12];10(1):39. Available from: http://bmcpediatr.biomedcentral.com/articles/10.1186/1471-2431-10-39
7. Chaurasia S, Sivanandan S, Agarwal R, Ellis S, Sharland M, Sankar MJ. Neonatal sepsis in South Asia: huge burden and spiralling antimicrobial resistance. BMJ [Internet]. 2019 Jan 22 [cited 2019 May 18];364:k5314. Available from: http://www.ncbi.nlm.nih.gov/pubmed/30670451
8. Benitz WE, Gould JB, Druzin ML. Risk Factors for Early-onset Group B Streptococcal Sepsis: Estimation of Odds Ratios by Critical Literature Review. Pediatrics [Internet]. 1999 Jun 1 [cited 2019 May 17];103(6):e77–e77. Available from: https://pediatrics.aappublications.org/content/103/6/e77.long
9. Sarff LD, Mccracken GH, Schiffer MS, Glode MP, Robbins JB, O rskov I, et al. Epidemiology of Ecoli K1 in healthy and diseased newborns. Lancet [Internet]. 1975 May 17 [cited 2019 May 16];305(7916):1099–104. Available from: https://www.sciencedirect.com/science/article/pii/S0140673675924964
10. Gibbs RS, Schrag S, Schuchat A. Perinatal Infections Due to Group B Streptococci. Obstet Gynecol [Internet]. 2004 Nov [cited 2019 Aug 1];104(5, Part 1):1062–76. Available from: http://www.ncbi.nlm.nih.gov/pubmed/15516403
11. Friedman S, Shah V, Ohlsson A, Matlow AG. Neonatal escherichia coli infections: concerns regarding resistance to current therapy. Acta Paediatr [Internet]. 2000 Jun [cited 2019 Aug 1];89(6):686–9. Available from:

DocuSign Envelope ID: B928C439-2A10-4CA0-932F-B711DAAFBA4E

http://www.ncbi.nlm.nih.gov/pubmed/10914963
12. Bulabula ANH, Dramowski A, Mehtar S. Maternal colonization or infection with extended-spectrum beta-lactamase-producing Enterobacteriaceae in Africa: A systematic review and meta-analysis. Int J Infect Dis [Internet]. 2017 Nov 1 [cited 2019 May 16];64:58–66. Available from: http://www.ncbi.nlm.nih.gov/pubmed/28890179
13. Karanika S, Karantanos T, Arvantitis M, Grigoras C, Mylonakis E. Fecal Colonization With Extended-spectrum Betalactamase–Producing Enterobacteriaceae and Risk Factors Among Healthy Individuals: A Systematic Review and Meta-analysis. Clin Infect Dis [Internet]. 2016 [cited 2019 May 16];63(3):310–8. Available from: https://watermark.silverchair.com/ciw283.pdf?token=AQECAHi208BE49Ooan9kkhW_Ercy7Dm3ZL_9Cf3qfKAc485ysgAAAjcwggIzBgkqhkiG9w0BBwagggIkMIICIAIBADCCAhkGCSqGSIb3DQEHATAeBglghkgBZQMEAS4wEQQM6FZAeHyzaOqOP1-NAgEQgIIB6vvMX8F7IHcQ2cgO4MfntXH3YqPdwHH2q8Cm7YKwdxsEVcTl
14. Johnson J, Suwantarat N, Colantuoni E, Ross TL, Aucott SW, Carroll KC, et al. The impact of chlorhexidine gluconate bathing on skin bacterial burden of neonates admitted to the Neonatal Intensive Care Unit. J Perinatol [Internet]. 2019 Jan 20 [cited 2019 May 19];39(1):63–71. Available from: http://www.nature.com/articles/s41372-018-0231-7
15. Darmstadt GL, Hossain MM, Choi Y, Shirin M, Mullany LC, Islam M, et al. Safety and effect of chlorhexidine skin cleansing on skin flora of neonates in Bangladesh. Pediatr Infect Dis J [Internet]. 2007 Jun [cited 2019 May 19];26(6):492–5. Available from: https://insights.ovid.com/crossref?an=00006454-200706000-00008
16. Simon A, Exner M. Prävention nosokomialer Infektionen bei intensivmedizinisch behandelten Frühgeborenen. Monatsschrift Kinderheilkd [Internet]. 2014;162(5):403–10. Available from: https://doi.org/10.1007/s00112-013-2974-8
17. Biermann C, Kribs A, Roth B, Tantcheva-Poor I. Use and Cutaneous Side Effects of Skin Antiseptics in Extremely Low Birth Weight Infants – A Retrospective Survey of the German NICUs. Klin Pädiatrie [Internet]. 2016 Jun 30 [cited 2019 May 16];228(04):208–12. Available from: http://www.ncbi.nlm.nih.gov/pubmed/27362412
18. Novakov Mikić A, Stojic S. Study results on the use of different therapies for the treatment of vaginitis in hospitalised pregnant women. Arch Gynecol Obstet [Internet]. 2015 Aug 5 [cited 2019 May 16];292(2):371–6. Available from: http://www.ncbi.nlm.nih.gov/pubmed/25651828
19. Alvarez-Marin R, Aires-de-Sousa M, Nordmann P, Kieffer N, Poirel L. Antimicrobial activity of octenidine against multidrug-resistant Gram-negative pathogens. Eur J Clin Microbiol Infect Dis [Internet]. 2017 Dec 19 [cited 2019 May 16];36(12):2379–83. Available from: http://link.springer.com/10.1007/s10096-017-3070-0
20. Messler S, Klare I, Wappler F, Werner G, Ligges U, Sakka SG, et al. Reduction of nosocomial bloodstream infections and nosocomial vancomycin-resistant Enterococcus faecium on an intensive care unit after introduction of antiseptic octenidine-based bathing. J Hosp Infect [Internet]. 2019 Mar [cited 2019 May 16];101(3):264–71. Available from: http://www.ncbi.nlm.nih.gov/pubmed/30408504
21. Cutland CL, Madhi SA, Zell ER, Kuwanda L, Laque M, Groome M, et al. Chlorhexidine maternal-vaginal and neonate body wipes in sepsis and vertical transmission of pathogenic bacteria in South Africa: a randomised, controlled trial. Lancet (London, England) [Internet]. 2009 Dec 5 [cited 2019 May 16];374(9705):1909–16. Available from: http://www.ncbi.nlm.nih.gov/pubmed/19846212
22. Mullany LC, Biggar RJ. Vaginal and neonatal skin cleansing with chlorhexidine. Lancet

DocuSign Envelope ID: B928C439-2A10-4CA0-932F-B711DAAFBA4E

(London, England) [Internet]. 2009 Dec 5 [cited 2019 May 29];374(9705):1873–5. Available from: http://www.ncbi.nlm.nih.gov/pubmed/19846213
23. Taha T, Biggar R, Broadhead R, al. et. Effect of cleansing the birth canal with antiseptic solution on maternal and newborn morbidity and mortality in Malawi: clinical trial. BMJ. 1997;315:216–9.
24. Bakr A, Karkour T. Effect of predelivery vaginal antisepsis on maternal and neonatal morbidity and mortality in Egypt. J Womens Heal. 2005;14:496–501.
25. Bell C, Hughes L, Akister T, Ramkhelawon V, Wilson A, Lissauer D. What is the result of vaginal cleansing with chlorhexidine during labour on maternal and neonatal infections? A systematic review of randomised trials with meta-analysis. BMC Pregnancy Childbirth [Internet]. 2018 Dec 8 [cited 2019 Jun 6];18(1):139. Available from: https://bmcpregnancychildbirth.biomedcentral.com/articles/10.1186/s12884-018-1754-9
26. Ohlsson A, Shah VS, Stade BC. Vaginal chlorhexidine during labour to prevent early-onset neonatal group B streptococcal infection. Cochrane Database Syst Rev [Internet]. 2014 Dec 14 [cited 2019 May 16];(12). Available from: http://doi.wiley.com/10.1002/14651858.CD003520.pub3
27. UNITED NATIONS. “Sustainable Development Goal 3: Ensure healthy lives and promote well-being for all at all ages,” Sustainable Development Goals Knowledge Platform, 2017. In.
28. Taylor AW, Blau DM, Bassat Q, Onyango D, Kotloff KL, Arifeen S El, et al. Initial findings from a novel population-based child mortality surveillance approach: a descriptive study. Lancet Glob Heal. 2020;8(7):e909–19.
29. Tielsch JM, Darmstadt GL, Mullany LC, Khatry SK, Katz J, LeClerq SC, et al. Impact of Newborn Skin-Cleansing with Chlorhexidine on Neonatal Mortality in Southern Nepal: A Community-Based, Cluster-Randomized Trial. Pediatrics. 2007;119(2):e330–40.
30. Johnson J, Suwantarat N, Colantuoni E, Ross TL, Aucott SW, Carroll KC, et al. The impact of chlorhexidine gluconate bathing on skin bacterial burden of neonates admitted to the Neonatal Intensive Care Unit. J Perinatol. 2019;39(1):63–71.
31. World Health Organisation. World Health Organization Model List of Essential Medicines for Children 7th List (2019). Report [Internet]. 2019;(June):1–42. Available from: https://www.who.int/medicines/publications/essentialmedicines/en/
32. Vorherr H, Vorherr U., Mehta P, J.A U, Messer R. Antimicrobial effect of chlorhexidine and povidone-iodine on vaginal bacteria. J Infect [Internet]. 1984;8:195–9. Available from: http://www.scopus.com/inward/record.url?eid=2-s2.0-0141828938&partnerID=40&md5=cff90dc9726b831c7cdced69636355d1
33. Pereira L, Chipato T, Mashu A, Mushangwe V, Rusakaniko S, Bangdiwala SI, et al. Randomized study of vaginal and neonatal cleansing with 1% chlorhexidine. Int J Gynecol Obstet. 2011;112(3):234–8.
34. Shippey SH, Malan TK. Desquamating vaginal mucosa from chlorhexidine gluconate. Obstet Gynecol. 2004;103(5 Pt 2):1048–50.
35. Anderson MJ, Scholz MT, Parks PJ, Peterson ML. *Ex vivo* porcine vaginal mucosal model of infection for determining effectiveness and toxicity of antiseptics. J Appl Microbiol [Internet]. 2013 Sep [cited 2019 Jun 7];115(3):679–88. Available from: http://www.ncbi.nlm.nih.gov/pubmed/23773892
36. Linke G, Tarantino I, Bruderer T, Celeiro J, Warschkow R, Tarr P, et al. Transvaginal access for NOTES: a cohort study of microbiological colonization and contamination. Endoscopy [Internet]. 2012 Jul 23 [cited 2019 Jun 6];44(07):684–9. Available from:

DocuSign Envelope ID: B928C439-2A10-4CA0-932F-B711DAAFBA4E

http://www.ncbi.nlm.nih.gov/pubmed/22528675
37. Briese V, Neumann G, Waldschläger J, May TW, Siebert J, Gerber B. Efficacy and tolerability of a local acting antiseptic agent in the treatment of vaginal dysbiosis during pregnancy. Arch Gynecol Obstet [Internet]. 2011 Mar 9 [cited 2019 Jun 6];283(3):585–90. Available from: http://www.ncbi.nlm.nih.gov/pubmed/20217109
38. Mikic A, Budakov D. Comparison of local metronidazole and a local antiseptic in the treatment of bacterial vaginosis. Arch Gynecol Obstet. 2010;(282):43–7.
39. WHO recommendation on digital vaginal examination | RHL [Internet]. [cited 2019 Oct 13]. Available from: https://extranet.who.int/rhl/topics/preconception-pregnancy-childbirth-and-postpartum-care/care-during-childbirth/care-during-labour-1st-stage/who-recommendation-digital-vaginal-examination
40. Sathiyamurthy S, Banerjee J, Godambe S V. Antiseptic use in the neonatal intensive care unit - a dilemma in clinical practice: An evidence based review. World J Clin Pediatr [Internet]. 2016 [cited 2019 Jul 15];5(2):159. Available from: http://www.wjgnet.com/2219-2808/full/v5/i2/159.htm
41. O'Brien CA, Blumer J., Speck W., Carr H. Effect of bathing with a 4 per cent chlorhexidine gluconate solution on neonatal bacterial colonization [Internet]. Journal of Hospital Infection. 1984 [cited 2019 Aug 9]. Available from: https://www.journalofhospitalinfection.com/article/0195-6701(84)90069-0/pdf
42. Mullany LC, Darmstadt GL, Tielsch JM. Safety and impact of chlorhexidine antisepsis interventions for improving neonatal health in developing countries. Pediatr Infect Dis J [Internet]. 2006 Aug [cited 2019 Jun 6];25(8):665–75. Available from: http://www.ncbi.nlm.nih.gov/pubmed/16874163
43. Wilson CM, Gray G, Read JS, Mwatha A, Lala S, Johnson S, et al. Tolerance and safety of different concentrations of chlorhexidine for peripartum vaginal and infant washes: HIVNET 025. J Acquir Immune Defic Syndr [Internet]. 2004 Feb 1 [cited 2019 Aug 9];35(2):138–43. Available from: http://www.ncbi.nlm.nih.gov/pubmed/14722445
44. Garland J, Alex C, Mueller C, Cisler-Kahill L. Local reactions to a chlorhexidine gluconate-impregnated antimicrobial dressing in very low birth weight infants. Pediatr Infect Dis J. 1996 Dec 20;15(10):912–4.
45. Bührer C, Bahr S, Siebert J, Wettstein R, Geffers C, Obladen M. Use of 2% 2-phenoxyethanol and 0.1% octenidine as antiseptic in premature newborn infants of 23–26 weeks gestation. J Hosp Infect [Internet]. 2002 Aug [cited 2019 Jul 15];51(4):305–7. Available from: https://linkinghub.elsevier.com/retrieve/pii/S0195670102912491
46. Darmstadt GL, Badrawi N, Law PA, Ahmed S, Bashir M, Iskander I, et al. Topically Applied Sunflower Seed Oil Prevents Invasive Bacterial Infections in Preterm Infants in Egypt. Pediatr Infect Dis J [Internet]. 2004 Aug [cited 2019 Aug 11];23(8):719–25. Available from: https://insights.ovid.com/crossref?an=00006454-200408000-00005
47. Lund CH, Osborne JW. Validity and reliability of the neonatal skin condition score. J Obstet Gynecol neonatal Nurs JOGNN [Internet]. [cited 2019 Aug 11];33(3):320–7. Available from: http://www.ncbi.nlm.nih.gov/pubmed/15180195
48. IMPAACT P1106: Pharmacokinetic Characteristics of Antiretrovirals and Tuberculosis Medicines in Low Birth Weight Infants - Full Text View - ClinicalTrials.gov.
49. Gordon SB, Chinula L, Chilima B, Mwapasa V, Dadabhai S, Mlombe Y. A Malawi guideline for research study participant remuneration. Wellcome Open Res. 2018;3(0).

DocuSign Envelope ID: F49E6E37-298A-446A-A83D-BE73F1829E60

NeoVT-AMR

Strategies to reduce vertical transmission of multi-drug resistant pathogens to neonates (NeoVT-AMR)

NeoVT-AMR Statistical Analysis Plan			
Version Number and Date: v1.0 2023-05-10<br>Supersedes version: 0.8<br>Protocol version 6.0			
**Author**	**Position**	**Signature**	**Date**
Michelle Clements	Trial statistician	DocuSigned by: Michelle Clements AD78954D2C79466...	2023-May-10
**Approved by**			
Sarah Walker	Co-investigator & statistical reviewer	DocuSigned by: Ann Sarah Walker A21A8226533E49A...	2023-May-10
James Carpenter	Independent reviewer	DocuSigned by: James Carpenter D39C318C5788411...	2023-May-10

Revision History

Version	Author	Date	Reason for Revision
Draft 0.1	MC	02/02/2022	First draft – copy of protocol version 3.0
Draft 0.2	MC	12/08/2022	First substantial draft
Draft 0.3	MC	12/09/2022	Updated after comments from SW
Draft 0.4	MC	21/09/2022	Updated with subgroups following discussion in TMG
Draft 0.5	MC	04/10/2022	Updated following comments from James Carpenter
Draft 0.6	MC	22/02/2023	Updated following comments from James Carpenter and Andrew Atkinson and following interim analyses of safety data (see interim analysis section below)
Draft 0.7	MC	13/04/2023	Updated with prior information for Bayesian analysis
Draft 0.8	MC	24/04/2023	Updated after comments by EB
V1.0	MC	10/05/2023	Updated after final comments by JC and upversioned

Contents

1. Trial design 4
1.1. Design & outline 4
1.2. Population 5
2. Outcome Measures 6
2.1. Co-Primary Outcome Measures 6
2.2. Secondary Outcome Measures 6
2.3. Additional secondary efficacy outcomes not in protocol 6
2.4. Sample Size Calculation 7
2.5. Method of Randomisation 7
2.6. Estimands 7
2.7. COVID-19 8
2.8. Interim analyses 8
2.9. Final analyses 8
2.10. Software 9
3. Derivation of data to be analysed 10
3.1. Definition of baseline 10
3.2. Follow-up timings 10
3.3. Loss to follow up 10
3.4. Free text 10
4. Statistical Analyses 11
4.1. Recruitment & Randomisation 11
4.2. Baseline 11
4.3. Non-Trial Treatment 12
4.4. Trial Treatment 12
4.5. Follow-up 13
4.6. Primary outcome analyses 14
4.7. Secondary efficacy outcome analyses 16
4.8. Secondary safety outcome analyses: skin score 16
4.9. Secondary safety outcome analyses: temperature 17
4.10. Secondary safety outcome analyses: AEs and SAEs 18
4.11. Additional secondary efficacy outcomes analysis 18
5. References 19
6. Appendix: Bayesian priors 20

DocuSign Envelope ID: F49E6E37-298A-446A-A83D-BE73F1829E60

6.1. Non-informative priors 20
6.2. Informative priors (optimistic and sceptical) 20

1. Trial design

1.1. Design & outline

NeoVT-AMR is a randomised controlled factorial trial with 3x2 + standard of care (SOC) design with two independent strata – labouring mothers and hospitalised neonates. Within each stratum, 147 individuals will be recruited – 21 per treatment combination or SOC. Additionally, 147 babies from mothers in the maternal stratum will also be swabbed at birth.

The two factors are:

- Antiseptic: 1% chlorhexidine (CHG), 2% CHG and octenisept (OCT)
- Frequency of application: once only and multiple applications

Multiple applications timings are 0/4/8/24/28/32h in maternal strata and 0/1/2/3d in neonatal strata.

The SOC group will receive standard of care. For analyses, the reference arm will be 1% CHG once only as there are more effective replicates of each of the factorial arms and therefore greater power for comparison than with SOC. 1% CHG once only was selected as it would be the most straightforward to implement and CHG is more widely available than OCT so evidence of superiority of OCT over CHG would have to be shown for implementation to be advised.

The trial design is summarised in the trial scheme below. Note that the 'once only' frequency boxes are (n=21) also.

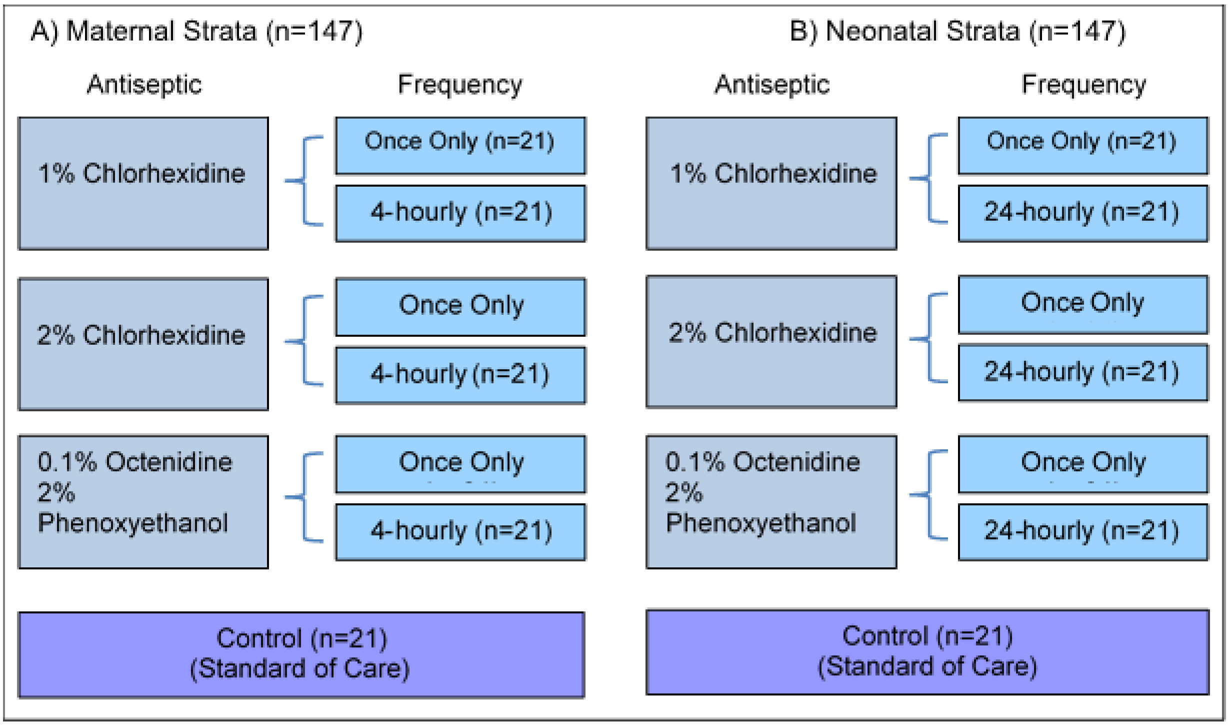

1.2. Population

Eligibility to the trial is based on the participant meeting all of the inclusion criteria and none of the exclusion criteria.

Inclusion criteria

Maternal strata

1. Presenting in labour with or without rupture of membranes

Neonatal strata

1. Born in a healthcare facility
2. Postnatal age at randomisation <24 hours
3. Birth weight >1000g

Exclusion criteria

Maternal strata

1. Under the age of 18 (minor in Malawi)
2. Any contra-indication to digital vaginal examination
3. In active labour (regular contractions and/or cervical dilatation of more than 4cm)
4. Poor perineal and vaginal skin condition as judged by clinician
5. Planned elective caesarean-section delivery
6. Known or suspected allergy to chlorhexidine or octenidine or phenoxyethanol
7. Intrauterine death confirmed or expected before randomisation
8. Antiseptic application or enrolment in the trial determined inappropriate in the opinion of the enrolling clinician
9. Any recent or planned (within 4 hours) iodine application to the perineum or vagina
10. Unable to obtain consent

Neonatal strata

1. Born by planned elective caesarean section
2. Born to mothers recruited in the trial
3. Poor skin condition (skin score of 2 or more in any of three domains (see <u>Appendix I</u>)) at the time of enrolment
4. Known congenital or acquired skin disorder or defect at time of enrolment
5. Antiseptic application or enrolment in the trial determined inappropriate in the opinion of the enrolling clinician
6. Any recent or planned (within 4 hours) iodine application to body
7. Any planned or previous lumbar puncture
8. Unable to obtain parental or guardian consent

The primary analysis population is intention-to-treat, including all randomised babies, regardless of treatment received. This corresponds to estimating the impact of the effectiveness of the treatments. However, in secondary analyses we will also use inverse-probability weighting methods to adjust for deviation from randomised strategy if non-compliance rates are >15%, which is a more efficient approach than defining a per-protocol population (1).

Loss-to-follow rates are unknown but may be high as participants may give birth (in the maternal strata) or be discharged (in the neonatal strata) before all planned swabs are taken. Missing swabs

will be monitored and participants without baseline and two post-baseline swabs may be replaced to maintain power. However, all baseline and post-baseline results will be included in analyses.

2. Outcome Measures

2.1. Co-Primary Outcome Measures

Maternal strata

- Change in vaginal and perineal bacterial load (in colony forming units)

Neonatal strata

- Change in neonatal skin bacterial load (in colony forming units)

Bacterial load will be assessed from vaginal and perineal swabs taken separately in the maternal strata and from neck and perirectal swabs taken separately in the neonatal strata. The log CFU for each swab will be summed to get the total log CFU. Timings of the swabs are the same as the timings for applications in the multiple application arm. Swabs are taken before antiseptic is applied.

2.2. Secondary Outcome Measures

Maternal strata

- Maternal toxicity – score and grade (tolerability and safety)
- Bacterial load in neonates exposed to maternal antiseptic, compared to control
- Serious adverse events

Neonatal strata

- Adapted neonatal skin condition score (safety) (absolute score and grade)
- Temperature (change in absolute temperature and grade (hypothermia))
- Serious adverse events

2.3. Additional secondary efficacy outcomes not in protocol

Maternal strata

- Change in vaginal and perineal bacterial load (in log CFU) of specific species:
  - All gram positive
  - All gram negative
  - Yeast
  - All enterobactereales combined (E. coli, Enterobacter, klebsiella, Proteus-Providencia-Morganella, Serratia, other enterobactereales)
  - Klebsiella spp.
  - E. coli
  - Serratia spp.
  - Proteus-Providencia-Morganella
  - Enterobacter spp.
  - Acinetobacter spp.
  - Staphylococcus aureus
  - Group A streptococcus (GAS)
  - Group B streptococcus (GBS)
  - GAS and GBS combined
  - Enterococcus spp.
  - Candida spp.
  - Pseudomonas spp.
  - Salmonella spp.

DocuSign Envelope ID: F49E6E37-298A-446A-A83D-BE73F1829E60

- Other Enterobacterales
- Acquisition and loss of specific species above
- Components of total CFU considered separately
- Components of skin scores considered separately

Neonatal strata

- Change in neonatal skin bacterial load (in log CFU) of specific species as per maternal strata
- Acquisition and loss of specific species as per maternal strata
- Components of total CFU considered separately
- Components of skin scores considered separately

2.4. Sample Size Calculation

There is very little data on bacterial load in neonates in LMICs. Additionally, bacterial loads are expected to differ across locations complicating interpretation of previous studies. For example, previous studies in neonates have found varying effects of CHG on log colony counts: a decrease from baseline to 24h of 0.2 SD with 1% CHG in Nepal (2) and 2SDs with 2% CHG in the USA (3). The primary endpoints of log colony forming units (CFUs) are widely accepted to be normally distributed. Sample size calculations used standard formula with Bonferroni adjustment for multiple testing within arms of frequency and antiseptic. Within each stratum, 147 individuals randomized to 7 treatment groups, 21 participants per group, provides 90% power to detect a difference of 0.82 standard deviations (SDs) between antiseptics (two-sided $\alpha=0.012$) and 0.58 SDs between frequencies ($\alpha=0.05$) (80% power for 0.72 and 0.50 SDs, respectively). This also provides 90% power to detect a difference of 1.01 SD between each antiseptic and SOC ($\alpha=0.012$), and 0.90 SD between frequency and SOC ($\alpha=0.025$) (0.88 and 0.79 SDs at 80% power). These expected effects were judged by clinical and statistical members of the team to be sufficient for selecting a treatment regimen for a future trial.

2.5. Method of Randomisation

Randomisation is be performed using a random number generated as implemented in the statistical computing environment R. Randomisation is by permuted blocks (random block sizes of 7 and 14 randomly selected) to guard against bias introduced over time, such as outbreaks of pathogenic bacteria in the hospital. Participants are randomised 1:1:1:1:1:1:1 to the 6 treatment and 1 SOC arms.

Randomisation is built into electronic data systems using algorithms developed by the data management team at the Malawi Liverpool Wellcome Trust (MLW). The participant's trial number, treatment allocation and the date of randomisation is entered into the trial register at the site.

2.6. Estimands

The intervention is the randomised antiseptic and frequency of application.

The patient population is labouring mothers and new-born babies, with birth weight over 1kg admitted to hospital, as defined by the inclusion and exclusion criteria.

The co-primary endpoints are change in skin bacterial colony load, taken from vaginal and perineal swabs in the maternal strata and neck and perirectal swabs in the neonatal strata.

DocuSign Envelope ID: F49E6E37-298A-446A-A83D-BE73F1829E60

The population-level summary which provides a basis for comparison between treatment conditions is the difference in mean log colony forming units adjusted for baseline value.

Anticipated intercurrent events and associated strategy are:

- Loss to follow-up: rates are unknown but may be high as not many women are anticipated to labour for 32h. The mixed model gives valid estimates assuming outcomes are missing at random.
- Deviation from randomised strategy (antiseptic application and frequency as per protocol) will be assessed using multiple sensitivity analyses:
    - sensitivity analysis will censor at deviation from randomised strategy. Estimated treatment effects will then correspond to the (hypothetical) setting of no deviation.
    - sensitivity analysis using (a) post-intercurrent event data where appropriate and (b) MI will be used to assess robustness of treatment effect inferences where rates of deviation from protocol are above 15%.
    - sensitivity analysis using Inverse-probability weighting methods to adjust for deviation from randomised strategy if deviation rates are above 15%.

2.7. COVID-19

Eligible patients will be mothers and neonates within the hospital so risk of COVID-19 will be low. NeoVT-AMR was designed before the COVID-19 pandemic and so there are no explicit statistical mitigation strategies. Rate of positive COVID tests in trial participants and any resulting changes to treatment will be monitored and addressed in the analysis if necessary.

2.8. Interim analyses

The DMC met around the time the trial started (without reviewing any data) to ensure they were familiar with its design and conduct. They formally reviewed data halfway through planned recruitment. They may call additional meetings at any time at their discretion. In addition, SARs are sent in real time during the trial to the DMC. The DMC can recommend premature closure or reporting of the trial, or that recruitment to any randomised group be discontinued or modified. Such recommendations would be made if, in the view of the DMC, there is an unacceptable rate of adverse reactions. There will be no early stopping for clinical efficacy because this pilot trial is powered only to detect differences in skin bacterial load, not clinical efficacy outcomes.

The review halfway thorough recruitment was held on 8th November 2022 and reviewed safety data but not efficacy, as per the protocol. The SAP was not signed off at this point as there were still some unresolved questions from the statistical and DMC reviewer around the analyses of the efficacy endpoints. It was agreed with the DMC that it was preferable to go ahead with an incomplete SAP than delay safety analyses. Note that there were no issues around analyses of the safety endpoints considered during the DMC. V0.5 was the version in use during the DMC review. A SOP deviation was completed for this.

There will be no adjustment to the significance level due to interim analysis.

2.9. Final analyses

Final analysis will be after database lock following last-patient last-visit.

2.10. Software

Primary and secondary outcome analyses will be done in Stata v17 or later. Data preparation and analyses of other data will be done in R v4.2.1 or later.

3. Derivation of data to be analysed

3.1. Definition of baseline

Baseline values for all measurements will be those recorded at screening either on the screening and enrolment form, or zero hour treatment and microbiology data from the treatment and microbiology forms.

3.2. Follow-up timings

Timings of swabs will be:

Maternal strata

- 0h (baseline)
- 4h
- 8h
- 24h
- 28h
- 32h

Neonatal strata

- 0 day (baseline)
- 1 day
- 2 day
- 3 day

3.3. Loss to follow up

Loss-to-follow up rates are unknown but may be high. Missing swabs will be monitored and participants may be replaced to maintain power. However, all baseline and post-baseline results will be included in analyses.

3.4. Free text

Free text fields in CRFs may be corrected for spelling and further categorised.

4. Statistical Analyses

Information will be presented in tables and may also be presented graphically to aid interpretation.

Recruitment data will be presented as per standard CONSORT diagrams (4). Baseline data tables will be presented overall. Unless stated, post-baseline data tables will be presented aggregated by antiseptic and by frequency (working days, alternate working days), with SOC presented separately. Variables will also be presented by factorial randomisation or arm if there is difference between randomised groups of $p<0.05$, used as a flagging device for imbalance and expected for 1 in 20 characteristics by chance, with p-values from t-tests of differences between means for numeric variables and chi-squared tests or Fisher's exact test if cell values are small for categorical variables.

Statistical tests will use 95% confidence intervals unless otherwise stated. Associated two-sided p-values will be produced but binary conclusions of significant/not significant will not be drawn. As this is a pilot trial there will be no adjustment for multiple testing, although interpretation of results will take this into consideration.

All analyses will be included in the interim and final reports unless stated. All analyses will be presented by strata separately and not combined.

IQR: interquartile range

4.1. Recruitment & Randomisation

The following metrics will be presented by strata:

- Total randomisation, n(%)
- Randomisation to each antiseptic concentration: n(%)
- Randomisation to each application frequency: n(%)
- Randomisation to each antiseptic/freq combination: n(%)
- Eligibility: number and reasons for any participants randomised in error and excluded or ineligible children included in the analysis

4.2. Baseline

Maternal

- Age at randomisation (years): median (IQR)
- Estimated gestational age at labour: median (IQR)
- Gravidity: median (IQR)
- Parity: median (IQR)
- Antenatal care during pregnancy: n(%)
- Tuberculosis: n(%)
- HIV: n(%) positive, negative, unknown
- Gestational diabetes: n(%)
- Antibiotics during pregnancy: n(%)
- Antibiotics since admission: n(%)
- Cervical dilation at enrolment: median (IQR)
- Rupture of membranes at enrolment: n(%) yes, no

DocuSign Envelope ID: F49E6E37-298A-446A-A83D-BE73F1829E60

- If ruptured, how ruptured: n(%) spontaneous, assisted
- Prolonged rupture of membranes (>18h): n(%) yes, no, unknown
- Offensive liquor: n(%) yes, no, unknown

Neonatal

- Prolonged rupture of membranes (>18h): n(%) yes, no, unknown
- Offensive liquor: n(%) yes, no, unknown
- Mother received antibiotics in labour: n(%) yes, no, unknown
- Mode of delivery: n(%) vaginal spontaneous, vaginal assisted, emergency caesarean section
- Maternal HIV status: n(%) positive, negative, unknown
- Age at enrolment (hours): median (IQR)
- Estimated gestational age at labour: median (IQR)
- Sex: n(%) male, female
- AGPAR Score at 1 minute: median (IQR)
- AGPAR Score at 5 minutes: median (IQR)
- AGPAR Score at 10 minutes: median (IQR)
- Where admitted: n(%) postnatal ward with mother, nursery HDU, nursery
- Reason for admission: n(%) routine for mum and baby, maternal complication – baby admitted with mum, birth asphyxia, other
- Any antibiotics prescribed before enrolment since birth: n(%)
- Chlorhexidine cord care given: n(%)

4.3. Non-Trial Treatment

Maternal and neonatal strata

- Antibiotics received post-baseline: n(%)
- Other conmeds received post-baseline: n(%)
- Length of hospital stay: median (IQR)

4.4. Trial Treatment

The following metrics will be presented by factor and strata. Note that compliance to antiseptic will not be assessed as patients have individual dose bottles for application.

Note that swab timings are 0/4/8/24/28/32h in maternal strata and 0/1/2/3d in neonatal strata.

- Total number of antiseptic applications: median (IQR) and 1, 2… n(%)
- Timing of last antiseptic application: n(%) 0/4/8/24/28/32h in maternal strata and 0/1/2/3d in neonatal strata
- Number of antiseptic applications before each swab: median (IQR)
- Temperature taken before antiseptic application: n(%) in neonatal strata only
- Skin score assessed before antiseptic application: n(%)
- Temperature taken after antiseptic application: n(%) in neonatal strata only
- Skin score assessed after antiseptic application: n(%)

4.5. Follow-up

The following metrics will be presented by strata:

- Baseline swab: n(%) yes
- Number of swabs: median (IQR)
- Time in hours from baseline when each swab taken: median (IQR)
- Number of skin score assessments: median (IQR)
- Last time point of skin score assessment: : n(%) 0/4/8/24/28/32h in maternal strata and 0/1/2/3d in neonatal strata
- Day 28 follow-up: n(%)

4.6. Primary outcome analyses

Note that no efficacy outcome data will be presented at the interim analysis. Analyses beyond the primary analysis may not be presented at final analysis, depending on results from primary analysis.

A summary table will be presented:

- Total log10 CFU at baseline: mean (SD) [N]
- Total log10 CFU at each assessment time point: mean (SD) [N]
- Change in total log10 CFU from baseline to each assessment time point: mean (SD) [N]

Primary analysis

- The primary analysis population is intention-to-treat, including all randomised participants, regardless of treatment received.
- The outcome variable will be change in Total log10 CFU from baseline to assessment time point.
- The primary analysis will include all randomised participants with baseline and at least one post-baseline measure.
- The dependent variable will be total log CFU.
- The model will be a mixed effects model
- Fixed effects in the model will be:
  - Antiseptic (1% CHG, 2% CHG, OCT)
  - Frequency of application (once, multiple)
  - Time point of assessment: hour of swab (continuous; mfp will be used to assess linearity)
  - Baseline total CFU (continuous; mfp will be used to assess linearity)
- Individual will be fitted as a random effect to account for repeated measures within individuals. Goodness of fit of an unstructured covariance matrix will be assessed and used if it is clearly a better fit to the data than random intercepts only.
- Goodness of fit of an interaction between baseline total CFU and timepoint of assessment will be assessed and included in the model if clearly a better fit.
- Normally distributed errors will be fitted.
- The intercept (reference treatment category) will be once only 1% CHG as this is the lowest concentration of CHG, which is believed to be more readily available that OCT, and so would be the most straightforward to implement. The standard of care control will not be used as the reference as the sample size for this arm is lower than for the factorial arms and so power for comparisons is correspondingly lower.
- Comparison of effects between factorial arms will be assessed by testing whether the null hypothesis of no treatment effect can be rejected (equivalent to using the estimate and 95% confidence interval of the estimate of the difference to the intercept).
- Comparisons between other arms and between arms and the SOC arm may also be performed. There will be no formal correction for multiple testing but interpretation will take multiple testing into consideration.
- Model fitting will use restricted maximum likelihood (REML) and p-values are derived using the Kenward-Roger approximation.

DocuSign Envelope ID: F49E6E37-298A-446A-A83D-BE73F1829E60

Interaction analysis

- Interactions between antiseptic and frequency, antiseptic and timepoint of swab, and frequency and timepoint of swabs will also be fitted in separate models, to assess evidence for the presence of interactions.
- Models and reporting of results will be as per the primary analysis.

Repeated samples efficacy sensitivity analysis

- Change from baseline to the:
    - first outcome measure after baseline
    - final outcome measure

  will also be modelled
- Model and reporting of results will be as the primary analysis except individual will not be fitted as a random effect (as there will be no repeated measures within individuals)

Time since antiseptic efficacy sensitivity analysis

- The primary analysis described above will be repeated with time since last antiseptic application fitted as a fixed effect (continuous; mfp will be used to assess linearity)

IV antibiotics efficacy sensitivity analysis

- The primary efficacy analysis described above will be repeated with received IV antibiotics in last 24h/not fitted as a fixed effect (factor)

Deviation from randomised strategy sensitivity censoring analysis

- The primary efficacy analysis described above will be repeated with individuals censored at the point of deviation from randomised strategy if deviation rates are above 15%.

Deviation from randomised strategy multiple imputation analysis

- The primary efficacy analysis described above will be repeated using multiple imputation on individuals censored at the point of deviation from randomised strategy if deviation rates are above 15%.

Deviation from randomised strategy multiple IPW analysis

- The primary efficacy analysis described above will be repeated using IPW analysis on individuals censored at the point of deviation from randomised strategy if deviation rates are above 15%.

Bayesian analysis

- Primary outcome main analysis will also be conducted in a Bayesian framework using ACCEPT analyses (5) reporting the probability that one treatment arm is more effective than another treatment arm based on a continuous range of efficacy thresholds.
- Models will be fitted as the primary analyses above.
- Sensitivity analysis to prior assumptions will be performed using non-informative, optimistic and sceptical priors (see appendix for details). The analysis will focus on non-informative

DocuSign Envelope ID: F49E6E37-298A-446A-A83D-BE73F1829E60

priors, with informative priors used for sensitivity analysis, unless there are model fitting issues with non-informative priors.

- Posterior probability curves will be created for each factor compared to SOC and between arms within a factor.
- The posterior probability of each arm truly being better that the comparator will be calculated for each factor arm compared to SOC and between arms within a factor.

Subgroup analyses

Subgroup analyses will be:

Maternal

- HIV
- Antibiotics since admission
- Rupture of membranes at enrolment
- Prolonged rupture of membranes (>18h)
- Offensive liquor

Neonatal

- Maternal HIV status
- Mother received antibiotics in labour
- Prolonged rupture of membranes (>18h)
- Offensive liquor
- Age at enrolment
- Estimated gestational age at labour
- Where admitted
- Any antibiotics prescribed before enrolment since birth
- Chlorhexidine cord care given

The main effect will be fitted as above for the primary analysis with additional main effect of subgroup. The interaction between subgroup and each factor (antiseptic and frequency) will also be fitted one at a time, in separate models. Interpretation of results will take multiple testing and statistical power into consideration.

4.7. Secondary efficacy outcome analyses

Note that no efficacy outcome data will be presented at the interim analysis.

Analysis of bacterial load in neonates exposed to maternal antiseptic will be as per primary outcome analyses described above.

4.8. Secondary safety outcome analyses: skin score

A summary table will be presented:

- Skin score at baseline: mean (SD) [N]
- Skin score at each assessment time point: mean (SD) [N]
- Change in total log10 CFU from baseline to each assessment time point: mean (SD) [N]

DocuSign Envelope ID: F49E6E37-298A-446A-A83D-BE73F1829E60

- Absolute skin score at baseline: n(%) 0, 1, 2…
- Absolute skin score at each assessment time point: n(%) 0, 1, 2…

Skin score analysis

- Analyses will be as per the primary outcome above, except baseline skin score will be used in place of baseline log CFU.
- Transformations may be performed if there is clear evidence of deviations from normally distributed errors in the safety outcome. If more than 70% of the safety endpoint are in one category, goodness of fit of alternative models (e.g. ordinal) will be considered

4.9. Secondary safety outcome analyses: temperature

Temperature analysis will be performed in the neonatal strata only.

A summary table will be presented for all babies randomised to treatment groups:

- Temperature at baseline: mean (SD) [N]
- Temperature pre-application at all time points: mean (SD) [N]
- Temperature at post-application at all time points: mean (SD) [N]

Temperature analysis

- The analysis will include all babies randomised to treatment with at least one antiseptic application. Babies in the SOC group will be excluded from the analysis.
- The outcome variable will be:
  - Change in temperature from pre to post application.
- Fixed effects in the model will be:
  - Antiseptic (1% CHG, 2% CHG, OCT)
  - Frequency of application (once, multiple)
  - Day of trial (continuous; mfp will be used to assess linearity)
  - Temperature pre application (continuous; mfp will be used to assess linearity)
- Individual will be fitted as a random effect to account for repeated measures within individuals.
- Normally distributed errors will be fitted.
- Comparison of main effects within arms will be assessed using the estimate and 95% confidence interval of the comparison of differences between factors and in comparison, to SOC.
- The reference group will be 1% CHG once only as it is the most straightforward and conservative to implement. The SOC will not be used as the reference as the sample size for this arm is lower than for the factorial arms.

Additional analyses, as per the primary outcomes, may also be performed.

DocuSign Envelope ID: F49E6E37-298A-446A-A83D-BE73F1829E60

4.10. Secondary safety outcome analyses: AEs and SAEs

SAEs and AEs will be presented overall and split by MedDRA System Order Class (SOC) and Preferred Term (PT). AEs and SAEs will be displayed as n(%)M where M is the number of events experienced for all children experiencing at least one event (M>n).

- SAEs: n(%)M
- Antiseptic related SAEs: n(%)M
- Grade 3 or 4 AEs: n(%)M
- Grade 3 or 4 skins scores (appendix I and II of protocol) : n(%)M
- Grade 3 or 4 hypothermia in neonates only (section 4.7.3 of protocol) : n(%)M

AE and SAE analysis

- Frequency of SAEs will be compared using exact logistic models.
- Time-to-event models will be used if AEs/SAEs occur in >10% of the trial population overall.

4.11. Additional secondary efficacy outcomes analysis

Log CFU of specific species

- Will be analysed as per the primary outcome.
- Analyses beyond the primary analysis will be performed if considered necessary.

Acquisition and loss of specific species

- Will be analysed as per the primary outcome except Binomial generalised linear mixed models will be fitted with logit link will be used.
- Analyses beyond the primary analysis will be performed if considered necessary.
- Margins will be taken for presentation of results.

Components of total CFU considered separately

- Will be analysed as per the primary outcome.
- Analyses beyond the primary analysis will be performed if considered necessary.

Components of skin scores considered separately

- Will be analysed as per total skin score above.
- Analyses beyond the primary analysis will be performed if considered necessary.

5. References

1. Hernán MA, Robins JM. Per-protocol analyses of pragmatic trials. N Engl J Med. 2017;377(14):1391–8.

2. Sankar MJ, Paul VK, Kapil A, Kalaivani M, Agarwal R, Darmstadt GL, et al. Does skin cleansing with chlorhexidine affect skin condition, temperature and colonization in hospitalized preterm low birth weight infants?: a randomized clinical trial. J Perinatol. 2009;29(12):795–801.

3. Johnson J, Suwantarat N, Colantuoni E, Ross TL, Aucott SW, Carroll KC, et al. The impact of chlorhexidine gluconate bathing on skin bacterial burden of neonates admitted to the Neonatal Intensive Care Unit. J Perinatol. 2019;39(1):63–71.

4. Schulz KF, Altman DG, Moher D. CONSORT 2010 statement: updated guidelines for reporting parallel group randomised trials. J Pharmacol Pharmacother. 2010;1(2):100–7.

5. Clements MN, White IR, Copas AJ, Cornelius V, Cro S, Dunn DT, et al. Improving clinical trial interpretation with ACCEPT analyses. NEJM Evid. 2022;1(8):EVIDctw2200018.

6. Appendix: Bayesian priors

6.1. Non-informative priors

Non informative priors were selected to capture a wide range of possible values. The same priors will be used for the maternal and the neonatal strata.

- Intercept mean =4, sd = 50
- Difference mean =0, sd = 20

6.2. Informative priors (optimistic and sceptical)

Informative priors were determined based on NeoCHG data (unpublished) and expert clinician opinion (Nicholas Feasey, David Lissauer, Emily Beales, Louise Hill, Neal Russell) during an online meeting on 21/03/2023. There is a lack of robust data to support the use of strong priors, especially in the maternal strata.

Intercept specification

The intercept (Control) was selected as data from NeoCHG could be used. The maternal strata will use data from d8 in NeoCHG, reflecting bacterial load with time to accumulate, whereas the neonatal strata will use data from baseline reflecting the recent birth of the neonates. Using mean values from the study and approximations of sd with a multiplier on the sd to capture extra uncertainty, the intercepts will be specified as following a normal distribution with parameters:

- Log CFU in maternal strata: mean 8.4; standard deviation 2.6; multiplier 3
- Log CFU in neonatal strata: mean 4.5; standard deviation 3; multiplier 2

A higher multiplier was chosen for the maternal strata as there is greater uncertainty in the expected values. Priors may be reformulated to a reference of 1%CHG/once before analysis to match reference specification in focal analysis.

Difference specification

Clinicians specified the mean difference that optimistic and sceptical persons knowledgeable on the topic might assume, using a reference of no antiseptic application. The approach taken was that both optimistic and sceptical persons were assumed to have some belief that the opposing view could be correct, given the assumption of equipoise for the trial to be able to go ahead. The mean values selected were:

Maternal strata

Parameter	Optimistic mean	Sceptical mean
0.5% CHG	Lower by 0.5	No effect
2% CHG	Lower by 1	No effect
OCT	Lower by 1	No effect
Single application	Lower by 0.5	No effect

Multiple application	Lower by 1	No effect

Neonatal strata

**Parameter**	**Optimistic mean**	**Sceptical mean**
0.5% CHG	Lower by 0.5	No effect
2% CHG	Lower by 1	No effect
Emollient	Lower by 1	No effect
Alternate day application	Lower by 0.5	No effect
Control	Lower by 1	No effect

Larger differences were selected than those found in NeoCHG due to the differing site conditions in NeoVT-AMR.

Differences were specified to follow a Normal distribution with mean as above and a certain percentage of the distribution more extreme than the opposing value (sceptical for optimistic priors and optimistic for sceptical priors) reflecting probability of alternate scenario being correct. This equates to a Normal distribution with:

- Mean = optimistic or sceptical mean
- sd = abs(sceptical mean – optimistic mean)/critical_value

Where the critical value is from the t-distribution with infinite degrees of freedom and probability equal to the alternate scenario being correct.

The probability of the alternate scenario being correct was

- Maternal strata: 0.2
- Neonatal strata: 0.1

A larger probability was chosen for the maternal strata to reflect the greater uncertainty in the this strata.

Plots of the differences are:

Maternal strata

DocuSign Envelope ID: F49E6E37-298A-446A-A83D-BE73F1829E60

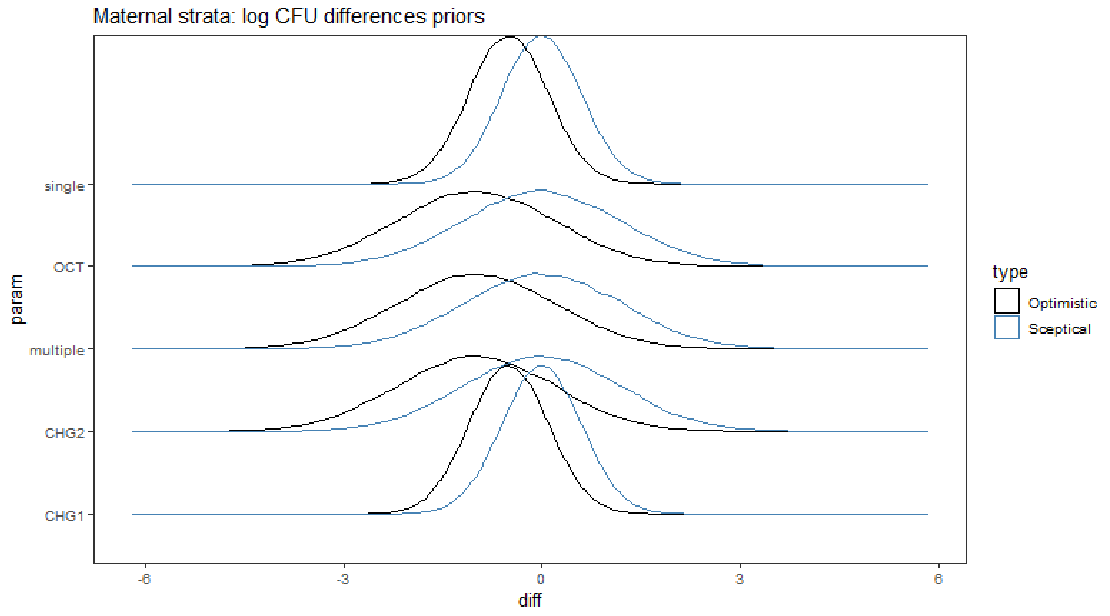

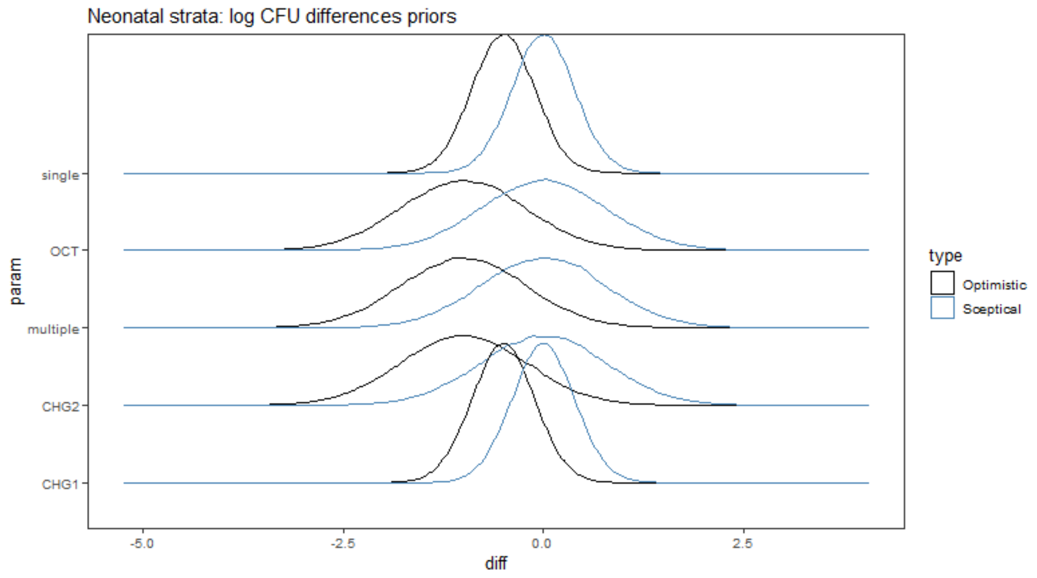

Note the difference scales in each graph.
