# Supplementary material for: Antiseptic Cleansing to Reduce Vertical Transmission of Pathogens to Neonates: The NeoVT-AMR Randomized Clinical Trial
Source: JAMA Netw Open. 2026 Jun 11;9(6):e2615665. doi: 10.1001/jamanetworkopen.2026.15665 (PMC13261496; doi:10.1001/jamanetworkopen.2026.15665)
Supplement: Supplement 2. — eAppendix. Protocol Amendments eMethods 1. Skin Score Assessment Criteria eMethods 2. Six-Swab Technique for Maternal Antiseptic Application eMethods 3. Microbiological Sampling and Laboratory Procedures eMethods 4. Adverse Event Grading Scales (Maternal) eMethods 5. Adverse Event Grading Scales (Neonatal) eMethods 6. Priors for Bayesian ACCEPT Analyses eFigure 1. Maternal Bacterial-Load Trajectories eFigure 2. Neonatal Bacterial-Load Trajectories eTable 1. Additional Baseline Variables (Maternal) eTable 2. Additional Baseline Variables (Neonatal) eTable 3. Intervention Delivery (Maternal) eTable 4. Intervention Delivery (Neonatal) eTable 5. Primary and Secondary Outcomes (Maternal) eTable 6. Primary and Secondary Outcomes (Neonatal) eTable 7. Main Effects Interaction Analyses (Maternal) eTable 8. Main Effects Interaction Analyses (Neonatal) eTable 9. Subgroup Analyses (Maternal) eTable 10. Subgroup Analyses (Neonatal) eTable 11. ACCEPT Posterior Probability Analyses (Maternal) eTable 12. ACCEPT Posterior Probability Analyses (Neonatal) eTable 13. Sensitivity Analyses (Maternal) eTable 14. Sensitivity Analyses (Neonatal) eTable 15. SAE Summary Tables (Maternal) eTable 16. SAE Summary Tables (Neonatal) eTable 17. SAE Line Listing (Maternal) eTable 18. SAE Line Listing (Neonatal) eReferences [file jamanetwopen-e2615665-s002.pdf]

Supplementary Online Content

Beales E, Clements MN, Feasey NA, et al. Antiseptic cleansing to reduce vertical transmission of pathogens to neonates: the NeoVT-AMR randomized clinical trial. *JAMA Netw Open*. 2026;9(6):e2615665. doi:10.1001/jamanetworkopen.2026.15665

eAppendix. Protocol Amendments
eMethods 1. Skin Score Assessment Criteria
eMethods 2. Six-Swab Technique for Maternal Antiseptic Application
eMethods 3. Microbiological Sampling and Laboratory Procedures
eMethods 4. Adverse Event Grading Scales (Maternal)
eMethods 5. Adverse Event Grading Scales (Neonatal)
eMethods 6. Priors for Bayesian ACCEPT Analyses
eFigure 1. Maternal Bacterial-Load Trajectories
eFigure 2. Neonatal Bacterial-Load Trajectories
eTable 1. Additional Baseline Variables (Maternal)
eTable 2. Additional Baseline Variables (Neonatal)
eTable 3. Intervention Delivery (Maternal)
eTable 4. Intervention Delivery (Neonatal)
eTable 5. Primary and Secondary Outcomes (Maternal)
eTable 6. Primary and Secondary Outcomes (Neonatal)
eTable 7. Main Effects Interaction Analyses (Maternal)
eTable 8. Main Effects Interaction Analyses (Neonatal)
eTable 9. Subgroup Analyses (Maternal)
eTable 10. Subgroup Analyses (Neonatal)
eTable 11. ACCEPT Posterior Probability Analyses (Maternal)
eTable 12. ACCEPT Posterior Probability Analyses (Neonatal)
eTable 13. Sensitivity Analyses (Maternal)
eTable 14. Sensitivity Analyses (Neonatal)
eTable 15. SAE Summary Tables (Maternal)
eTable 16. SAE Summary Tables (Neonatal)
eTable 17. SAE Line Listing (Maternal)
eTable 18. SAE Line Listing (Neonatal)
eReferences

This supplementary material has been provided by the authors to give readers additional information about their work.

© 2026 Beales E et al. *JAMA Network Open*.

eAppendix1. Protocol amendments

Final protocol version was version 6.0. Recruitment commenced on version 3.0.

Protocol versions	Summary of amendments	Comments
3.0 to 5.0	i) Inclusion criteria updated to allow recruitment of neonates born at any healthcare facility within the last 24 hours. Previous inclusion criterion only allowed recruitment of neonates born at Zomba Central Hospital within the last 12 hours.<br>ii) 28-day follow-up window increased from ±2 days to ±7 days.	There was no Protocol version 4.0. The College of Medicine Research & Ethics Committee (COMREC) requires version numbers of Protocols and corresponding Patient Information Sheets to be the same. Amendments were made to the Patient Information Sheet with no corresponding amendment to the Protocol and therefore, the protocol was updated to version 5.0 to correspond with the updates to the Patient Information Sheet.
5.0 to 6.0	The recruitment period was extended from January 2023 to March 2023.	

© 2026 Beales E et al. *JAMA Network Open*.

eMethods 1. Skin score assessment criteria

Neonatal population skin score assessment criteria

These criteria were adapted from Lund C & Osborne JW.[1] The skin should be scored on each of the three domains (dryness, erythema and skin breakdown) as per Table 1: Neonatal Skin Condition Score in Lund C & Osborne JW,[1] and then the total score is the sum of the three components (minimum score 0, maximum score 12). The skin affected area assessment should be made using the Lund and Browder chart for estimation of body surface area burn involvement in infants and children.[2]

Highest grade from:

Grade 1 = Score of ≥1 in ≥2 categories

Grade 2 = Score of ≥2 in ≥2 categories

Grade 3 = Score of ≥3 in ≥2 categories

Grade 4 = Score of ≥4 in ≥2 categories

© 2026 Beales E et al. *JAMA Network Open*.

Maternal population skin score assessment criteria

Adapted from NCI. National Cancer Institute Common Terminology Criteria for Adverse Events.[3]

The vaginal and vulval area should be scored on each of the four domains: 1 symptom (irritation) and 3 signs (erythema, skin breakdown, swelling). The total score is the sum of the three components (minimum score 0, maximum score 16).

Highest grade from:

Grade 1 = Score of ≥1 in ≥2 categories

Grade 2 = Score of ≥2 in ≥2 categories

Grade 3 = Score of ≥3 in ≥2 categories

Grade 4 = Score of ≥4 in ≥2 categories

PART A – Question to labouring women:

1. Vaginal/vulval irritation				
What is the severity of your vaginal irritation now at its worst?				
None = 0	Mild = 1	Moderate = 2	Severe = 3	Very severe = 4

PART B – On inspection of external genitalia:

2. Vaginal/vulval erythema				
What is the severity of erythema? (select the most severe that is present)				
None = 0	Mild = 1	Moderate = 2	Severe = 3	Very severe = 4

3. Vaginal/vulval skin breakdown				
What is the severity of skin breakdown? (select the most severe that is present)				
None = 0	Mild = 1	Moderate = 2	Severe = 3	Very severe = 4

4. Vaginal/vulval swelling				
What is the severity of swelling? (select the most severe that is present)				
None = 0	Mild = 1	Moderate = 2	Severe = 3	Very severe = 4

© 2026 Beales E et al. *JAMA Network Open*.

eMethods 2. Six-swab technique for maternal antiseptic application

Using six cotton wool balls soaked in antiseptic (chlorhexidine (CHG)1%, CHG2% or (octenidine 0.1% combined with phenoxyethanol 2% (OHP)) or sterile water (SOC).

1. Swab first labia majora with a downward motion using 1 swab per stroke (extending down to perineum)
2. Swab second labia majora with a downward motion using 1 swab per stroke (extending down to perineum)
3. Swab first labia minora with a downward motion using 1 swab per stroke
4. Swab second labia minora with a downward motion using 1 swab per stroke
5. Swab rest of vulva and vaginal introitus using 1 swab
6. Insert cotton wool into vagina and cervical os, using circular motions cleanse from cervical os outwards to cleanse the inside of the vagina

© 2026 Beales E et al. *JAMA Network Open*.

eMethods 3. Microbiological sampling and laboratory procedures

Swabs and processing

Swab collection was only performed by the study nurses to ensure standardization of sample collection. Dry flocked cotton swabs were used. In the maternal population, low vaginal and perineal swabs were taken at 0, 4, 8, 24, 28, 32 hours from randomization; if heavily soiled, the area was cleansed with sterile water prior to swabbing (see table below). In the neonatal population, neck (cervical skin fold) and peri-rectal swabs were taken; no skin preparation was used (see 'Colonization sampling procedure' below). Swabs were placed in liquid amies transport medium (LTM). Samples were refrigerated for 24-48 hours prior to being processed in the laboratory. Swabs in LTM were vortexed, and ten-fold dilutions of the LTM with saline were performed, down to a dilution of 10^{-4}. 20µL aliquots of the neat LTM were inoculated onto UriSelect agar (Bio Rad, Hercules, USA) and blood sheep agar (made in-house); and the 10^{-2} and 10^{-4} dilutions were inoculated onto UriSelect agar only. Agar plates were incubated overnight (18-20 hours) at 35°C in air.

The UriSelect and blood sheep agar plates with colonies most suitable for colony counts were selected for manual colony counting. Total number of colonies as well as colony counts for Gram-positive organisms, Gram-negative organisms and any identified species were also recorded. The colony counts were converted to $\log_{10}$CFU.

Colonization sampling procedure

Sampling site	Sampling method
**Maternal population**	
Low vaginal	Skin outside the vagina was gently spread with one hand. The swab tip was inserted into the vaginal introitus, and gently slid 3 cm into the vagina. The swab was gently rotated 360 degrees 10 times, taking 1 second for each turn. The swab tip was withdrawn without touching the skin.
Perineal	The perineum was wiped with cotton wool soaked in tap water when visibly soiled.<br>Taking care to avoid other contact with the swab, the swab was rotated 360 degrees against the perineal skin (the area between the anus and external genitalia) for 5 seconds from one side to the next. This was then repeated one further time.
**Neonatal population**	
Neck (cervical skin fold)	The swab was applied between the neck folds whilst continuously rotating the tip backwards and forwards. The anterior 180-degree portion of the neck was swabbed. The process was repeated one further time.
Peri-rectal area	The swab was rotated 360 degrees around the peri-rectal area whilst continuously rotating the tip. This was then repeated one further time.

Organism identification

Organisms were presumptively identified based on the colony color on the UriSelect agar (Bio-Rad, Redmond, USA) with more precise identification undertaken, where relevant, using Gram staining as well as specific identification tests. Color and indole tests were used to differentiate *Escherichia coli* from other Enterobacterales. Potential *Klebsiella* spp., *Serratia spp.* and *Enterobacter* spp. (purple-blue colonies), were

© 2026 Beales E et al. *JAMA Network Open*.

identified using the Analytical Profile Index (API) (20E) (Biomerieux, Marcy-l'Etoile, France). Oxidase was adopted to differentiate Gram-negative bacilli (Pseudomonas spp. or *Acinetobacter* spp.). Catalase was used to differentiate Gram-positive cocci (*Staphylococcus* spp. or *Streptococcus* spp.). Coagulase was used on catalase positive organisms to differentiate *Staphylococcus aureus* from other *Staphylococcus* spp.. Catalase negative organisms were further differentiated based on the type of hemolysis on blood agar (α- and β-haemolyticus streptococci) and Lancefield typing (β-haemolyticus streptococci groups A or B) was conducted using the Streptococcal Latex Grouping Kit (Pro-Lab, Ontario, Canada).

© 2026 Beales E et al. *JAMA Network Open*.

eMethods 4. Adverse event grading scales (maternal)

Adapted from Common Terminology Criteria for Adverse Events[3]

Maternal population

Parameter	Grade 0 Normal	Grade 1 Mild	Grade 2 Moderate	Grade 3 Severe	Grade 4 Potentially Life-Threatening	Grade 5
Blood and lymphatic system disorders						
Anaemia	Haemoglobin (Hb) >10g/dL	Hb <10g/dL	Hb 8-10 g/dL	Hb ≤ 8 g/dL; transfusion indicated	Life threatening consequences; urgent intervention indicated	Death
Disseminated intravascular coagulation	–	–	Laboratory findings with no bleeding	Laboratory findings and bleeding	Life threatening consequences; urgent intervention indicated	Death
Hemolysis	–	Laboratory evidence of haemolysis only (e.g. direct antiglobulin test (DAT); Coombs; schistocytes; decreased haptoglobin)	Evidence of haemolysis and ≥2g decrease in haemoglobin	Transfusion or medical intervention indicated (e.g. steroids)	Life threatening consequences; urgent intervention indicated	Death
Blood and lymphatic system disorders – other	–	Asymptomatic or mild symptoms; clinical or diagnostic observations only; intervention not indicated	Moderate; minimal, local or non-invasive intervention indicated; limiting age-appropriate instrumental activities of daily living (ADL)	Severe or medically significant but not immediately life-threatening; hospitalization or prolongation of existing hospitalization indicated; limiting self-care ADL	Life-threatening consequences; urgent intervention indicated	Death
Cardiac disorders						

© 2026 Beales E et al. *JAMA Network Open*.

Parameter	Grade 0 Normal	Grade 1 Mild	Grade 2 Moderate	Grade 3 Severe	Grade 4 Potentially Life-Threatening	Grade 5
Sinus tachycardia	–	Asymptomatic, intervention not indicated	Symptomatic, non-urgent medical intervention indicated	Urgent medical intervention indicated	–	- –
Supraventricular tachycardia	–	Asymptomatic, intervention not indicated	Non-urgent medical intervention indicated	Symptomatic, urgent intervention indicated	Life threatening consequences	Death
Cardiac disorders – other, specify	–	Asymptomatic or mild symptoms; clinical or diagnostic observations only; intervention not indicated	Moderate; minimal, local or non-invasive intervention indicated; limiting age-appropriate instrumental ADL	Severe or medically significant but not immediately life-threatening; hospitalisation or prolongation of existing hospitalisation indicated; limiting self-care ADL	Life-threatening consequences; urgent intervention indicated	Death
Gastrointestinal						
Anal fistula	Asymptomatic	Symptomatic	Invasive intervention not indicated	Invasive intervention indicated	Life threatening consequences; urgent intervention indicated	–
Haemorrhoids	–	Asymptomatic; clinical or diagnostic observations only; intervention not indicated	Symptomatic, banding or medical intervention indicated	Severe symptoms, invasive intervention indicated	–	–
Haemorrhoidal haemorrhage	–	Mild symptoms; intervention not indicated	Moderate symptoms; intervention indicated	Transfusion indicated; invasive intervention indicated; hospitalisation	Life-threatening consequences; urgent intervention indicated	Death
Nausea	–	Loss of appetite without	Oral intake decreased without	Inadequate oral caloric or fluid intake; tube	–	–

© 2026 Beales E et al. *JAMA Network Open*.

Parameter	Grade 0 Normal	Grade 1 Mild	Grade 2 Moderate	Grade 3 Severe	Grade 4 Potentially Life-Threatening	Grade 5
		alteration in eating habits	significant weight loss, dehydration or malnutrition	feeding, TPN, or hospitalization indicated		
Vomiting	–	Intervention not indicated	Outpatient intravenous (IV) hydration; medical intervention indicated	Tube feeding, total parenteral nutrition (TPN) or hospitalisation indicated	Life-threatening consequences	Death
Ileus	–	Asymptomatic and radiologic observations only	Symptomatic, altered GI function, bowel rest indicated	Severely altered GI function; TPN indicated, tube placement indicated	Life-threatening consequences; urgent intervention indicated	Death
General disorders						
Neonatal death	–	–	–	–	Neonatal loss of life	–
Oedema of limbs	–	5-10% inter-limb discrepancy in volume or circumference at point of greatest visible difference; swelling or obscuration of anatomic architecture on close inspection	>10-30% inter-limb discrepancy in volume or circumference at point of greatest visible difference; readily apparent obscuration of anatomic architecture; obliteration of skin folds; readily apparent deviation from normal anatomic contour; limiting instrumental ADL	>30% inter-limb discrepancy in volume; gross deviation from normal anatomic contour; limiting self-care ADL	–	–
Fever	–	38.0–39.0°C	39.0–40.0°C	>40.0°C for <24 hours	>40.0°C for >24 hours	Death
Immune system disorders						
Allergic reaction	–	Systemic intervention	Oral intervention indicated	Bronchospasm; hospitalisation	Life-threatening consequences;	Death

© 2026 Beales E et al. *JAMA Network Open*.

Parameter	Grade 0 Normal	Grade 1 Mild	Grade 2 Moderate	Grade 3 Severe	Grade 4 Potentially Life-Threatening	Grade 5
		not indicated		indicated for clinical sequelae; intravenous intervention indicated	urgent intervention indicated	
Anaphylaxis	–	–	–	Symptomatic bronchospasm, with or without urticaria; parenteral intervention indicated; allergy-related oedema/angioedema; hypotension	Life-threatening consequences; urgent intervention indicated	Death
Infections						
Abdominal infection	–	–	Oral intervention indicated	IV antibiotic, antifungal or antiviral intervention indicated; invasive intervention indicated	Life-threatening consequences; urgent intervention indicated	Death
Bacteraemia	–	–	Blood culture positive with no signs or symptoms			
Cervicitis infection	–	–	Localised, local intervention indicated (e.g. topical antibiotic, antifungal or antiviral)	IV antibiotic, antifungal or antiviral intervention indicated; invasive intervention indicated	Life-threatening consequences; urgent intervention indicated	Death
Pelvic infection	–	–	Moderate symptoms, oral	IV antibiotic, antifungal or antiviral	Life-threatening consequences; urgent	Death

© 2026 Beales E et al. *JAMA Network Open*.

Parameter	Grade 0 Normal	Grade 1 Mild	Grade 2 Moderate	Grade 3 Severe	Grade 4 Potentially Life-Threatening	Grade 5
			intervention indicated	intervention indicated; invasive intervention indicated	intervention indicated	
Sepsis	–	–	–	Blood culture positive with signs or symptoms; treatment indicated	Life threatening consequences; urgent intervention indicated	Death
Thrush	–	Asymptomatic; local symptomatic management	Oral intervention indicated	IV antifungal intervention indicated	–	–
Urinary tract infection	–	–	Localised oral intervention indicated	IV antibiotic, antifungal or antiviral intervention indicated; invasive intervention indicated	Life threatening consequences; urgent intervention indicated	Death
Uterine infection	–	–	Moderate symptoms; oral intervention indicated	IV antibiotic, antifungal or antiviral intervention indicated; invasive intervention indicated	Life threatening consequences; urgent intervention indicated	Death
Vaginal infection	–	Localised, local intervention indicated	Oral intervention indicated	IV antibiotic, antifungal or antiviral intervention indicated; invasive	Life threatening consequences; urgent intervention indicated	Death

© 2026 Beales E et al. *JAMA Network Open*.

Parameter	Grade 0 Normal	Grade 1 Mild	Grade 2 Moderate	Grade 3 Severe	Grade 4 Potentially Life-Threatening	Grade 5
				intervention indicated		
Vulval infection	–	Localised, local intervention indicated	Oral intervention indicated	IV antibiotic, antifungal or antiviral intervention indicated; invasive intervention indicated	Life threatening consequences; urgent intervention indicated	Death
Wound infection	–	Localised, local intervention indicated	Oral intervention indicated	IV antibiotic, antifungal or antiviral intervention indicated; invasive intervention indicated	Life threatening consequences; urgent intervention indicated	Death
Injury and procedural complications						
Intraoperative arterial injury; intraoperative gastrointestina l injury; intraoperative reproductive tract injury; urinary injury	–	Primary repair of injured organ/struc ture indicated	Partial resection of injured organ/structure indicated	Complete resection or reconstruction of injured organ/structure indicated; limiting self-care ADL	Life-threatening consequences; urgent intervention indicated	Death
Intraoperative haemorrhage	–	–	–	Postoperative invasive intervention indicated, hospitalisation	Life-threatening consequences; urgent intervention indicated	Death
Postoperative haemorrhage	–	Mild symptoms; intervention not indicated	Moderate bleeding requiring transfusion <2 units	Transfusion indicated ≥2 units; invasive intervention indicated; hospitalisation	Life-threatening consequences, urgent intervention indicated	Death

© 2026 Beales E et al. *JAMA Network Open*.

Parameter	Grade 0 Normal	Grade 1 Mild	Grade 2 Moderate	Grade 3 Severe	Grade 4 Potentially Life-Threatening	Grade 5
Uterine perforation	–		Invasive intervention not indicated	Invasive intervention indicated	Life-threatening consequences, urgent intervention indicated	Death
Wound complication	–	Observation only, topical intervention indicated	Bedside local care indicated	Operative intervention indicated	Life-threatening consequences	Death
Wound dehiscence	–	Incisional separation, intervention not indicated	Incisional separation, local care (e.g. suturing) or medical intervention indicated (e.g. analgesic)	Fascial disruption or dehiscence without evisceration; revision by operative intervention indicated	Life-threatening consequences; symptomatic hernia with evidence of strangulation; fascial disruption with evisceration; major reconstruction flap, grafting, resection	Death
Pregnancy, puerperium and perinatal conditions						
Fetal growth retardation	–	–	<10% percentile of weight for gestational age	<5% percentile of weight for gestational age	<1% percentile of weight for gestational age	–
Pregnancy loss	–	–	–	–	Fetal loss at any gestational age	–
Premature delivery	–	Delivery of a liveborn infant at >34 to 37 weeks gestation	Delivery of a liveborn infant at >28 to 34 weeks gestation	Delivery of a liveborn infant at 24 to 28 weeks gestation	Delivery of a liveborn infant at 24 weeks of gestation or less	–
Renal and urinary disorders						
Acute kidney injury	–	–	–	Hospitalisation indicated	Life-threatening consequences; dialysis indicated	Death
Reproductive system						

© 2026 Beales E et al. *JAMA Network Open*.

Parameter	Grade 0 Normal	Grade 1 Mild	Grade 2 Moderate	Grade 3 Severe	Grade 4 Potentially Life-Threatening	Grade 5
Vaginal discharge	Normal for patient	Mild vaginal discharge (greater than baseline for patient)	Moderate to heavy vaginal discharge; use of perineal pad or tampon indicated	–	–	–
Uterine haemorrhage	No haemorrhage	Mild symptoms; intervention not indicated	Moderate symptoms; intervention indicated	Transfusion indicated; invasive intervention indicated; hospitalisation	Life threatening consequences; urgent intervention indicated	Death
Vaginal haemorrhage	–	Mild symptoms; intervention not indicated	Moderate symptoms; intervention indicated	Transfusion indicated; invasive intervention indicated; hospitalisation	Life threatening consequences; urgent intervention indicated	Death
Vaginal perforation	–	–	Invasive intervention not indicated	Invasive intervention indicated	Life threatening consequences; urgent intervention indicated	Death
Respiratory						
Hypoxia	–	–	Decreased oxygen saturation with exercise (e.g. pulse oximeter <88%); intermittent supplemental oxygen	Decreased oxygen saturation at rest (e.g. pulse oximeter <88%)	Life threatening compromise; urgent intervention needed	Death
Vascular						
Thromboembolic event	–	Medical intervention not indicated (e.g.	Medical intervention indicated	Urgent medical intervention indicated (e.g. pulmonary embolism)	Life-threatening consequences with haemodynamic or	Death

© 2026 Beales E et al. *JAMA Network Open*.

Parameter	Grade 0 Normal	Grade 1 Mild	Grade 2 Moderate	Grade 3 Severe	Grade 4 Potentially Life-Threatening	Grade 5
		superficial thrombosis)			neurologic instability	

© 2026 Beales E et al. *JAMA Network Open*.

eMethods 5. Adverse event grading scales (neonatal)

Neonatal population

Adapted DAIDS score for neonates reproduced from the IMPAACT study[4]

Parameter	Grade 0 Normal	Grade 1 Mild	Grade 2 Moderate	Grade 3 Severe	Grade 4 Potentially Life-Threatening
Apnoea*<br><br>*Apnoea spell defined as apnoea event >20s or associated with bradycardia, hypoxia or cyanosis	No apnoeas	<6 spells* per day	6<12 spells* per day or nasal cannula for apnoea	12 or more spells* per day or nasal continuous positive airway pressure (NCPAP) for apnoea	Requires intubation for apnoea
Anaemia	Haemoglobin (Hb) >10 g/dL	Hb 8 – 10 g/dL	Hb ≤8 g/dL	Requires packed red cell transfusion, no clinical signs	Requires packed red cell transfusion, clinical signs of shock
Congenital anomalies	None	Minor (no impairment of function)	Minor (no impairment of function), future treatment may be needed	Major (impairment of function), no immediate treatment needed	Major (impairment of function), immediate treatment needed
Congenital heart disease	No congenital heart disease	Minor (no impairment of function), no treatment needed	Minor (no impairment of function), future treatment may be needed	Major (impairment of function), no immediate treatment needed	Major (impairment of function), immediate treatment needed
Electrolyte/ Metabolic disorders	None	------------------	Electrolyte/Metabolic disorder, no systemic signs	------------------------	Electrolyte/Metabolic disorder with systemic signs

© 2026 Beales E et al. *JAMA Network Open*.

Parameter	Grade 0 Normal	Grade 1 Mild	Grade 2 Moderate	Grade 3 Severe	Grade 4 Potentially Life-Threatening
Gastrointestinal dysfunction [including necrotising enterocolitis (NEC)]	None	Not ill, abnormal abdominal exam, no treatment needed (e.g. abdominal distension but not NPO)	Mild illness, nil per os (NPO) <3 days (e.g. rule out NEC evaluation, Stage I NEC)	Moderate illness, NPO 3 – 7 days, or medical treatment (e.g. Stage II NEC)	Severe illness, NPO >7 days, or surgical treatment needed (e.g. Stage III NEC)
Hypertension	No blood pressure (BP) performed	Systolic BP >80–100, no treatment	Systemic BP >100, no treatment	Treated with one agent	Treatment with multiple agents
Hypotension	No BP performed	Mild clinical signs, no treatment needed	Symptomatic, treated with intravenous fluids	Symptomatic, treated with single medication	Clinical signs of shock or requiring use of multiple medications
Intraventricular haemorrhage	No abnormality noted or not assessed	Germinal matrix haemorrhage	Blood in ventricle, no enlargement	Blood in ventricle, with ventricular enlargement	Parenchymal haemorrhage and/or need for ventricular drainage
Jaundice	No jaundice	Mild jaundice, no treatment	Phototherapy and/or intravenous immunoglobulin	Exchange transfusion	Acute bilirubin encephalopathy
Neonatal abstinence syndrome (NAS)	No history of abstinence syndrome	NAS signs, no medical treatment	NAS controlled with single drug	NAS controlled with two drugs	NAS with seizures
Neurologic compromise	Normal neurological examinations	Mildly abnormal neurologic exam, no clinical or electroencephalogram (EEG) seizure activity	Stage I encephalopathy or possible clinical or EEG seizure activity but no treatment	Stage II encephalopathy or single drug seizure therapy	Stage III encephalopathy or multiple drug seizure therapy

© 2026 Beales E et al. *JAMA Network Open*.

Parameter	Grade 0 Normal	Grade 1 Mild	Grade 2 Moderate	Grade 3 Severe	Grade 4 Potentially Life-Threatening
Neutropenia	Absolute neutrophil count (ANC) >1000/mm^3	ANC <1000/mm^3	ANC <500/mm^3	Treated with granulocyte colony-stimulating factor	White blood cell transfusion
Patent ductus arteriosus	No murmur	Clinical signs, no treatment	Treatment with fluid restriction or diuretics	Treatment with indomethacin or ibuprofen	Surgical ligation
Persistent pulmonary hypertension	No difference in pre- and post-ductal difference	Supplemental O_2 but no mechanical ventilation	Conventional ventilator <5 days	Conventional ventilation 5 – 10 days, alternative ventilation (e.g. high frequency oscillatory ventilation (HFOV), sildenafil and/or nitric oxide	Mechanical ventilation >10 days
Renal dysfunction	Wet diapers documented	Urine output 1 < 1.5 mL/kg/hr	Urine output 0.5 < 1.0 mL/kg/hr	Urine output 0 < 0.5 mL/kg/hr	Prolonged anuria
Respiratory Insufficiency	Baby is in room air – no supplemental oxygen	Nasal cannula oxygen with FiO_2 <0.5	Continuous positive airway pressure (CPAP) or nasal cannula with FiO_2 >0.5	Conventional ventilation	Alternative ventilation (e.g. HFOV)
Retinopathy of prematurity (ROP)	Normal vascularisation or not assessed	Incomplete vascularisation	Pre-threshold ROP	Threshold ROP or ROP treatment	Retinal detachment
Sepsis	No sepsis symptoms and signs	Septic evaluation, no treatment	Rule out sepsis,	A confirmed blood stream infection with	A confirmed blood stream infection with

© 2026 Beales E et al. *JAMA Network Open*.

Parameter	Grade 0 Normal	Grade 1 Mild	Grade 2 Moderate	Grade 3 Severe	Grade 4 Potentially Life-Threatening
			antibiotics for ≤72 hours	a course of antibiotic treatment, no septic shock or meningitis	a course of antibiotic treatment with septic shock or meningitis
Thrombocytopaenia	>100,000 cells/mm^3	75,000-100,000 cells/mm^3	50,000 < 75,000 cells/mm^3	25,000<50,000 cells/mm^3	<25,000 cells/mm^3 or platelet transfusion

© 2026 Beales E et al. *JAMA Network Open*.

eMethods 6. Priors for Bayesian ACCEPT analyses

Non-informative priors

Non informative priors were selected to capture a wide range of possible values. The same priors will be used for the maternal and the neonatal population.

- Intercept mean =4, SD = 50
- Difference mean =0, SD = 20

Informative priors (optimistic and sceptical)

Informative priors were determined based on NeoCHG data (unpublished) and expert clinician opinion (Nicholas Feasey, David Lissauer, Emily Beales, Louise F Hill, Neal Russell) during an online meeting on 21/03/2023. There is a lack of robust data to support the use of strong priors, especially in the maternal population.

Intercept specification

The intercept (SOC) was selected as data from NeoCHG could be used. The maternal population will use data from d8 in NeoCHG, reflecting bacterial load with time to accumulate, whereas the neonatal population will use data from baseline reflecting the recent birth of the neonates. Using mean values from the study and approximations of SD with a multiplier on the SD to capture extra uncertainty, the intercepts will be specified as following a normal distribution with parameters:

- $\log_{10}$CFU in maternal population: mean 8.4; standard deviation 2.6; multiplier 3
- $\log_{10}$CFU in neonatal population: mean 4.5; standard deviation 3; multiplier 2

A higher multiplier was chosen for the maternal population as there is greater uncertainty in the expected values. Priors may be reformulated to a reference of 1%CHG/once before analysis to match reference specification in focal analysis.

Difference specification

Clinicians specified the mean difference that optimistic and sceptical persons knowledgeable on the topic might assume, using a reference of no antiseptic application. The approach taken was that both optimistic and sceptical persons were assumed to have some belief that the opposing view could be correct, given the assumption of equipoise for the trial to be able to go ahead. The mean values selected were:

Maternal population

Parameter	Optimistic mean	Sceptical mean
0.5% CHG	Lower by 0.5	No effect
2% CHG	Lower by 1	No effect
OHP	Lower by 1	No effect
Single application	Lower by 0.5	No effect
Multiple application	Lower by 1	No effect

CHG = chlorhexidine; OHP = octenidine 0.1% combined with phenoxyethanol 2%

Neonatal population

Parameter	Optimistic mean	Sceptical mean
0.5% CHG	Lower by 0.5	No effect
2% CHG	Lower by 1	No effect
Emollient	Lower by 1	No effect
Alternate day application	Lower by 0.5	No effect
SOC	Lower by 1	No effect

CHG = chlorhexidine; OHP = octenidine 0.1% combined with phenoxyethanol 2%

Larger differences were selected than those found in NeoCHG due to the differing site conditions in NeoVT-AMR.

Differences were specified to follow a Normal distribution with mean as above and a certain percentage of the distribution more extreme than the opposing value (sceptical for optimistic priors and optimistic for sceptical priors) reflecting probability of alternate scenario being correct. This equates to a Normal distribution with:

- Mean = optimistic or sceptical mean
- SD = abs(sceptical mean – optimistic mean)/critical_value

Where the critical value is from the t-distribution with infinite degrees of freedom and probability equal to the alternate scenario being correct.

The probability of the alternate scenario being correct was:

- Maternal population: 0.2
- Neonatal population: 0.1

A larger probability was chosen for the maternal population to reflect the greater uncertainty in this population.

Plots of the differences are:

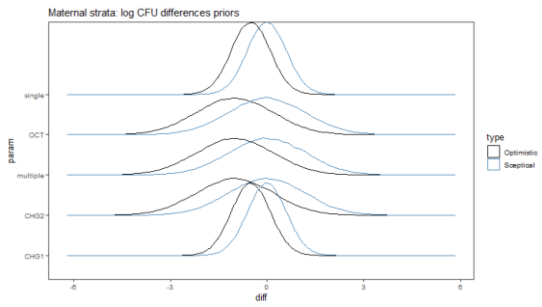

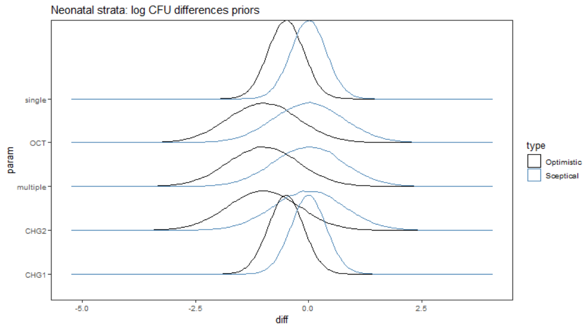

Note: different scales in each graph

© 2026 Beales E et al. *JAMA Network Open*.

eFigure 1. Maternal bacterial-load trajectories

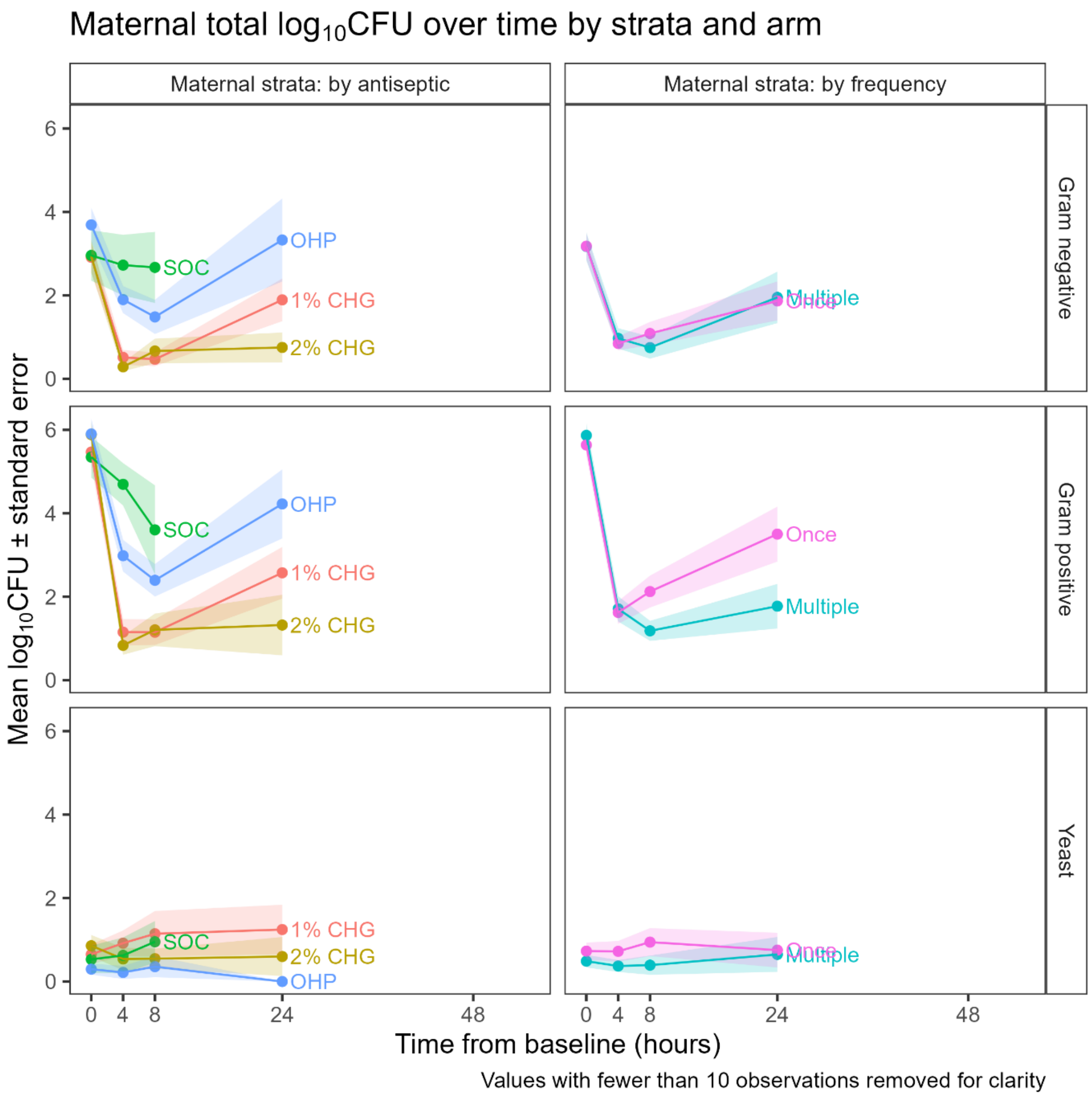

© 2026 Beales E et al. *JAMA Network Open*.

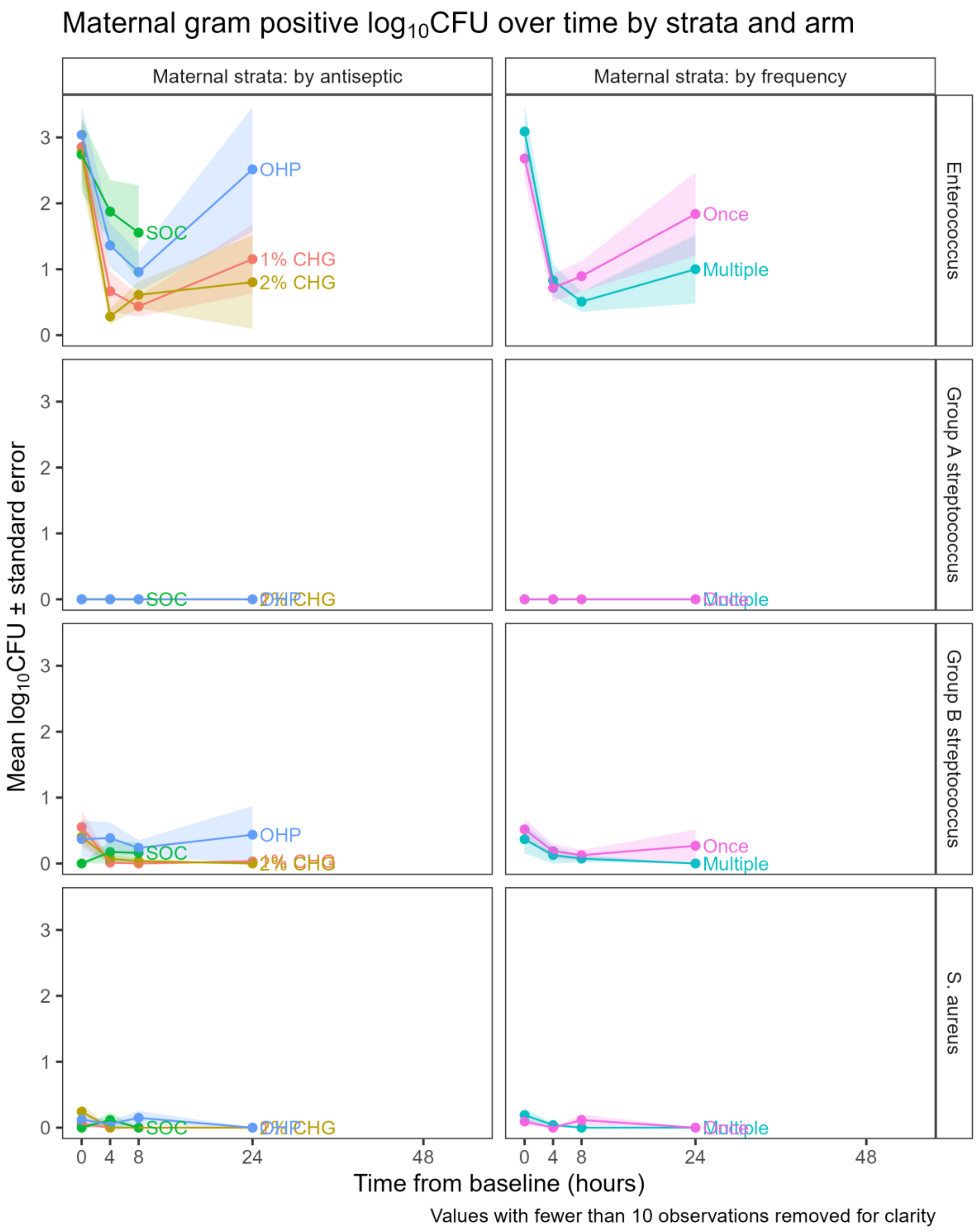

© 2026 Beales E et al. *JAMA Network Open*.

Maternal gram negative $\log_{10}$CFU over time by strata and arm

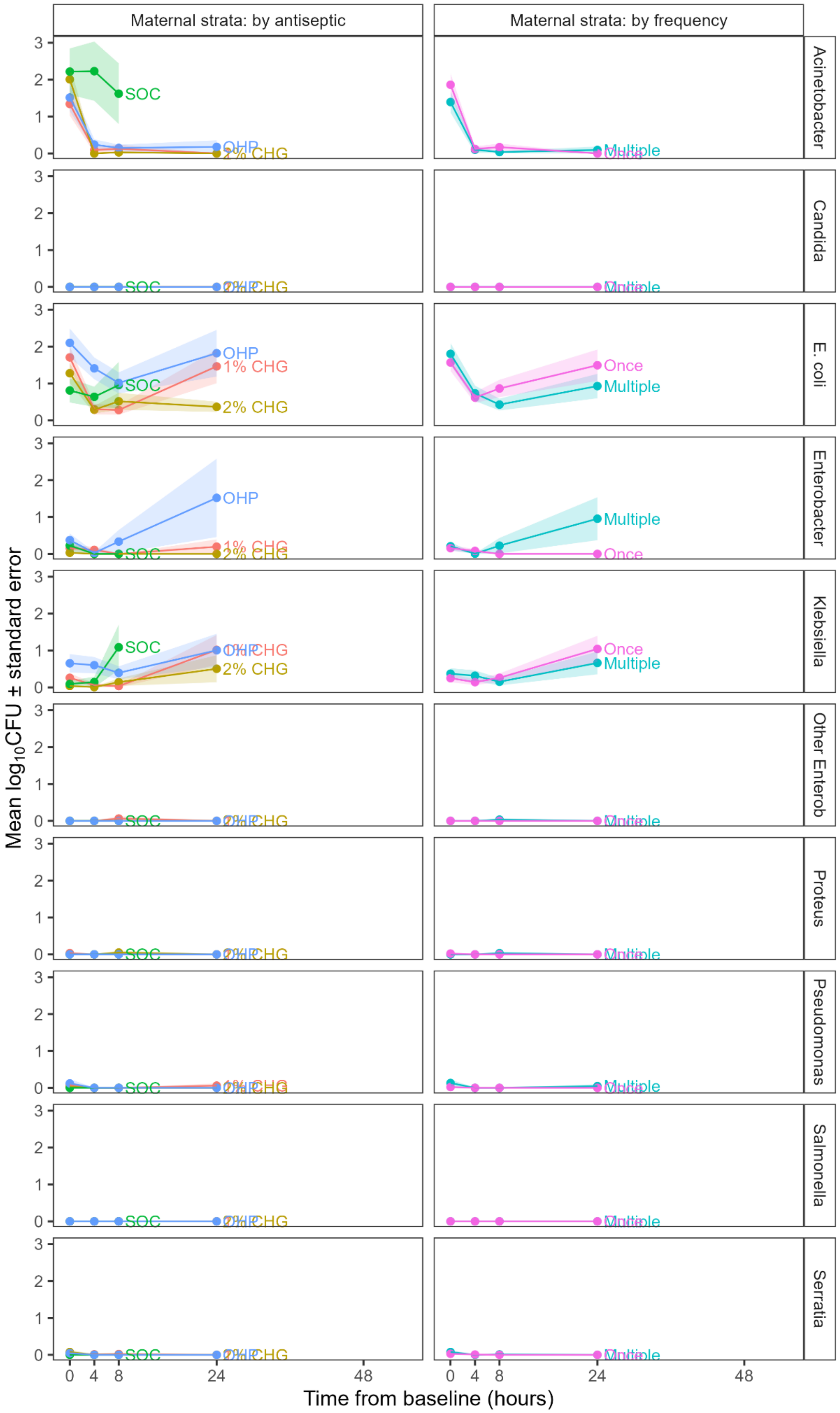

Values with fewer than 10 observations removed for clarity

eFigure 2. Neonatal bacterial-load trajectories

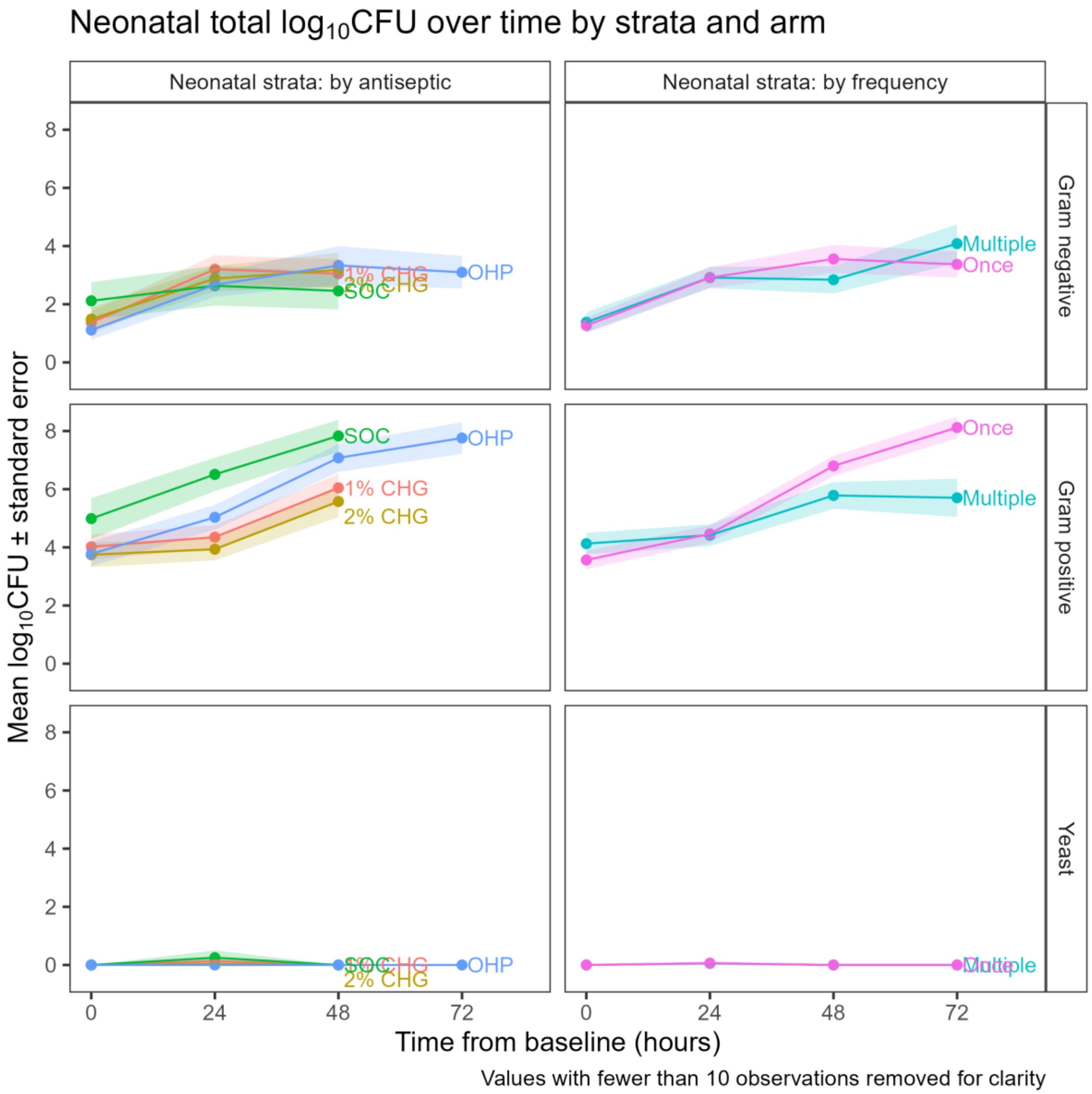

© 2026 Beales E et al. *JAMA Network Open*.

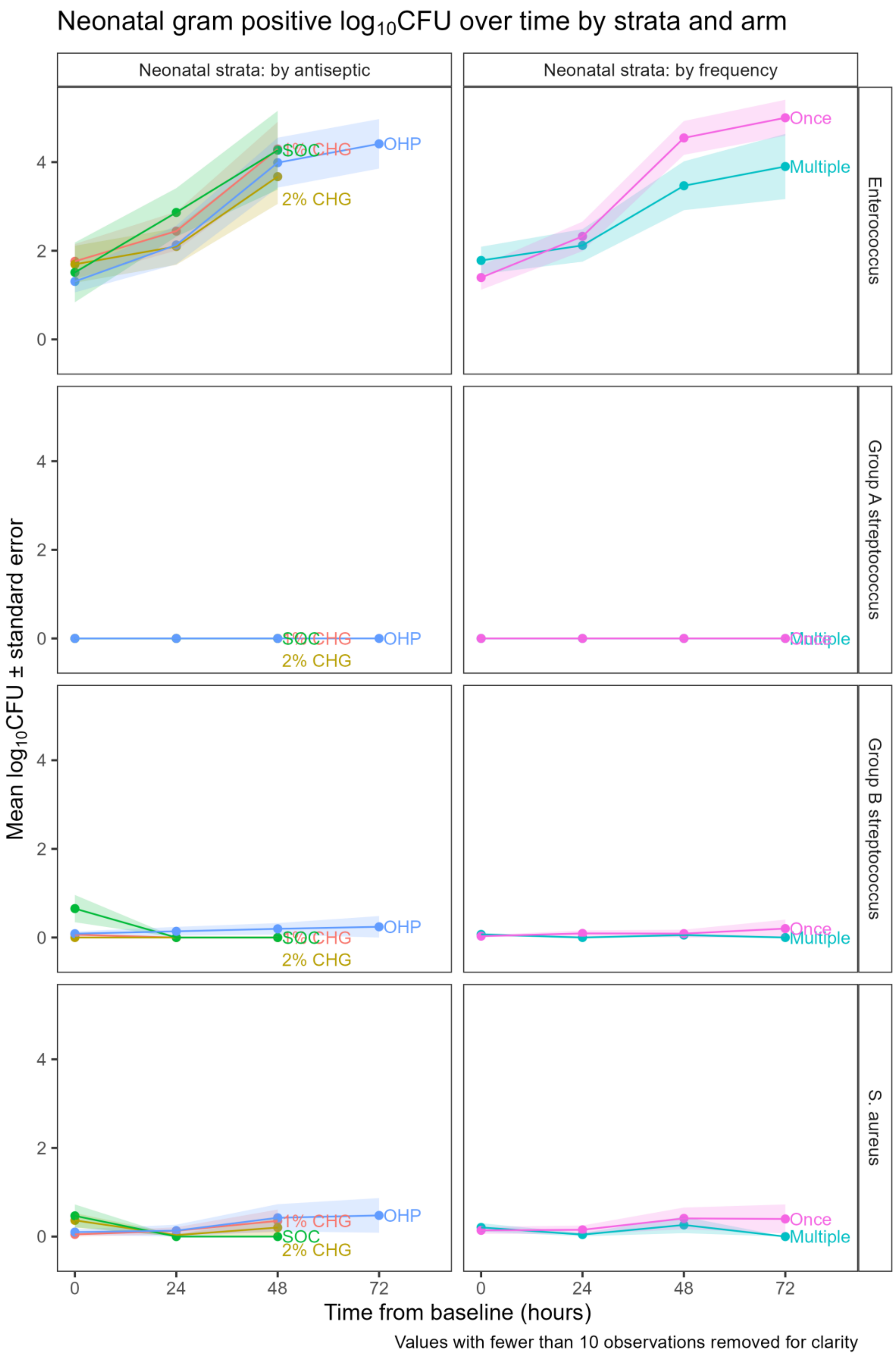

© 2026 Beales E et al. *JAMA Network Open*.

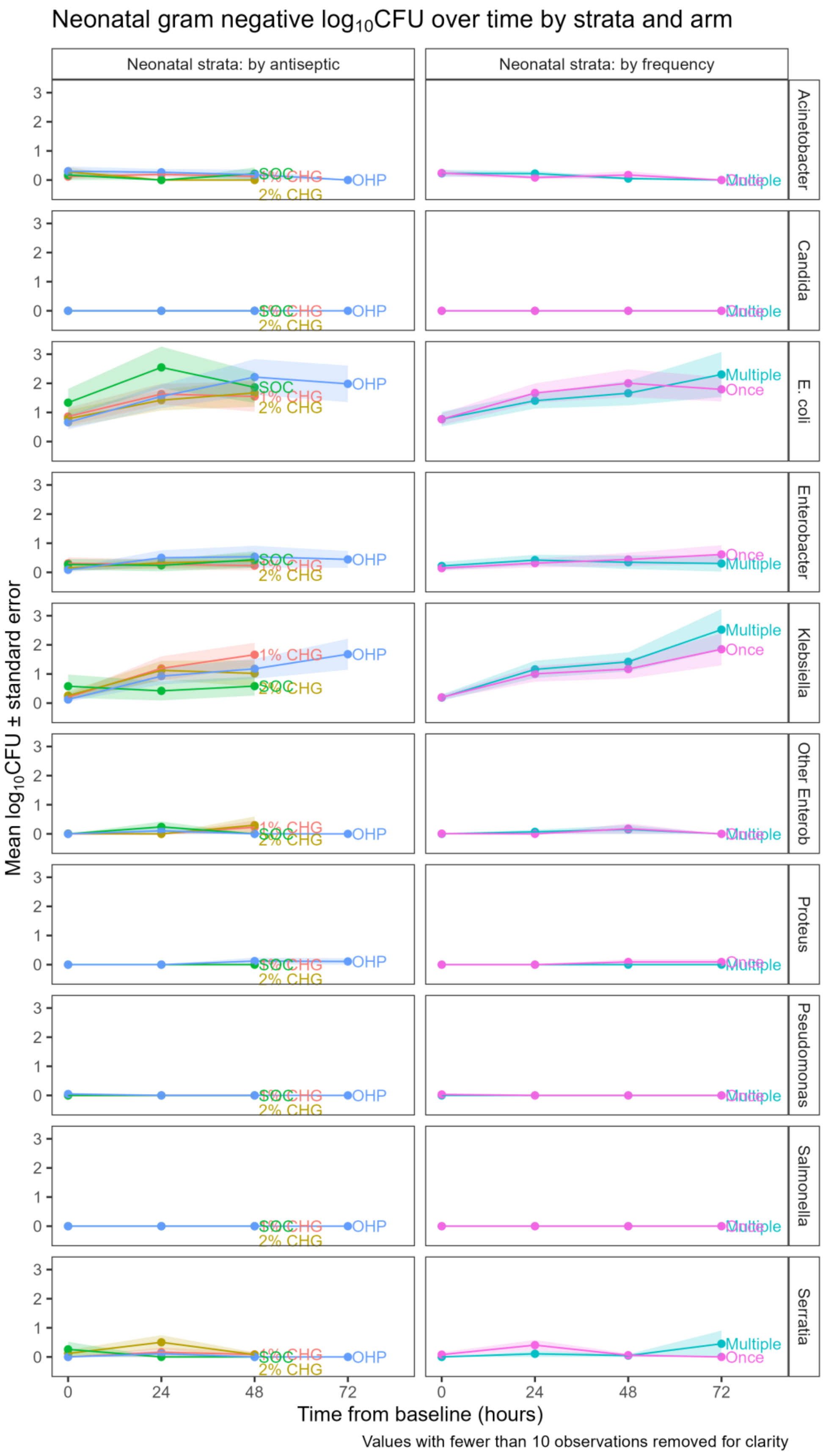

© 2026 Beales E et al. *JAMA Network Open*.

eTable 1. Additional baseline variables (maternal)

Variable	Overall	1% CHG (N=43)	2% CHG (N=43)	OHP (N=42)	Multiple (N=64)	Once (N=64)	SOC (N=21)
Gravidity (mean (SD))	2.35 (1.36)	2.44 (1.39)	2.33 (1.57)	2.36 (1.25)	2 (1.08)	2.75 (1.57)	2.19 (1.12)
Parity (mean (SD))	1.26 (1.36)	1.33 (1.38)	1.28 (1.59)	1.29 (1.22)	0.95 (1.09)	1.64 (1.58)	1.05 (1.12)
Gestational diabetes	1 (1%)	0 (0%)	0 (0%)	1 (2%)	0 (0%)	1 (2%)	0 (0%)
Cervical dilation at enrolment	2.06 (1.04)	2.3 (1.24)	1.81 (0.82)	2.1 (1.08)	2.17 (0.94)	1.97 (1.19)	2 (0.84)
Rupture of membranes at enrolment	13 (9%)	4 (9%)	4 (9%)	4 (10%)	6 (9%)	6 (9%)	1 (5%)
If ruptured, spontaneous	13 (100%)	4 (100%)	4 (100%)	4 (100%)	6 (100%)	6 (100%)	1 (100%)
Prolonged rupture of membranes	5 (38%)	2 (50%)	1 (25%)	2 (50%)	2 (33%)	3 (50%)	0 (0%)
Offensive liquor	0 (0%)	0 (0%)	0 (0%)	0 (0%)	0 (0%)	0 (0%)	0 (0%)

© 2026 Beales E et al. *JAMA Network Open*.

eTable 2. Additional baseline variables (neonatal)

Variable	Level	Overall	1% CHG (N=43)	2% CHG (N=43)	OHP (N=42)	Multiple (N=64)	Once (N=64)	SOC (N=21)
Prolonged rupture of membranes (>18h)	Yes	10 (7%)	3 (7%)	2 (5%)	5 (12%)	3 (5%)	7 (11%)	0 (0%)
	No	123 (84%)	36 (86%)	37 (88%)	32 (76%)	54 (86%)	51 (81%)	18 (86%)
	Unknown	14 (10%)	3 (7%)	3 (7%)	5 (12%)	6 (10%)	5 (8%)	3 (14%)
Offensive liquor	Yes	11 (7%)	2 (5%)	2 (5%)	4 (10%)	5 (8%)	3 (5%)	3 (14%)
	No	122 (83%)	36 (86%)	36 (86%)	34 (81%)	48 (76%)	58 (92%)	16 (76%)
	Unknown	14 (10%)	4 (10%)	4 (10%)	4 (10%)	10 (16%)	2 (3%)	2 (10%)
Mode of delivery	Vaginal spontaneous	76 (52%)	24 (57%)	18 (43%)	22 (52%)	35 (56%)	29 (46%)	12 (57%)
	Vaginal assisted	9 (6%)	2 (5%)	3 (7%)	3 (7%)	2 (3%)	6 (10%)	1 (5%)
	Emergency caesarean section	61 (41%)	15 (36%)	21 (50%)	17 (40%)	25 (40%)	28 (44%)	8 (38%)
	Unknown	1 (1%)	1 (2%)	0 (0%)	0 (0%)	1 (2%)	0 (0%)	0 (0%)
Maternal HIV status	Positive	11 (7%)	4 (10%)	3 (7%)	3 (7%)	5 (8%)	5 (8%)	1 (5%)
	Negative	131 (89%)	36 (86%)	36 (86%)	39 (93%)	56 (89%)	55 (87%)	20 (95%)
	Unknown	5 (3%)	2 (5%)	3 (7%)	0 (0%)	2 (3%)	3 (5%)	0 (0%)
APGAR Score at 1 minute	2	3 (2%)	2 (5%)	1 (2%)	0 (0%)	3 (5%)	0 (0%)	0 (0%)
	3	12 (8%)	3 (7%)	3 (7%)	5 (12%)	7 (11%)	4 (6%)	1 (5%)
	4	11 (7%)	4 (10%)	2 (5%)	3 (7%)	4 (6%)	5 (8%)	2 (10%)
	5	11 (7%)	2 (5%)	4 (10%)	3 (7%)	4 (6%)	5 (8%)	2 (10%)
	6	11 (7%)	1 (2%)	2 (5%)	4 (10%)	3 (5%)	4 (6%)	4 (19%)
	7	29 (20%)	6 (14%)	12 (29%)	9 (21%)	9 (14%)	18 (29%)	2 (10%)
	8	43 (29%)	15 (36%)	12 (29%)	11 (26%)	20 (32%)	18 (29%)	5 (24%)

© 2026 Beales E et al. *JAMA Network Open*.

Variable	Level	Overall	1% CHG (N=43)	2% CHG (N=43)	OHP (N=42)	Multiple (N=64)	Once (N=64)	SOC (N=21)
	9	24 (16%)	7 (17%)	6 (14%)	6 (14%)	10 (16%)	9 (14%)	5 (24%)
	Unknown	3 (2%)	2 (5%)	0 (0%)	1 (2%)	3 (5%)	0 (0%)	0 (0%)
APGAR Score at 5 minutes	4	1 (1%)	1 (2%)	0 (0%)	0 (0%)	1 (2%)	0 (0%)	0 (0%)
	5	10 (7%)	1 (2%)	3 (7%)	5 (12%)	8 (13%)	1 (2%)	1 (5%)
	6	12 (8%)	5 (12%)	3 (7%)	3 (7%)	4 (6%)	7 (11%)	1 (5%)
	7	12 (8%)	4 (10%)	3 (7%)	2 (5%)	5 (8%)	4 (6%)	3 (14%)
	8	14 (10%)	3 (7%)	4 (10%)	5 (12%)	4 (6%)	8 (13%)	2 (10%)
	9	16 (11%)	2 (5%)	8 (19%)	4 (10%)	5 (8%)	9 (14%)	2 (10%)
	10	79 (54%)	24 (57%)	21 (50%)	22 (52%)	33 (52%)	34 (54%)	12 (57%)
	Unknown	3 (2%)	2 (5%)	0 (0%)	1 (2%)	3 (5%)	0 (0%)	0 (0%)
APGAR Score at 10 minutes	4	1 (1%)	1 (2%)	0 (0%)	0 (0%)	1 (2%)	0 (0%)	0 (0%)
	5	1 (1%)	0 (0%)	1 (2%)	0 (0%)	1 (2%)	0 (0%)	0 (0%)
	6	2 (1%)	0 (0%)	1 (2%)	1 (2%)	2 (3%)	0 (0%)	0 (0%)
	7	13 (9%)	4 (10%)	4 (10%)	2 (5%)	6 (10%)	4 (6%)	3 (14%)
	8	12 (8%)	4 (10%)	1 (2%)	5 (12%)	5 (8%)	5 (8%)	2 (10%)
	9	9 (6%)	2 (5%)	4 (10%)	2 (5%)	2 (3%)	6 (10%)	1 (5%)
	10	79 (54%)	21 (50%)	25 (60%)	20 (48%)	31 (49%)	35 (56%)	13 (62%)
	Unknown	30 (20%)	10 (24%)	6 (14%)	12 (29%)	15 (24%)	13 (21%)	2 (10%)

© 2026 Beales E et al. *JAMA Network Open*.

eTable 3. Intervention delivery (maternal)

Comparison between antiseptics

Variable	Level/Metric	1% CHG (N=43)	2% CHG (N=43)	OHP (N=42)	SOC (N=21)	p-value	p-value drug only
Total number of antiseptic applications	median	1	1	1	0	<0.001	0.826
	(IQR)	(1, 2)	(1, 3)	(1, 3)	(0, 0)		
	N	43	43	42	21		
Number of antiseptic applications	0	1 (2%)	..	1 (2%)	21 (100%)	<0.001	0.77
	1	25 (58%)	24 (56%)	23 (55%)	..		
	2	6 (14%)	5 (12%)	3 (7%)	..		
	3	5 (12%)	8 (19%)	11 (26%)	..		
	4	4 (9%)	4 (9%)	1 (2%)	..		
	5	2 (5%)	1 (2%)	2 (5%)	..		
	6	..	1 (2%)	1 (2%)	..		
Last antiseptic application	0h	25 (58%)	24 (56%)	23 (55%)	..	<0.001	0.778
	4h	6 (14%)	5 (12%)	3 (7%)	..		
	8h	5 (12%)	8 (19%)	11 (26%)	..		
	24h	4 (9%)	4 (9%)	1 (2%)	..		
	28h	2 (5%)	1 (2%)	2 (5%)	..		
	32h	..	1 (2%)	1 (2%)	..		
	No antiseptic	1 (2%)	..	1 (2%)	21 (100%)		
Number of antiseptic applications before 4h swab	median	1	1	1	0	<0.001	n/a
	(IQR)	(1, 1)	(1, 1)	(1, 1)	(0, 0)		
	N	36	37	37	14		
Number of antiseptic applications before 8h swab	median	2	2	2	0	<0.001	0.885
	(IQR)	(1, 2)	(1, 2)	(1, 2)	(0, 0)		
	N	22	28	28	10		
Number of antiseptic applications before 24h swab	median	1	3	1	0	<0.001	0.342
	(IQR)	(1, 3)	(1, 3)	(1, 3)	(0, 0)		
	N	15	12	10	5		
Number of antiseptic applications before 28h swab	median	1	2	2	0	0.089	0.856
	(IQR)	(1, 3)	(2, 3)	(1, 4)	(0, 0)		
	N	6	2	6	4		
Number of antiseptic applications before 32h swab	median	1	5	5	0	<0.001	<0.001
	(IQR)	(1, 1)	(5, 5)	(5, 5)	(0, 0)		
	N	2	1	1	1		

© 2026 Beales E et al. *JAMA Network Open*.

Skin score assessed at all measured timepoints	39 (93%)	40 (93%)	41 (100%)	20 (100%)	0.322	0.278

Comparison between frequencies

Variable	Level/Metric	Once (N=64)	Multiple (N=64)	p-value
Total number of antiseptic applications	median	1	3	<0.001
	(IQR)	(1, 1)	(2, 3)	
	N	64	64	
Number of antiseptic applications	0	2 (3%)	..	<0.001
	1	62 (97%)	10 (16%)	
	2	..	14 (22%)	
	3	..	24 (38%)	
	4	..	9 (14%)	
	5	..	5 (8%)	
	6	..	2 (3%)	
Last antiseptic application	0h	62 (97%)	10 (16%)	<0.001
	4h	..	14 (22%)	
	8h	..	24 (38%)	
	24h	..	9 (14%)	
	28h	..	5 (8%)	
	32h	..	2 (3%)	
	No antiseptic	2 (3%)	..	
Number of antiseptic applications before 4h swab	median	1	1	n/a
	(IQR)	(1, 1)	(1, 1)	
	N	56	54	
Number of antiseptic applications before 8h swab	median	1	2	<0.001
	(IQR)	(1, 1)	(2, 2)	
	N	36	42	
Number of antiseptic applications before 24h swab	median	1	3	<0.001
	(IQR)	(1, 1)	(3, 3)	
	N	18	19	
Number of antiseptic applications before 28h swab	median	1	4	<0.001
	(IQR)	(1, 1)	(4, 4)	
	N	8	6	
Number of antiseptic applications before 32h swab	median	1	5	<0.001
	(IQR)	(1, 1)	(5, 5)	
	N	2	2	
Skin score assessed at all measured timepoints		60 (97%)	60 (94%)	0.418

© 2026 Beales E et al. *JAMA Network Open*.

eTable 4. Intervention delivery (neonatal)

Comparison between antiseptics

Variable	Level/Metric	1% CHG (N=42)	2% CHG (N=42)	OHP (N=42)	SOC (N=21)	p-value	p-value drug only
Total number of antiseptic applications	median	1	1	1	0	<0.001	0.734
	(IQR)	(1, 3)	(1, 2)	(1, 3)	(0, 0)		
	N	42	42	42	21		
Number of antiseptic applications	0	1 (2%)	..	..	21 (100%)	<0.001	0.619
	1	23 (55%)	22 (52%)	22 (52%)	..		
	2	5 (12%)	11 (26%)	6 (14%)	..		
	3	10 (24%)	6 (14%)	9 (21%)	..		
	4	3 (7%)	3 (7%)	5 (12%)	..		
Last antiseptic application	0h	22 (52%)	22 (52%)	22 (52%)	..	<0.001	0.809
	24h	6 (14%)	10 (24%)	6 (14%)	..		
	48h	10 (24%)	6 (14%)	9 (21%)	..		
	72h	3 (7%)	4 (10%)	5 (12%)	..		
	No antiseptic	1 (2%)	..	..	21 (100%)		
Number of antiseptic applications before 24h swab	median	1	1	1	0	<0.001	0.371
	(IQR)	(1, 1)	(1, 1)	(1, 1)	(0, 0)		
	N	39	39	39	20		
Number of antiseptic applications before 48h swab	median	2	1	2	0	<0.001	0.702
	(IQR)	(1, 2)	(1, 2)	(1, 2)	(0, 0)		
	N	24	21	26	14		
Number of antiseptic applications before 72h swab	median	2	1	1	0	<0.001	0.77
	(IQR)	(1, 3)	(1, 3)	(1, 3)	(0, 0)		
	N	6	9	15	9		
Skin score assessed before all antiseptic applications / swabs		41 (100%)	42 (100%)	42 (100%)	21 (100%)	n/a	n/a
Skin score assessed after all antiseptic applications		41 (100%)	42 (100%)	42 (100%)	..	n/a	n/a
Temperature assessed before all antiseptic applications / swabs		40 (98%)	42 (100%)	41 (98%)	21 (100%)	n/a	n/a
Temperature assessed after all antiseptic applications		41 (100%)	41 (98%)	42 (100%)	..	n/a	n/a

© 2026 Beales E et al. *JAMA Network Open*.

Comparison between frequencies

Variable	Level/Metric	Once (N=63)	Multiple (N=63)	p-value
Total number of antiseptic applications	median	1	3	<0.001
	(IQR)	(1, 1)	(2, 3)	
	N	63	63	
Number of antiseptic applications	0	..	1 (2%)	<0.001
	1	63 (100%)	4 (6%)	
	2	..	22 (35%)	
	3	..	25 (40%)	
	4	..	11 (17%)	
Last antiseptic application	0h	63 (100%)	3 (5%)	<0.001
	24h	..	22 (35%)	
	48h	..	25 (40%)	
	72h	..	12 (19%)	
	No antiseptic		1 (2%)	
Number of antiseptic applications before 24h swab	median	1	1	0.315
	(IQR)	(1, 1)	(1, 1)	
	N	59	58	
Number of antiseptic applications before 48h swab	median	1	2	<0.001
	(IQR)	(1, 1)	(2, 2)	
	N	35	36	
Number of antiseptic applications before 72h swab	median	1	3	<0.001
	(IQR)	(1, 1)	(3, 3)	
	N	18	12	
Skin score assessed before all antiseptic applications/swabs		63 (100%)	62 (100%)	n/a
Skin score assessed after all antiseptic applications		63 (100%)	62 (100%)	n/a
Temperature assessed before all antiseptic applications/swabs		61 (97%)	62 (100%)	n/a
Temperature assessed after all antiseptic applications		63 (100%)	61 (98%)	n/a

© 2026 Beales E et al. *JAMA Network Open*.

eTable 5. Primary and secondary outcomes (maternal)

Primary and key secondary outcomes with modelled effect sizes averaged across all observations days. Estimates are presented vs 1% CHG applied once.

Arm	Change baseline to 4h $\log_{10}$CFU mean (SD) [N]	Change baseline to 8h $\log_{10}$CFU mean (SD) [N]	Change baseline to 24h $\log_{10}$CFU mean (SD) [N]	Change baseline to 28h $\log_{10}$CFU mean (SD) [N]	Change baseline to $\log_{10}$CFU 32h mean (SD) [N]	Adjusted difference in $\log_{10}$CFU (95% CI)	p-value
Total $\log_{10}$ CFU							
1% CHG (N=43)	-4.5 (2.3) [35]	-4.3 (2.6) [22]	-2.5 (2.5) [15]	-4.1 (2.1) [6]	-4.7 (3.0) [2]	..	..
2% CHG (N=43)	-5.2 (2.2) [37]	-5.0 (2.7) [28]	-4.3 (3.1) [12]	-2.5 (1.0) [3]	-1.7 (NA) [1]	-0.6 (-1.4, 0.2)	0.12
OHP (N=42)	-2.7 (2.2) [36]	-3.1 (2.2) [27]	-2.4 (3.7) [10]	-3.3 (4.0) [6]	-7.6 (NA) [1]	1.7 (0.9, 2.5)	<0.0001
Once (N=64)	-4.3 (2.4) [56]	-3.7 (2.9) [36]	-2.2 (2.6) [18]	-2.3 (1.7) [8]	-4.7 (3.0) [2]	..	..
Multiple (N=64)	-4.0 (2.6) [52]	-4.6 (2.3) [41]	-3.8 (3.4) [19]	-4.7 (3.3) [7]	-4.6 (4.1) [2]	-0.4 (-1.1, 0.2)	0.20
SOC (N=21)	0.2 (1.4) [14]	-0.5 (1.7) [10]	-0.3 (2.3) [5]	0.5 (2.5) [4]	1.9 (NA) [1]	3.5 (2.4, 4.6)	<0.0001
Skin score							
1% CHG (N=43)	0.19 (0.52) [36]	0.09 (0.68) [22]	-0.21 (0.43) [14]	-0.50 (0.55) [6]	-0.50 (0.71) [2]		
2% CHG (N=43)	0.32 (0.53) [37]	0.22 (0.51) [27]	0.38 (0.65) [13]	0.00 (0.00) [3]	0.00 (NA) [1]	0.16 (-0.03, 0.35)	0.09
OHP (N=42)	0.46 (0.56) [37]	0.32 (0.55) [28]	0.00 (0.47) [10]	0.17 (0.41) [6]	1.00 (NA) [1]	0.26 (0.07, 0.45)	0.0067
Once (N=64)	0.30 (0.57) [56]	-0.03 (0.45) [36]	-0.16 (0.37) [19]	-0.25 (0.46) [8]	-0.50 (0.71) [2]		
Multiple (N=64)	0.35 (0.52) [54]	0.44 (0.59) [41]	0.28 (0.67) [18]	0.00 (0.58) [7]	0.50 (0.71) [2]	0.21 (0.06, 0.36)	0.0079
SOC (N=21)	0.07 (0.27) [14]	0.00 (0.00) [10]	0.00 (0.00) [5]	0.00 (0.00) [4]	0.00 (NA) [1]	-0.04 (-0.31, 0.22)	0.74
Neonate born to mother: Total $\log_{10}$ CFU							
1% CHG (N=43)	2.9 (3.1) [36]		..	..	..	..	..
2% CHG (N=43)	2.5 (2.6) [38]		..	..	..	-0.4 (-1.8, 1.0)	0.54
OHP (N=42)	4.4 (3.6) [37]		..	..	..	1.5 (0.1, 2.9)	0.038

© 2026 Beales E et al. *JAMA Network Open*.

Once (N=64)	3.3 (3.2) [56]		..	..	..	..	..
Multiple (N=64)	3.3 (3.3) [55]		..	..	..	-0.0 (-1.2, 1.1)	0.99
SOC (N=21)	3.5 (2.4) [17]		..	..	..	0.5 (-1.4, 2.4)	0.58
Gram negative log10 CFU							
1% CHG (N=43)	-2.5 (2.3) [35]	-2.5 (2.7) [22]	-1.5 (3.3) [15]	-2.7 (2.7) [6]	-2.2 (3.1) [2]	..	..
2% CHG (N=43)	-2.5 (2.4) [37]	-2.0 (2.9) [28]	-1.0 (2.6) [12]	-1.4 (1.5) [3]	-0.6 (NA) [1]	-0.1 (-0.8, 0.6)	0.72
OHP (N=42)	-1.8 (2.6) [36]	-1.9 (2.2) [27]	-0.4 (2.6) [10]	-1.7 (3.6) [6]	-2.7 (NA) [1]	1.1 (0.4, 1.8)	0.0023
Once (N=64)	-2.3 (2.3) [56]	-1.5 (2.7) [36]	-0.7 (2.7) [18]	-1.9 (2.0) [8]	-2.2 (3.1) [2]	..	..
Multiple (N=64)	-2.2 (2.6) [52]	-2.6 (2.4) [41]	-1.3 (3.0) [19]	-2.3 (3.7) [7]	-1.7 (1.5) [2]	-0.1 (-0.7, 0.5)	0.72
SOC (N=21)	-0.3 (1.7) [14]	0.1 (1.4) [10]	1.1 (1.5) [5]	2.1 (4.4) [4]	4.8 (NA) [1]	2.1 (1.1, 3.0)	<0.0001
Gram positive log10 CFU							
1% CHG (N=43)	-4.2 (2.2) [35]	-3.8 (2.4) [22]	-2.8 (2.7) [15]	-3.5 (1.5) [6]	-4.0 (2.1) [2]	..	
2% CHG (N=43)	-5.0 (1.9) [37]	-4.8 (2.5) [28]	-4.5 (3.0) [12]	-2.4 (1.0) [3]	-2.4 (NA) [1]	-0.5 (-1.2, 0.2)	0.17
OHP (N=42)	-2.7 (1.9) [36]	-3.5 (2.1) [27]	-2.9 (3.9) [10]	-4.5 (4.8) [6]	-8.7 (NA) [1]	1.2 (0.5, 2.0)	0.0013
Once (N=64)	-4.0 (2.1) [56]	-3.5 (2.6) [36]	-2.3 (2.5) [18]	-2.0 (1.5) [8]	-4.0 (2.1) [2]	..	
Multiple (N=64)	-3.9 (2.3) [52]	-4.5 (2.1) [41]	-4.5 (3.4) [19]	-5.6 (3.5) [7]	-5.5 (4.5) [2]	-0.6 (-1.2, 0.0)	0.063
SOC (N=21)	-0.3 (1.0) [14]	-1.1 (2.1) [10]	-1.9 (2.8) [5]	0.2 (2.3) [4]	-2.6 (NA) [1]	2.6 (1.6, 3.6)	<0.0001
Yeast log10CFU							
1% CHG (N=43)	0.2 (1.0) [35]	0.5 (1.8) [22]	0.5 (1.2) [15]	0.0 (0.0) [6]	1.3 (1.9) [2]	..	
2% CHG (N=43)	-0.4 (1.6) [37]	-0.4 (1.4) [28]	-0.1 (2.3) [12]	-0.3 (0.5) [3]	0.0 (NA) [1]	-0.6 (-1.1, -0.1)	0.03
OHP (N=42)	-0.1 (0.6) [36]	0.0 (1.2) [27]	-0.2 (0.5) [10]	-0.3 (0.6) [6]	0.0 (NA) [1]	-0.4 (-1.0, 0.1)	0.091
Once (N=64)	-0.0 (1.4) [56]	0.2 (1.8) [36]	-0.1 (1.8) [18]	0.0 (0.0) [8]	1.3 (1.9) [2]	..	
Multiple (N=64)	-0.2 (0.8) [52]	-0.2 (1.1) [41]	0.3 (1.2) [19]	-0.4 (0.6) [7]	0.0 (0.0) [2]	-0.2 (-0.6, 0.2)	0.31
SOC (N=21)	-0.1 (0.3) [14]	0.3 (1.7) [10]	0.3 (0.7) [5]	-1.6 (2.4) [4]	2.7 (NA) [1]	-0.4 (-1.1, 0.3)	0.29
Enterococcus spp. log10CFU							

© 2026 Beales E et al. *JAMA Network Open*.

1% CHG (N=43)	-2.3 (2.5) [35]	-2.3 (2.3) [22]	-2.0 (2.4) [15]	-2.7 (1.2) [6]	-2.1 (1.2) [2]	..	
2% CHG (N=43)	-2.5 (2.6) [37]	-2.3 (2.8) [28]	-2.2 (3.1) [12]	-1.7 (4.3) [3]	2.5 (NA) [1]	-0.1 (-0.8, 0.5)	0.64
OHP (N=42)	-1.6 (2.1) [36]	-2.1 (2.2) [27]	-1.5 (2.9) [10]	-2.3 (3.1) [6]	-2.3 (NA) [1]	0.6 (0.0, 1.3)	0.045
Once (N=64)	-2.2 (2.1) [56]	-1.8 (2.0) [36]	-1.3 (2.0) [18]	-2.1 (2.2) [8]	-2.1 (1.2) [2]	..	
Multiple (N=64)	-2.1 (2.7) [52]	-2.5 (2.8) [41]	-2.5 (3.2) [19]	-2.6 (3.1) [7]	0.1 (3.4) [2]	-0.2 (-0.7, 0.3)	0.43
SOC (N=21)	-1.2 (1.4) [14]	-0.9 (1.8) [10]	-1.5 (1.3) [5]	1.1 (0.9) [4]	-0.4 (NA) [1]	1.1 (0.2, 2.0)	0.012
S. aureus log10CFU							
1% CHG (N=43)	-0.1 (0.4) [35]	0.0 (0.0) [22]	0.0 (0.0) [15]	0.0 (0.0) [6]	0.0 (0.0) [2]	..	
2% CHG (N=43)	-0.3 (0.8) [37]	-0.3 (0.9) [28]	-0.3 (1.0) [12]	-1.1 (1.9) [3]	0.0 (NA) [1]	-0.0 (-0.1, 0.1)	0.82
OHP (N=42)	-0.1 (0.5) [36]	0.2 (0.6) [27]	0.0 (0.0) [10]	0.0 (0.0) [6]	0.0 (NA) [1]	0.1 (0.0, 0.1)	0.039
Once (N=64)	-0.1 (0.5) [56]	-0.0 (0.8) [36]	-0.2 (0.8) [18]	-0.4 (1.2) [8]	0.0 (0.0) [2]	..	
Multiple (N=64)	-0.2 (0.7) [52]	-0.1 (0.5) [41]	0.0 (0.0) [19]	0.0 (0.0) [7]	0.0 (0.0) [2]	-0.0 (-0.1, 0.0)	0.62
SOC (N=21)	0.1 (0.4) [14]	0.0 (0.0) [10]	0.0 (0.0) [5]	0.0 (0.0) [4]	0.0 (NA) [1]	0.0 (-0.1, 0.1)	0.38
GBS log10CFU							
1% CHG (N=43)	-0.3 (1.1) [35]	-0.3 (1.2) [22]	-0.2 (0.9) [15]	0.0 (0.0) [6]	0.0 (0.0) [2]	..	
2% CHG (N=43)	-0.4 (1.0) [37]	-0.3 (0.8) [28]	-0.6 (1.0) [12]	-1.0 (1.7) [3]	0.0 (NA) [1]	0.0 (-0.2, 0.2)	0.9
OHP (N=42)	-0.0 (1.2) [36]	-0.3 (2.0) [27]	-0.7 (3.9) [10]	-1.6 (4.0) [6]	-8.9 (NA) [1]	0.2 (0.0, 0.5)	0.038
Once (N=64)	-0.3 (1.4) [56]	-0.4 (1.2) [36]	-0.2 (1.6) [18]	-0.4 (1.0) [8]	0.0 (0.0) [2]	..	
Multiple (N=64)	-0.2 (0.7) [52]	-0.2 (1.6) [41]	-0.7 (2.5) [19]	-1.4 (3.7) [7]	-4.4 (6.3) [2]	-0.1 (-0.2, 0.1)	0.54
SOC (N=21)	0.2 (0.7) [14]	0.2 (0.5) [10]	0.0 (0.0) [5]	0.0 (0.0) [4]	0.0 (NA) [1]	0.2 (-0.1, 0.5)	0.26
Klebsiella spp. log10CFU							
1% CHG (N=43)	-0.1 (0.6) [35]	-0.2 (0.7) [22]	0.7 (1.8) [15]	0.2 (2.3) [6]	0.2 (0.2) [2]	..	
2% CHG (N=43)	-0.0 (0.2) [37]	0.1 (0.5) [28]	0.5 (1.3) [12]	0.0 (0.0) [3]	0.0 (NA) [1]	-0.0 (-0.4, 0.3)	0.97
OHP (N=42)	-0.1 (1.1) [36]	-0.4 (0.9) [27]	0.2 (1.6) [10]	-0.5 (0.9) [6]	-1.0 (NA) [1]	0.1 (-0.2, 0.5)	0.51
Once (N=64)	-0.1 (0.4) [56]	0.0 (0.6) [36]	0.8 (1.5) [18]	0.5 (1.5) [8]	0.2 (0.2) [2]	..	

© 2026 Beales E et al. *JAMA Network Open*.

Multiple (N=64)	-0.1 (1.0) [52]	-0.3 (0.8) [41]	0.2 (1.5) [19]	-0.8 (1.3) [7]	-0.5 (0.7) [2]	-0.1 (-0.4, 0.2)	0.62
SOC (N=21)	0.0 (0.8) [14]	1.1 (1.9) [10]	0.7 (1.6) [5]	1.4 (2.9) [4]	2.9 (NA) [1]	0.4 (-0.1, 0.9)	0.11
Serratia spp. log10CFU							
1% CHG (N=43)	-0.0 (0.3) [35]	0.0 (0.1) [22]	0.0 (0.0) [15]	0.0 (0.0) [6]	0.0 (0.0) [2]	..	
2% CHG (N=43)	-0.1 (0.5) [37]	-0.1 (0.6) [28]	-0.2 (0.8) [12]	-1.0 (1.7) [3]	-2.9 (NA) [1]	-0.0 (-0.0, 0.0)	0.071
OHP (N=42)	-0.1 (0.3) [36]	-0.1 (0.3) [27]	0.0 (0.0) [10]	0.0 (0.0) [6]	0.0 (NA) [1]	-0.0 (-0.0, 0.0)	0.07
Once (N=64)	-0.0 (0.2) [56]	-0.0 (0.1) [36]	0.0 (0.0) [18]	0.0 (0.0) [8]	0.0 (0.0) [2]	..	
Multiple (N=64)	-0.1 (0.5) [52]	-0.1 (0.5) [41]	-0.2 (0.7) [19]	-0.4 (1.1) [7]	-1.5 (2.1) [2]	-0.0 (-0.0, 0.0)	0.84
SOC (N=21)	0.0 (0.0) [14]	0.0 (0.0) [10]	0.0 (0.0) [5]	0.0 (0.0) [4]	0.0 (NA) [1]	-0.0 (-0.0, 0.0)	0.17
E. coli log10CFU							
1% CHG (N=43)	-1.4 (1.8) [35]	-1.5 (1.9) [22]	-0.5 (2.4) [15]	-2.1 (2.4) [6]	-1.3 (1.8) [2]	..	
2% CHG (N=43)	-0.9 (1.3) [37]	-0.6 (1.5) [28]	-0.5 (1.2) [12]	-0.2 (0.3) [3]	2.3 (NA) [1]	0.1 (-0.4, 0.6)	0.69
OHP (N=42)	-0.6 (2.3) [36]	-1.1 (2.3) [27]	-0.8 (3.6) [10]	-1.1 (4.2) [6]	0.0 (NA) [1]	0.8 (0.3, 1.3)	0.0034
Once (N=64)	-0.9 (1.6) [56]	-0.5 (1.6) [36]	0.1 (1.9) [18]	-1.0 (1.1) [8]	-1.3 (1.8) [2]	..	
Multiple (N=64)	-1.0 (2.0) [52]	-1.5 (2.1) [41]	-1.2 (2.7) [19]	-1.7 (4.4) [7]	1.2 (1.6) [2]	-0.2 (-0.6, 0.2)	0.36
SOC (N=21)	-0.4 (1.1) [14]	-0.2 (1.7) [10]	0.7 (1.0) [5]	3.2 (3.6) [4]	5.8 (NA) [1]	0.7 (-0.0, 1.4)	0.053
Enterobacter spp. log10CFU							
1% CHG (N=43)	-0.1 (0.4) [35]	0.0 (0.0) [22]	0.2 (0.8) [15]	0.0 (0.0) [6]	0.0 (0.0) [2]	..	
2% CHG (N=43)	-0.0 (0.2) [37]	-0.0 (0.3) [28]	-0.1 (0.4) [12]	-0.4 (0.8) [3]	0.3 (NA) [1]	-0.1 (-0.3, 0.2)	0.54
OHP (N=42)	-0.3 (1.1) [36]	-0.0 (0.8) [27]	0.8 (1.7) [10]	-0.3 (0.7) [6]	0.0 (NA) [1]	0.0 (-0.2, 0.3)	0.71
Once (N=64)	-0.1 (0.4) [56]	-0.0 (0.2) [36]	-0.1 (0.3) [18]	-0.2 (0.5) [8]	0.0 (0.0) [2]	..	
Multiple (N=64)	-0.2 (0.9) [52]	-0.0 (0.6) [41]	0.6 (1.4) [19]	-0.3 (0.7) [7]	0.2 (0.2) [2]	0.1 (-0.1, 0.3)	0.18
SOC (N=21)	-0.3 (1.0) [14]	-0.4 (1.1) [10]	-0.1 (0.2) [5]	0.0 (0.0) [4]	0.0 (NA) [1]	-0.2 (-0.4, 0.1)	0.29
Proteus spp. log10CFU							
1% CHG (N=43)	-0.0 (0.2) [35]	0.0 (0.0) [22]	0.0 (0.0) [15]	0.0 (0.0) [6]	0.0 (0.0) [2]	..	

© 2026 Beales E et al. *JAMA Network Open*.

2% CHG (N=43)	0.0 (0.0) [37]	0.0 (0.3) [28]	0.0 (0.0) [12]	0.0 (0.0) [3]	0.0 (NA) [1]	0.0 (-0.0, 0.0)	0.24
OHP (N=42)	0.0 (0.0) [36]	0.0 (0.0) [27]	0.0 (0.0) [10]	0.0 (0.0) [6]	0.0 (NA) [1]	-0.0 (-0.0, 0.0)	0.97
Once (N=64)	-0.0 (0.2) [56]	0.0 (0.0) [36]	0.0 (0.0) [18]	0.0 (0.0) [8]	0.0 (0.0) [2]	..	
Multiple (N=64)	0.0 (0.0) [52]	0.0 (0.2) [41]	0.0 (0.0) [19]	0.0 (0.0) [7]	0.0 (0.0) [2]	0.0 (-0.0, 0.0)	0.36
SOC (N=21)	0.0 (0.0) [14]	0.0 (0.0) [10]	0.0 (0.0) [5]	0.0 (0.0) [4]	0.0 (NA) [1]	0.0 (-0.0, 0.0)	0.8
Pseudomonas spp. log10CFU							
1% CHG (N=43)	-0.1 (0.6) [35]	-0.2 (0.7) [22]	-0.2 (0.6) [15]	0.0 (0.0) [6]	0.0 (0.0) [2]	..	
2% CHG (N=43)	-0.0 (0.2) [37]	0.0 (0.0) [28]	0.0 (0.0) [12]	0.0 (0.0) [3]	0.0 (NA) [1]	-0.0 (-0.0, 0.0)	0.42
OHP (N=42)	-0.1 (0.8) [36]	-0.2 (0.9) [27]	0.0 (0.0) [10]	0.0 (0.0) [6]	0.0 (NA) [1]	-0.0 (-0.0, 0.0)	0.2
Once (N=64)	-0.0 (0.2) [56]	0.0 (0.0) [36]	0.0 (0.0) [18]	0.0 (0.0) [8]	0.0 (0.0) [2]	..	
Multiple (N=64)	-0.2 (0.8) [52]	-0.2 (0.9) [41]	-0.1 (0.6) [19]	0.0 (0.0) [7]	0.0 (0.0) [2]	0.0 (-0.0, 0.0)	0.73
SOC (N=21)	0.0 (0.0) [14]	0.0 (0.0) [10]	0.0 (0.0) [5]	0.0 (0.0) [4]	0.0 (NA) [1]	-0.0 (-0.0, 0.0)	0.59
Acinetobacter spp. log10CFU							
1% CHG (N=43)	-1.3 (1.9) [35]	-1.1 (2.2) [22]	-1.8 (2.5) [15]	-1.9 (2.2) [6]	-2.4 (3.4) [2]	..	
2% CHG (N=43)	-1.9 (2.5) [37]	-1.9 (2.6) [28]	-1.2 (2.4) [12]	-1.4 (2.5) [3]	0.0 (NA) [1]	-0.2 (-0.6, 0.2)	0.32
OHP (N=42)	-1.4 (2.1) [36]	-1.2 (1.9) [27]	-1.2 (2.0) [10]	-1.5 (1.9) [6]	-3.8 (NA) [1]	0.1 (-0.3, 0.5)	0.73
Once (N=64)	-1.7 (2.2) [56]	-1.4 (2.2) [36]	-1.9 (2.3) [18]	-2.6 (2.0) [8]	-2.4 (3.4) [2]	..	
Multiple (N=64)	-1.4 (2.2) [52]	-1.5 (2.4) [41]	-1.1 (2.4) [19]	-0.5 (1.4) [7]	-1.9 (2.7) [2]	-0.0 (-0.3, 0.3)	0.89
SOC (N=21)	0.1 (1.6) [14]	0.1 (0.6) [10]	0.1 (0.3) [5]	-1.1 (2.2) [4]	0.0 (NA) [1]	1.5 (1.0, 2.1)	<0.0001

© 2026 Beales E et al. *JAMA Network Open*.

eTable 6. Primary and secondary outcomes (neonatal)

Primary and key secondary outcomes with modelled effect sizes averaged across all days. Estimates are presented vs 1% CHG applied once.

Arm	Change baseline to 24h mean (SD) [N]	Change baseline to 48h mean (SD) [N]	Change baseline to 72h mean (SD) [N]	Adjusted difference in log_{10}CFU (95% CI)	p-value
Total log_{10} CFU					
1% CHG (N=43)	1.1 (2.8) [39]	1.8 (3.3) [24]	3.5 (3.3) [6]	..	..
2% CHG (N=43)	0.9 (2.9) [37]	2.5 (2.9) [21]	2.7 (3.6) [9]	-0.2 (-1.1, 0.7)	0.63
OHP (N=42)	2.0 (3.8) [39]	3.5 (3.2) [26]	4.1 (2.9) [15]	0.7 (-0.2, 1.6)	0.11
Once (N=64)	1.5 (2.7) [58]	3.0 (3.3) [35]	4.7 (2.2) [18]	..	..
Multiple (N=64)	1.3 (3.7) [57]	2.3 (3.1) [36]	1.9 (3.5) [12]	-0.5 (-1.2, 0.2)	0.17
SOC (N=21)	2.0 (3.8) [20]	2.7 (3.5) [14]	3.8 (3.7) [9]	1.3 (0.2, 2.4)	0.017
Skin score					
1% CHG (N=43)	1.8 (2.8) [39]	2.1 (1.8) [24]	4.3 (1.9) [6]	..	..
2% CHG (N=43)	1.3 (2.8) [39]	2.2 (3.1) [21]	3.1 (1.9) [9]	-0.1 (-1.1, 0.8)	0.79
OHP (N=42)	1.5 (3.0) [39]	2.3 (3.4) [26]	2.3 (2.4) [15]	-0.3 (-1.2, 0.7)	0.59
Once (N=64)	1.6 (2.6) [59]	2.5 (3.3) [35]	2.4 (2.0) [18]	..	..
Multiple (N=64)	1.4 (3.1) [58]	1.9 (2.3) [36]	3.7 (2.4) [12]	-0.1 (-0.8, 0.7)	0.87
SOC (N=21)	0.4 (2.3) [20]	0.6 (4.0) [14]	1.4 (4.0) [9]	-0.9 (-2.1, 0.3)	0.14
Gram negative log_{10} CFU					
1% CHG (N=43)	-0.13 (0.47) [39]	-0.08 (0.58) [24]	0.00 (0.00) [7]	..	..
2% CHG (N=43)	-0.10 (0.38) [39]	-0.14 (0.36) [21]	-0.22 (0.44) [9]	0.02 (-0.15, 0.18)	0.86
OHP (N=42)	-0.05 (0.56) [39]	-0.12 (0.59) [26]	-0.13 (0.52) [15]	0.04 (-0.13, 0.20)	0.66
Once (N=64)	-0.15 (0.52) [59]	-0.17 (0.57) [35]	-0.16 (0.50) [19]	..	..
Multiple (N=64)	-0.03 (0.42) [58]	-0.06 (0.47) [36]	-0.08 (0.29) [12]	0.02 (-0.12, 0.16)	0.78
SOC (N=21)	-0.15 (0.37) [20]	-0.07 (0.27) [14]	-0.22 (0.44) [9]	-0.05 (-0.27, 0.16)	0.63
Gram positive log_{10} CFU					
1% CHG (N=43)	0.3 (3.3) [39]	1.7 (3.1) [24]	3.3 (2.9) [6]	..	
2% CHG (N=43)	0.3 (2.7) [39]	2.2 (2.6) [21]	2.1 (5.1) [9]	-0.5 (-1.4, 0.4)	0.29
OHP (N=42)	1.2 (3.6) [39]	3.5 (3.2) [26]	4.1 (3.2) [15]	0.8 (-0.1, 1.7)	0.096
Once (N=64)	0.9 (2.8) [59]	3.0 (3.0) [35]	4.6 (2.4) [18]	..	
Multiple (N=64)	0.3 (3.6) [58]	2.0 (3.1) [36]	1.4 (4.7) [12]	-0.6 (-1.3, 0.2)	0.12
SOC (N=21)	1.4 (3.6) [20]	2.7 (3.5) [14]	3.7 (3.6) [9]	1.3 (0.1, 2.4)	0.035
Yeast log_{10} CFU					
1% CHG (N=43)	0.1 (0.6) [39]	0.0 (0.0) [24]	0.0 (0.0) [6]	..	

© 2026 Beales E et al. *JAMA Network Open*.

2% CHG (N=43)	0.0 (0.2) [39]	0.0 (0.0) [21]	0.0 (0.0) [9]	-0.1 (-0.2, 0.1)	0.45
OHP (N=42)	0.0 (0.0) [39]	0.0 (0.0) [26]	0.0 (0.0) [15]	-0.1 (-0.2, 0.1)	0.32
Once (N=64)	0.1 (0.5) [59]	0.0 (0.0) [35]	0.0 (0.0) [18]	..	
Multiple (N=64)	0.1 (0.3) [58]	0.0 (0.0) [36]	0.0 (0.0) [12]	-0.0 (-0.1, 0.1)	0.87
SOC (N=21)	0.3 (1.1) [20]	0.0 (0.0) [14]	0.0 (0.0) [9]	0.0 (-0.1, 0.2)	0.57
Enterococcus spp. log_{10} CFU					
1% CHG (N=43)	0.6 (3.0) [39]	2.6 (3.1) [24]	4.7 (3.1) [6]	..	
2% CHG (N=43)	0.6 (2.7) [39]	2.3 (2.9) [21]	2.4 (5.6) [9]	-0.5 (-1.6, 0.5)	0.32
OHP (N=42)	0.8 (2.7) [39]	2.8 (3.1) [26]	3.3 (2.9) [15]	-0.2 (-1.3, 0.8)	0.65
Once (N=64)	0.9 (2.7) [59]	3.2 (2.9) [35]	4.3 (2.7) [18]	..	
Multiple (N=64)	0.4 (2.9) [58]	2.0 (3.1) [36]	1.9 (5.0) [12]	-0.5 (-1.4, 0.3)	0.21
SOC (N=21)	1.3 (4.5) [20]	2.3 (4.3) [14]	2.5 (4.3) [9]	-0.3 (-1.6, 1.1)	0.68
S. aureus log_{10} CFU					
1% CHG (N=43)	0.1 (0.6) [39]	0.3 (1.3) [24]	0.0 (0.0) [6]	..	
2% CHG (N=43)	-0.4 (1.1) [39]	-0.3 (1.4) [21]	-0.7 (1.5) [9]	-0.2 (-0.5, 0.2)	0.29
OHP (N=42)	0.0 (0.9) [39]	0.3 (1.5) [26]	0.3 (1.6) [15]	0.0 (-0.3, 0.4)	0.91
Once (N=64)	0.0 (1.0) [59]	0.2 (1.5) [35]	0.0 (1.8) [18]	..	
Multiple (N=64)	-0.2 (0.8) [58]	0.0 (1.3) [36]	-0.2 (0.6) [12]	-0.1 (-0.4, 0.2)	0.38
SOC (N=21)	-0.5 (1.2) [20]	-0.5 (1.1) [14]	-0.4 (1.1) [9]	-0.3 (-0.7, 0.1)	0.15
GBS log_{10} CFU					
1% CHG (N=43)	-0.1 (0.3) [39]	-0.1 (0.3) [24]	0.0 (0.0) [6]	..	
2% CHG (N=43)	0.0 (0.0) [39]	0.0 (0.0) [21]	0.0 (0.0) [9]	0.0 (-0.2, 0.2)	1
OHP (N=42)	0.0 (0.7) [39]	0.1 (0.8) [26]	0.2 (1.0) [15]	0.2 (0.0, 0.3)	0.037
Once (N=64)	0.1 (0.5) [59]	0.1 (0.5) [35]	0.2 (0.9) [18]	..	
Multiple (N=64)	-0.1 (0.3) [58]	-0.1 (0.4) [36]	0.0 (0.0) [12]	-0.1 (-0.2, 0.0)	0.19
SOC (N=21)	-0.7 (1.4) [20]	-0.8 (1.6) [14]	-0.8 (1.5) [9]	-0.0 (-0.2, 0.2)	0.69
Klebsiella spp. log_{10} CFU					
1% CHG (N=43)	1.0 (2.1) [39]	1.5 (2.0) [24]	3.8 (3.1) [6]	..	
2% CHG (N=43)	0.9 (1.7) [39]	1.0 (2.1) [21]	1.6 (2.1) [9]	-0.1 (-0.9, 0.6)	0.7
OHP (N=42)	0.8 (1.9) [39]	1.0 (1.9) [26]	1.5 (2.3) [15]	-0.4 (-1.1, 0.4)	0.34
Once (N=64)	0.8 (1.8) [59]	1.0 (2.0) [35]	1.7 (2.5) [18]	..	
Multiple (N=64)	1.0 (2.0) [58]	1.3 (2.0) [36]	2.5 (2.5) [12]	0.3 (-0.3, 0.9)	0.37
SOC (N=21)	-0.2 (1.2) [20]	0.2 (2.0) [14]	0.4 (2.6) [9]	-1.1 (-2.1, -0.1)	0.026
Serratia spp. log_{10} CFU					
1% CHG (N=43)	0.2 (1.0) [39]	0.1 (0.4) [24]	0.9 (2.2) [6]	..	
2% CHG (N=43)	0.4 (1.3) [39]	-0.2 (1.2) [21]	0.0 (0.0) [9]	0.1 (-0.2, 0.4)	0.57
OHP (N=42)	0.1 (0.7) [39]	0.0 (0.0) [26]	0.0 (0.0) [15]	-0.1 (-0.4, 0.1)	0.33
Once (N=64)	0.3 (1.2) [59]	-0.1 (0.9) [35]	0.0 (0.0) [18]	..	

© 2026 Beales E et al. *JAMA Network Open*.

Multiple (N=64)	0.1 (0.8) [58]	0.0 (0.3) [36]	0.5 (1.6) [12]	-0.1 (-0.3, 0.1)	0.42
SOC (N=21)	-0.3 (1.2) [20]	-0.4 (1.5) [14]	-0.6 (1.8) [9]	-0.3 (-0.6, 0.0)	0.089
E. coli log_{10} *CFU*					
1% CHG (N=43)	0.7 (1.8) [39]	1.0 (2.6) [24]	1.1 (2.2) [6]	..	
2% CHG (N=43)	0.6 (2.5) [39]	1.1 (2.0) [21]	1.8 (2.4) [9]	0.1 (-0.8, 1.0)	0.9
OHP (N=42)	0.9 (2.3) [39]	1.8 (2.9) [26]	1.7 (2.1) [15]	0.4 (-0.5, 1.3)	0.39
Once (N=64)	0.9 (2.2) [59]	1.3 (2.8) [35]	1.3 (1.9) [18]	..	
Multiple (N=64)	0.6 (2.3) [58]	1.3 (2.3) [36]	2.0 (2.6) [12]	-0.1 (-0.9, 0.6)	0.71
SOC (N=21)	1.2 (2.2) [20]	1.1 (2.3) [14]	2.2 (2.4) [9]	0.6 (-0.6, 1.7)	0.33
Enterobacter spp. log_{10} *CFU*					
1% CHG (N=43)	-0.0 (1.4) [39]	-0.1 (1.7) [24]	0.7 (1.3) [6]	..	
2% CHG (N=43)	0.2 (1.2) [39]	0.2 (1.4) [21]	0.5 (1.4) [9]	0.0 (-0.5, 0.5)	0.89
OHP (N=42)	0.4 (1.5) [39]	0.4 (2.0) [26]	0.2 (0.8) [15]	0.2 (-0.3, 0.7)	0.5
Once (N=64)	0.2 (1.1) [59]	0.2 (1.6) [35]	0.4 (1.2) [18]	..	
Multiple (N=64)	0.2 (1.6) [58]	0.1 (1.9) [36]	0.3 (1.0) [12]	0.0 (-0.4, 0.5)	0.81
SOC (N=21)	-0.0 (1.3) [20]	0.0 (1.6) [14]	0.0 (1.7) [9]	0.1 (-0.5, 0.7)	0.75
Proteus spp. log_{10} *CFU*					
1% CHG (N=43)	0.0 (0.0) [39]	0.0 (0.0) [24]	0.0 (0.0) [6]	..	
2% CHG (N=43)	0.0 (0.0) [39]	0.0 (0.0) [21]	0.0 (0.0) [9]	-0.0 (-0.1, 0.1)	0.97
OHP (N=42)	0.0 (0.0) [39]	0.1 (0.6) [26]	0.1 (0.4) [15]	0.0 (-0.0, 0.1)	0.23
Once (N=64)	0.0 (0.0) [59]	0.1 (0.5) [35]	0.1 (0.4) [18]	..	
Multiple (N=64)	0.0 (0.0) [58]	0.0 (0.0) [36]	0.0 (0.0) [12]	-0.0 (-0.1, 0.0)	0.29
SOC (N=21)	0.0 (0.0) [20]	0.0 (0.0) [14]	0.0 (0.0) [9]	-0.0 (-0.1, 0.1)	0.67
Acinetobacter spp. log_{10} *CFU*					
1% CHG (N=43)	0.1 (0.7) [39]	0.1 (0.6) [24]	-0.3 (0.7) [6]	..	
2% CHG (N=43)	-0.3 (1.1) [39]	-0.2 (0.8) [21]	-0.1 (0.3) [9]	-0.2 (-0.3, 0.0)	0.096
OHP (N=42)	0.0 (1.0) [39]	-0.1 (1.5) [26]	-0.2 (0.6) [15]	0.0 (-0.2, 0.2)	0.73
Once (N=64)	-0.1 (0.9) [59]	0.0 (1.0) [35]	-0.3 (0.7) [18]	..	
Multiple (N=64)	-0.0 (0.9) [58]	-0.2 (1.1) [36]	0.0 (0.0) [12]	0.0 (-0.1, 0.2)	0.66
SOC (N=21)	-0.2 (0.8) [20]	-0.0 (1.3) [14]	-0.1 (1.6) [9]	0.0 (-0.2, 0.2)	0.99

© 2026 Beales E et al. *JAMA Network Open*.

eTable 7. Main effects interaction analyses (maternal)

Interactions between main terms included in models for change total log10CFU (primary outcome)

Effect 1	Effect 2	p-value of interaction
drug	frequency	0.6102
drug	time	0.6152
frequency	time	0.1690

p-values from interactions fitted one at a time to the focal model

© 2026 Beales E et al. *JAMA Network Open*.

eTable 8. Main effects interaction analyses (neonatal)

Interactions between main terms included in models for change total log10CFU (primary outcome)

Effect 1	Effect 2	p-value of interaction
drug	frequency	0.1369
drug	time	0.9163
frequency	time	0.0194

p-values from interactions fitted one at a time to the focal model. Drug:time interaction added to the model did not converge so unstructured residuals were removed.

Frequency:time interaction added to the model:

Effect	Estimate	lower 95% CI	upper 95% CI	p-value	p-value of factor
2% CHG	-0.27	-1.18	0.635	0.556	0.0616
OHP	0.648	-0.188	1.48	0.127	
Multiple	0.747	-0.59	2.08	0.271	
SOC	1.22	0.171	2.27	0.023	
hour	0.0679	0.0424	0.0934	<0.001	
Multiple#hour	-0.0294	-0.0565	-0.00229	0.034	

CHG = chlorhexidine; OHP = octenidine 0.1% combined with phenoxyethanol 2%; *SOC = standard of care*

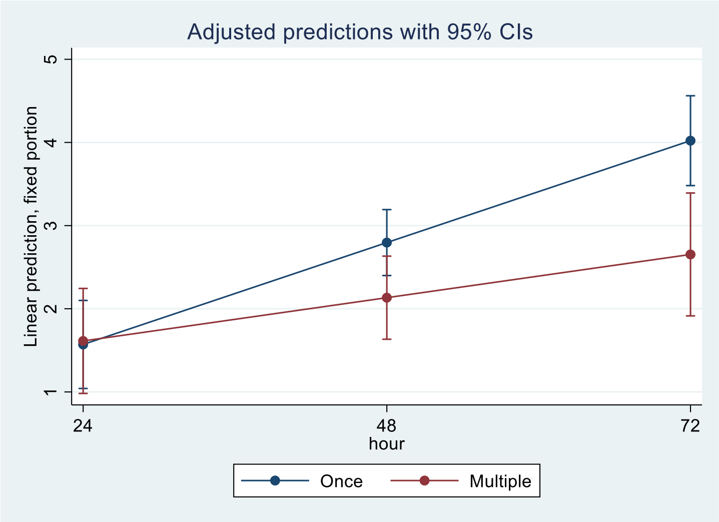

© 2026 Beales E et al. *JAMA Network Open*.

eTable 9. Subgroup analyses (maternal)

Effects of baseline factors on total log10CFU and effect modification of intervention effects

Variable	Effect/ Interaction	p-value of effect/interaction
HIV status	Main effect	0.1166
	Drug interaction	0.6464
	Frequency interaction	0.2136
Antibiotics since admission at baseline	Main effect	0.5730
	Drug interaction (without 2% CHG as none found)	0.4444
	Frequency interaction	0.1897
Rupture of membranes at baseline	Main effect	0.2373
	Drug interaction	0.7838
	Frequency interaction	0.6670
Prolonged rupture of membranes at enrolment	Main effect	0.9917
	Drug interaction	0.4183
	Frequency interaction	0.4398

Neonates born to mothers in the maternal population

Variable	Effect/ Interaction	p-value of effect/interaction
HIV status	Main effect	0.0349
	Drug interaction	0.9116
	Frequency interaction	0.6582
Mode of delivery	Main effect (forceps/vacuum assisted removed as observed only once)	0.0243
	Drug interaction (forceps/vacuum assisted removed as observed only once)	0.7680
	Frequency interaction (forceps/vacuum assisted removed as observed only once)	0.6104

p-values with specified subgroups measured at baseline and interaction terms added to the focal model one at a time.
HIV status coefficient = -1.9 (95% CI: -3.7, -0.1) for positive (lower) vs negative (higher)
Model of delivery coefficient = 1.7 (95% CI: 0.2, 3.2) for spontaneous vaginal (higher) vs Emergency c-section (lower)
HIV status coefficient for spontaneous vaginal only = -2.5 (95% CI: -4.7, -0.4; p = 0.020) for positive (lower) vs negative (higher)
HIV = human immunodeficiency virus

© 2026 Beales E et al. *JAMA Network Open*.

eTable 10. Subgroup analyses (neonatal)

Effects of baseline factors on total log10CFU and effect modification of intervention effects

Variable	Effect/ Interaction	p-value of effect/interaction
Maternal HIV status	Main effect	0.0260
	Drug interaction (with 9 unknown HIV status removed)	0.1201
	Frequency interaction	0.1379
Mother received antibiotics in labor	Main effect	0.3412
	Drug interaction	0.8815
	Frequency interaction	0.2687
Prolonged rupture of membranes	Main effect	0.2685
	Drug interaction*	0.0917
	Frequency interaction	0.2872
Offensive liquor	Main effect	0.4161
	Drug interaction	0.2649
	Frequency interaction	0.8107
Gestational age at enrolment	Main effect	0.624
	Drug interaction*	0.9358
	Frequency interaction	0.9017
Admission ward	Main effect	0.0398
	Drug interaction	0.0959
	Frequency interaction	0.0160
Antibiotics before enrolment	Main effect	0.0033
	Drug interaction	0.0655
	Frequency interaction*	0.0315
CHG cord care	Main effect	0.6937
	Drug interaction	0.4390
	Frequency interaction	0.8032

Values with specified subgroups measured at baseline and interaction terms added to the focal model one at a time.
*Unstructured residuals excluded from the model due to model not converging.
HIV status coefficient = 1.5 (95% CI: 0.2, 2.9) for positive (higher) vs negative (lower)
CHG = chlorhexidine; HIV = human immunodeficiency virus

ADMISSION WARD # FREQUENCY INTERACTION

Effect	Estimate	lower 95% CI	upper 95% CI	p-value	p-value of factor
2% CHG	-0.264	-1.16	0.633	0.562	0.0584
OHP	0.662	-0.173	1.5	0.119	
Multiple	0.997	-0.411	2.4	0.163	
SOC	1.28	0.221	2.34	0.018	

© 2026 Beales E et al. *JAMA Network Open*.

Nursery	0.00216	-1.14	1.14	0.997
Nursery HDU	-0.0101	-1.11	1.09	0.985
Nursery#multiple	-2.34	-4.11	-0.571	0.01
Nursery HDU# multiple	-1.77	-3.48	-0.066	0.042

Note: reference ward was Postnatal ward with mother. Postnatal ward with mother 66 (25%), Nursery 90 (33%), Nursery HDU 113 (42%).
CHG = chlorhexidine; HDU = high dependency unit; OHP = octenidine 0.1% combined with phenoxyethanol 2%; *SOC = standard of care*

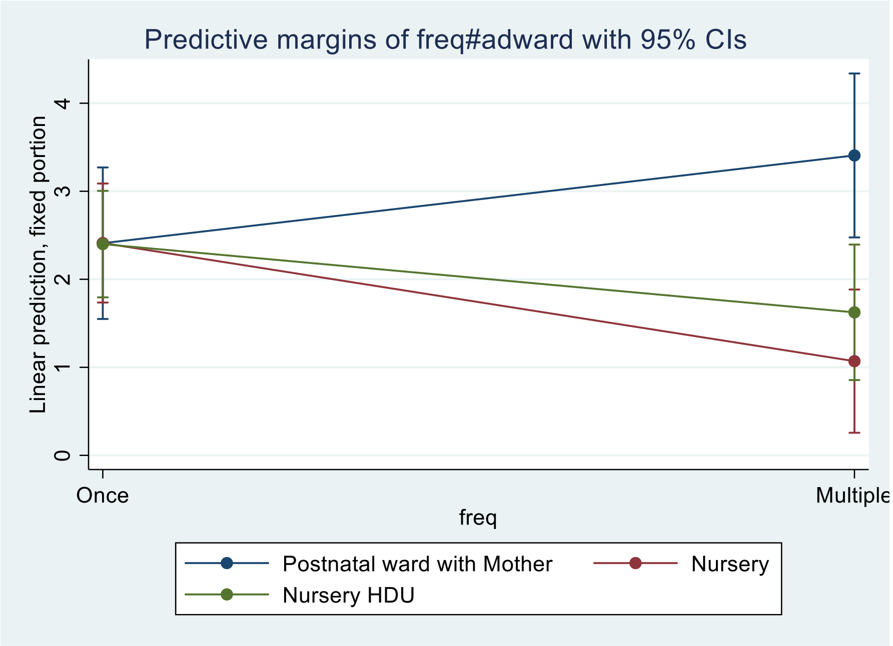

ANTIBIOTICS BEFORE ENROLMENT # FREQUENCY INTERACTION

Effect	Estimate	lower 95% CI	upper 95% CI	p-value
2% CHG	-0.142	-1	0.719	0.745
OHP	0.834	-0.00628	1.67	0.052
Multiple	0.171	-0.601	0.942	0.662
SOC	1.51	0.439	2.59	0.006
Antibiotics before enrolment	-0.314	-1.33	0.7	0.54
Antibiotics before enrolment#Multiple	-1.68	-3.22	-0.131	0.034

Antibiotics before enrolment: 65 (24%)
CHG = chlorhexidine; OHP = octenidine 0.1% combined with phenoxyethanol 2%; *SOC = standard of care*

© 2026 Beales E et al. *JAMA Network Open*.

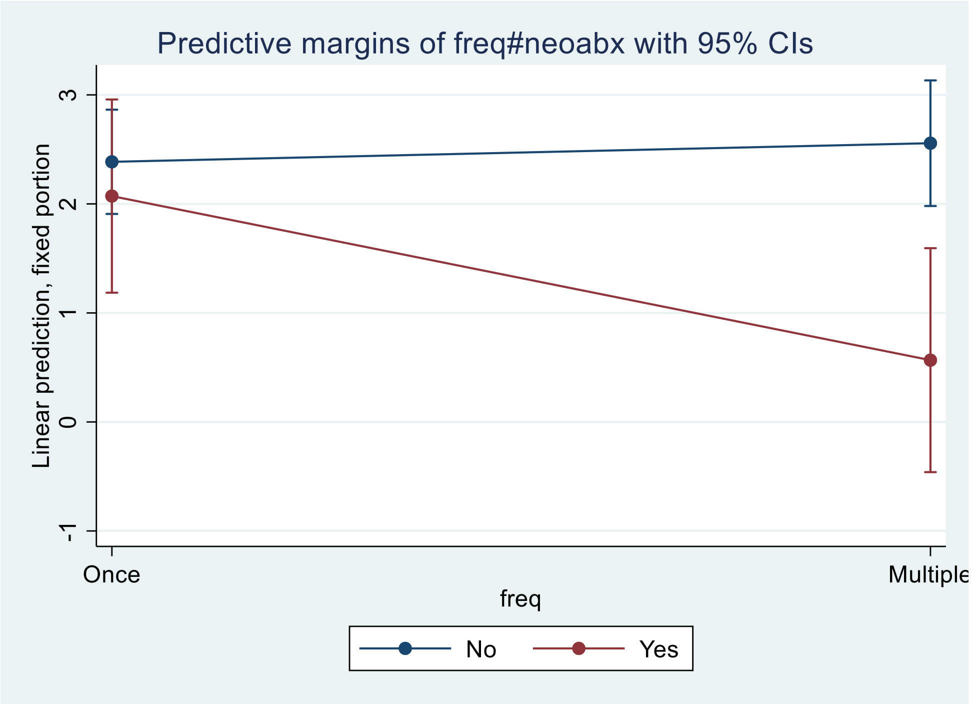

© 2026 Beales E et al. *JAMA Network Open*.

eTable 11. ACCEPT posterior probability analyses (maternal)

Probability treatment effect compared to 1% CHG/once is more negative than acceptability threshold under different priors. E.g. under non-informative priors

- Probability 2% CHG is better than 1% CHG is 94%, and the probability is better by at least 0.5 log_{10}CFU is 62%
- Probability OHP is better than 1% CHG is 0%
- Probability multiple is better than once is 90%, and probably is better by at least 0.5 log_{10}CFU is 39%
- Probability SOC is better than 1% CHG once is 0%

Factor	Acceptability threshold	Non-informative	Optimistic	Sceptical
2% CHG	-2	0%	2%	0%
	-1	17%	79%	61%
	-0.5	62%	99%	96%
	0	94%	100%	100%
	0.5	100%	100%	100%
	1	100%	100%	100%
	2	100%	100%	100%
OHP	-2	0%	0%	0%
	-1	0%	0%	0%
	-0.5	0%	0%	0%
	0	0%	2%	0%
	0.5	0%	26%	12%
	1	5%	79%	60%
	2	80%	100%	100%
Multiple applications	-2	0%	0%	0%
	-1	4%	7%	2%
	-0.5	39%	68%	45%
	0	90%	100%	97%
	0.5	100%	100%	100%
	1	100%	100%	100%
	2	100%	100%	100%
SOC	-2	0%	0%	0%
	-1	0%	0%	0%
	-0.5	0%	0%	0%
	0	0%	0%	0%
	0.5	0%	0%	3%
	1	0%	12%	32%
	2	0%	97%	99%

CHG = chlorhexidine; OHP = octenidine 0.1% combined with phenoxyethanol 2%; *SOC = standard of care*

© 2026 Beales E et al. *JAMA Network Open*.

eTable 12. ACCEPT posterior probability analyses (neonatal)

- Probability 2% CHG is better than 1% CHG is 63%, and the probability is better by at least 0.5 log_{10}CFU is 22%
- Probability OHP is better than 1% CHG is 4%
- Probability multiple is better than once is 72%, and probably is better by at least 0.5 log_{10}CFU is 22%.
- Probability SOC is better than 1% CHG once is 1%.

See the Statistical Analysis Plan for further details of priors

Factor	Acceptability threshold	Non-informative	Optimistic	Sceptical
2% CHG	-2	0%	0%	0%
	-1	3%	21%	4%
	-0.5	22%	74%	40%
	0	63%	99%	89%
	0.5	93%	100%	100%
	1	99%	100%	100%
	2	100%	100%	100%
OHP	-2	0%	0%	0%
	-1	0%	0%	0%
	-0.5	0%	5%	1%
	0	4%	45%	16%
	0.5	24%	91%	68%
	1	67%	100%	97%
	2	100%	100%	100%
Multiple applications	-2	0%	0%	0%
	-1	2%	0%	0%
	-0.5	22%	36%	9%
	0	72%	97%	79%
	0.5	97%	100%	100%
	1	100%	100%	100%
	2	100%	100%	100%
SOC	-2	0%	0%	0%
	-1	0%	0%	0%
	-0.5	0%	0%	0%
	0	1%	1%	8%
	0.5	4%	32%	70%
	1	20%	92%	99%
	2	82%	100%	100%

CHG = chlorhexidine; OHP = octenidine 0.1% combined with phenoxyethanol 2%; *SOC = standard of care*

eTable 13. Sensitivity analyses (maternal)

All analyses are as per the primary analysis either restricted to one sample only (and day of sampling removed from the model) or with additional factors added. Estimates are versus the **1% CHG, applied once** reference group from a normal linear regression, adjusted for arm, s, and time of sample where required.

REPEATED SAMPLES SENSITIVITY ANALYSIS

First outcome measure after baseline

Effect	Estimate	lower 95% CI	upper 95% CI	p-value	p-value of factor
2% CHG	-0.608	-1.42	0.204	0.141	<0.001
OHP	2.09	1.28	2.91	<0.001	
Multiple	0.241	-0.423	0.905	0.473	
SOC	4.5	3.37	5.63	<0.001	

First swab timepoint: 4h: 123 (100%)
CHG = chlorhexidine; OHP = octenidine 0.1% combined with phenoxyethanol 2%; *SOC = standard of care*

Final outcome measure

Effect	Estimate	lower 95% CI	upper 95% CI	p-value	p-value of factor
2% CHG	-0.78	-1.76	0.197	0.116	<0.001
OHP	1.14	0.15	2.12	0.024	
Multiple	-0.777	-1.57	0.0204	0.056	
SOC	3.1	1.75	4.46	<0.001	

CHG = chlorhexidine; OHP = octenidine 0.1% combined with phenoxyethanol 2%; *SOC = standard of care*

© 2026 Beales E et al. *JAMA Network Open*.

eTable 14. Sensitivity analyses (neonatal)

All analyses are as per the primary analysis either restricted to one sample only (and day of sampling removed from the model) or with additional factors added. Estimates are versus the **1% CHG, applied once** reference group from a normal linear regression, adjusted for arm, s, and time of sample where required.

First outcome measure after baseline

Effect	Estimate	lower 95% CI	upper 95% CI	p-value	p-value of factor
2% CHG	-0.287	-1.37	0.796	0.601	0.2283
OHP	0.638	-0.44	1.72	0.244	
Multiple	0.161	-0.726	1.05	0.72	
SOC	1.76	0.375	3.15	0.013	

CHG = chlorhexidine; OHP = octenidine 0.1% combined with phenoxyethanol 2%; *SOC = standard of care*

Final outcome measure

Effect	Estimate	lower 95% CI	upper 95% CI	p-value	p-value of factor
2% CHG	0.252	-0.658	1.16	0.585	0.0745
OHP	1.02	0.109	1.93	0.029	
Multiple	-0.0888	-0.833	0.656	0.814	
SOC	1.8	0.623	2.98	0.003	

CHG = chlorhexidine; OHP = octenidine 0.1% combined with phenoxyethanol 2%; *SOC = standard of care*

© 2026 Beales E et al. *JAMA Network Open*.

eTable 15. SAE summary tables (maternal)

SAEs are presented as N (%) M where N is the number of participants experiencing at least one SAE, % is the associated percentage of participants and M is the total number of events.

Maternal population

(a) by randomization to antiseptic or SOC

Variable	Overall (N=149)	1% CHG (N=43)	2% CHG (N=43)	OHP (N=42)	SOC (N=21)
Overall SAEs	7 (5%) 7	3 (7%) 3	2 (5%) 2	1 (2%) 1	1 (5%) 1
Death	5 (3%) 5	2 (5%) 2	1 (2%) 1	1 (2%) 1	1 (5%) 1
Prolonged hospitalization	2 (1%) 2	1 (2%) 1	1 (2%) 1	0 (0%) 0	0 (0%) 0
Congenital Anomaly	1 (1%) 1	0 (0%) 0	1 (2%) 1	0 (0%) 0	0 (0%) 0

CHG = chlorhexidine; OHP = octenidine 0.1% combined with phenoxyethanol 2%; *SAE = serious adverse event; SOC = standard of care*

(b) by randomization frequency (excluding SOC)

Variable	Once (N=64)	Multiple (N=64)
Overall SAEs	4 (6%) 4	2 (3%) 2
Death	2 (3%) 2	2 (3%) 2
Prolonged hospitalization	2 (3%) 2	0 (0%) 0
Congenital Anomaly	1 (2%) 1	0 (0%) 0

ANALYSIS OF OVERALL SAEs

Arm	Odds ratio estimate	lower 95% CI	upper 95% CI	p-value
CHG 2%	0.66	0.05	6.11	1
OHP	0.33	0.01	4.32	0.640
Multiple	0.49	0.04	3.55	0.689
SOC	0.50	0.01	7.33	0.99

Estimates showing comparison to 1% CHG, applied once from an exact logistic regression.
CHG = chlorhexidine; OHP = octenidine 0.1% combined with phenoxyethanol 2%*; SAE = serious adverse event; SOC = standard of care*

SAEs by PT

(a) by randomization to antiseptic or SOC

Variable	1% CHG (N=43)	2% CHG (N=43)	OHP (N=42)	SOC (N=21)
Congenital, familial and genetic disorders /Congenital anomaly	0 (0%) 0	1 (2%) 1	0 (0%) 0	0 (0%) 0

© 2026 Beales E et al. *JAMA Network Open*.

Variable	1% CHG (N=43)	2% CHG (N=43)	OHP (N=42)	SOC (N=21)
Infections and infestations /Sepsis neonatal	0 (0%) 0	0 (0%) 0	1 (2%) 1	1 (5%) 1
Pregnancy, puerperium and perinatal conditions /Foetal death	1 (2%) 1	0 (0%) 0	0 (0%) 0	0 (0%) 0
Pregnancy, puerperium and perinatal conditions /Postpartum haemorrhage	1 (2%) 1	0 (0%) 0	0 (0%) 0	0 (0%) 0
Pregnancy, puerperium and perinatal conditions /Stillbirth	0 (0%) 0	1 (2%) 1	0 (0%) 0	0 (0%) 0
Respiratory, thoracic and mediastinal disorders /Neonatal asphyxia	1 (2%) 1	0 (0%) 0	0 (0%) 0	0 (0%) 0

CHG = chlorhexidine; OHP = octenidine 0.1% combined with phenoxyethanol 2%*; SAE = serious adverse event; SOC = standard of care*

(b) by randomization frequency (excluding SOC)

Variable	Once (N=64)	Multiple (N=64)
Congenital, familial and genetic disorders /Congenital anomaly	1 (2%) 1	0 (0%) 0
Infections and infestations /Sepsis neonatal	1 (2%) 1	0 (0%) 0
Pregnancy, puerperium and perinatal conditions /Foetal death	1 (2%) 1	0 (0%) 0
Pregnancy, puerperium and perinatal conditions /Postpartum haemorrhage	1 (2%) 1	0 (0%) 0
Pregnancy, puerperium and perinatal conditions /Stillbirth	0 (0%) 0	1 (2%) 1
Respiratory, thoracic and mediastinal disorders /Neonatal asphyxia	0 (0%) 0	1 (2%) 1

eTable 16. SAE summary tables (neonatal)

Neonatal population

(a) by randomization to antiseptic or SOC

**Variable**	**Overall (N=147)**	**1% CHG (N=42)**	**2% CHG (N=42)**	**OHP (N=42)**	**SOC (N=21)**
Overall SAEs	16 (11%) 17	4 (10%) 4	7 (17%) 8	2 (5%) 2	3 (14%) 3
Death	9 (6%) 9	2 (5%) 2	3 (7%) 3	1 (2%) 1	3 (14%) 3
Life threatening	3 (2%) 3	1 (2%) 1	1 (2%) 1	0 (0%) 0	1 (5%) 1
Prolonged hospitalization	7 (5%) 8	2 (5%) 2	3 (7%) 4	1 (2%) 1	1 (5%) 1

CHG = chlorhexidine; OHP = octenidine 0.1% combined with phenoxyethanol 2%; *SAE = serious adverse event; SOC = standard of care*

(b) by randomization frequency (excluding SOC)

**Variable**	**Once (N=63)**	**Multiple (N=63)**
Overall SAEs	5 (8%) 6	8 (13%) 8
Death	2 (3%) 2	4 (6%) 4
Life threatening	1 (2%) 1	1 (2%) 1
Prolonged hospitalization	2 (3%) 3	4 (6%) 4

© 2026 Beales E et al. *JAMA Network Open*.

ANALYSIS OF OVERALL SAEs

Arm	Odds ratio estimate	lower 95% CI	upper 95% CI	p-value
CHG 2%	1.88	0.43	9.54	0.520
OHP	0.48	0.04	3.57	0.676
Multiple	1.6	0.45	7.03	0.558
SOC	2.05	0.23	16.74	0.691

CHG = chlorhexidine; OHP = octenidine 0.1% combined with phenoxyethanol 2%; *SAE = serious adverse event; SOC = standard of care*

(a) by randomization to antiseptic or SOC

Variable	1% CHG (N=42)	2% CHG (N=42)	OHP (N=42)	SOC (N=21)
Infections and infestations /Pneumonia	0 (0%) 0	2 (5%) 2	0 (0%) 0	0 (0%) 0
Infections and infestations /Sepsis neonatal	3 (7%) 3	3 (7%) 3	1 (2%) 1	1 (5%) 1
Pregnancy, puerperium and perinatal conditions /Hypoxic ischaemic encephalopathy neonatal	0 (0%) 0	3 (7%) 3	0 (0%) 0	1 (5%) 1
Pregnancy, puerperium and perinatal conditions /Neonatal asphyxia	1 (2%) 1	0 (0%) 0	1 (2%) 1	0 (0%) 0
Respiratory, thoracic and mediastinal disorders /Neonatal respiratory distress syndrome	0 (0%) 0	0 (0%) 0	0 (0%) 0	1 (5%) 1

(b) by randomization frequency (excluding SOC)

Variable	Once (N=63)	Multiple (N=63)
Infections and infestations /Pneumonia	1 (2%) 1	1 (2%) 1
Infections and infestations /Sepsis neonatal	3 (5%) 3	4 (6%) 4
Pregnancy, puerperium and perinatal conditions /Hypoxic ischaemic encephalopathy neonatal	2 (3%) 2	1 (2%) 1
Pregnancy, puerperium and perinatal conditions /Neonatal asphyxia	0 (0%) 0	2 (3%) 2
Respiratory, thoracic and mediastinal disorders /Neonatal respiratory distress syndrome	0 (0%) 0	0 (0%) 0

© 2026 Beales E et al. *JAMA Network Open*.

eTable 17. SAE line listing (maternal)

Note: no mothers experienced multiple SAEs

SAE Ref	Trial Arm	Main Diagnosis	Severity	Relatedness	Event Outcome
1	CHG 1% Multiple	Birth asphyxia	Grade 5 (Death)	Unrelated	Death
2	CHG 1% Once	Post partum haemorrhage	Grade 2 (Moderate)	Unrelated	Resolved
3	CHG 1% Once	Neonatal Intrauterine Death (fresh still birth) due to missed Transverse Lie	Grade 5 (Death)	Unrelated	Death
4	SOC	Neonatal Sepsis	Grade 5 (Death)	Unrelated	Death
5	CHG 2% Once	Congenital Abnormality	Grade 2 (Moderate)	Unrelated	Resolved with sequelae
6	OHP Once	Neonatal sepsis	Grade 5 (Death)	Unlikely to be related	Death
7	CHG 2% Multiple	Stillbirth	Grade 5 (Death)	Unrelated	Death

CHG = chlorhexidine; IMP = investigational medicinal product; OHP = octenidine 0.1% combined with phenoxyethanol 2%; *SAE = serious adverse event; SOC = standard of care*

© 2026 Beales E et al. *JAMA Network Open*.

eTable 18. SAE line listing (neonatal)

SAE Ref	SAE num	Trial Arm	Main Diagnosis	Severity	Relatedness	Event Outcome
1	1	CHG 2% Once	Pneumonia	Grade 2 (Moderate)	Unrelated	Resolved
2	1	CHG 2% Multiple	Neonatal sepsis	Grade 2 (Moderate)	Unrelated	Resolved
3	1	OHP Multiple	Neonatal sepsis	Grade 2 (Moderate)	Unrelated	Resolved
4	1	SOC	Neonatal Sepsis	Grade 5 (Death)	Unrelated	Death
5	1	CHG 2% Once	Neonatal sepsis	Grade 3 (Severe)	Unrelated	Resolved
5	2	CHG 2% Once	hypoxic ischaemic encephalopathy	Grade 3 (Severe)	Unrelated	Resolved with sequalae
6	1	CHG 1% Multiple	Birth asphyxia	Grade 5 (Death)	Unrelated	Death
7	1	CHG 2% Once	hypoxic ischaemic encephalopathy	Grade 4 (Life-threatening)	Unrelated	Death
8	1	CHG 1% Multiple	Neonatal sepsis (Klebsiella pneumoniae)	Grade 2 (Moderate)	Unrelated	Death
9	1	CHG 1% Once	Neonatal sepsis	Grade 5 (Death)	Unrelated	Death
10	1	SOC	Respiratory distress syndrome	Grade 5 (Death)	Unrelated	Death
11	1	SOC	Neonatal death secondary to severe hypoxic ischaemic encephalopathy	Grade 5 (Death)	Unrelated	Death

CHG = chlorhexidine; IMP = investigational medicinal product; OHP = octenidine 0.1% combined with phenoxyethanol 2%; *SAE = serious adverse event; SOC = standard of care*

© 2026 Beales E et al. *JAMA Network Open*.

eReferences

1. Lund CH, Osborne JW. Validity and reliability of the Neonatal Skin Condition score. *JOGNN - Journal of Obstetric, Gynecologic, and Neonatal Nursing*. 2004;33(3):320–327.

2. Harwood-Nuss A, Wolfson A LC. *The Clinical Practice of Emergency Medicine*. (Lippincott-Raven;, ed.).; 1996.

3. U.S. Department of Health and Human Services. National Cancer Institute Common Terminology Criteria for Adverse Events (CTCAE). Version 5. https://www.ctc.ucl.ac.uk/TrialDocuments/Uploaded/Common%20Terminology%20Criteria%20for%20Adverse%20Events%20(CTCAE)%20v5.0_14092023_0.pdf. Published November 27, 2017. Accessed August 7, 2025.

4. IMPAACT P1106: Pharmacokinetic Characteristics of Antiretrovirals and Tuberculosis Medicines in Low Birth Weight Infants. https://clinicaltrials.gov/study/NCT02383849. Accessed August 7, 2025.

© 2026 Beales E et al. *JAMA Network Open*.
