# Supplementary material for: Antiseptic Cleansing to Reduce Vertical Transmission of Pathogens to Neonates: The NeoVT-AMR Randomized Clinical Trial
Source: JAMA Netw Open. 2026 Jun 11;9(6):e2615665. doi: 10.1001/jamanetworkopen.2026.15665 (PMC13261496; doi:10.1001/jamanetworkopen.2026.15665)
Supplement: Supplement 3. — Data Sharing Statement [file jamanetwopen-e2615665-s003.pdf]

Data Sharing Statement

Beales. Antiseptic Cleansing to Reduce Vertical Transmission of Pathogens to Neonates. *JAMA Netw Open*. Published June 11, 2026. doi:10.1001/jamanetworkopen.2026.15665

Data
Additional Information: NeoVT-AMR is registered on the ISRCTN registry - registration number: ISRCTN78026255. The registration record can be found at: https://doi.org/10.1186/ISRCTN78026255
Data available: Yes
Data types: Deidentified participant data, Data dictionary
How to access data: lhill@citystgeorges.ac.uk
When available: With publication

Supporting Documents
Document types: None

Additional Information
Who can access the data: Data will be freely available on publication
Types of analyses: Data will be freely available on publication
Mechanisms of data availability: Data will be freely available on publication
